# Adding 6 months of androgen deprivation therapy to postoperative radiotherapy for prostate cancer: a comparison of short-course versus no androgen deprivation therapy in the RADICALS-HD randomised controlled trial

**DOI:** 10.1016/S0140-6736(24)00548-8

**Published:** 2024-05-16

**Authors:** Chris C Parker, Noel W Clarke, Adrian D Cook, Howard Kynaston, Charles N Catton, William R Cross, Peter M Petersen, Rajendra A Persad, Fred Saad, Lorna C Bower, John Logue, Heather Payne, Silvia Forcat, Cindy Goldstein, Claire Murphy, Juliette Anderson, Maroie Barkati, David M Bottomley, Jennifer Branagan, Ananya Choudhury, Peter W M Chung, Lyn Cogley, Chee L Goh, Peter Hoskin, Vincent Khoo, Shawn C Malone, Lindsey Masters, Stephen L Morris, Abdenour Nabid, Aldrich D Ong, Rakesh Raman, Kathryn L Tarver, Alison C Tree, Jane Worlding, James P Wylie, Anjali M Zarkar, Wendy R Parulekar, Mahesh K B Parmar, Matthew R Sydes, on behalf of the RADICALS investigators

**Affiliations:** The Royal Marsden NHS Foundation Trust, London, UK; The Institute of Cancer Research, London, UK; Department of Urology, The Christie NHS Foundation Trust, Manchester, UK; Division of Cancer Sciences, University of Manchester, Manchester, UK; Department of Urology, Salford Royal Hospital, Salford, UK; MRC Clinical Trials Unit at UCL, Institute of Clinical Trials and Methodology, University College London, London, UK; Division of Cancer and Genetics, Cardiff University Medical School, Cardiff, UK; Princess Margaret Cancer Centre, University Health Network, Toronto, ON, Canada; Department of Urology; St James’s University Hospital, Leeds, UK; Department of Oncology, Rigshospitalet, University of Copenhagen, Copenhagen, Denmark; Department of Urology, Bristol Urological Institute, Bristol, UK; Department of Urology; The Royal Marsden NHS Foundation Trust, London, UK; The Institute of Cancer Research, London, UK; Centre Hospitalier de l’Université de Montréal, Montreal, QC, Canada; Guy’s and St Thomas’ NHS Foundation Trust, London, UK; Department of Urology, The Christie NHS Foundation Trust, Manchester, UK; The Prostate Centre, London, UK; MRC Clinical Trials Unit at UCL, Institute of Clinical Trials and Methodology, University College London, London, UK; Department of Clinical Oncology; Department of Radiation Oncology; Department of Clinical Oncology; Northampton General Hospital, Northampton, UK; Department of Urology, The Christie NHS Foundation Trust, Manchester, UK; Division of Cancer Sciences, University of Manchester, Manchester, UK; Princess Margaret Cancer Centre, University Health Network, Toronto, ON, Canada; Department of Radiation Oncology, University of Toronto, Toronto, ON, Canada; Derriford Hospital, Plymouth, UK; Royal Surrey Hospital, Guildford, UK; Division of Cancer Sciences, University of Manchester, Manchester, UK; Mount Vernon Cancer Centre, Northwood, UK; The Royal Marsden NHS Foundation Trust, London, UK; The Institute of Cancer Research, London, UK; The Ottawa Hospital, University of Ottawa, Ottawa, ON, Canada; MRC Clinical Trials Unit at UCL, Institute of Clinical Trials and Methodology, University College London, London, UK; Centre Hospitalier de l’Université de Montréal, Montreal, QC, Canada; Guy’s and St Thomas’ NHS Foundation Trust, London, UK; Service de Radio-Oncologie, Centre Hospitalier Universitaire de Sherbrooke, Sherbrooke, QC, Canada; Max Rady Faculty of Health Sciences, University of Manitoba, Winnipeg, MB, Canada; Kent Oncology Centre, Kent and Canterbury Hospital, Canterbury, UK; Department of Oncology, Queen’s Hospital, Romford, UK; The Royal Marsden NHS Foundation Trust, London, UK; The Institute of Cancer Research, London, UK; University Hospitals Coventry and Warwickshire NHS Trust, Coventry, UK; Department of Urology, The Christie NHS Foundation Trust, Manchester, UK; Department of Oncology, University Hospitals Birmingham, Birmingham, UK; Canadian Cancer Trials Group, Queen’s University, Kingston, ON, Canada; MRC Clinical Trials Unit at UCL, Institute of Clinical Trials and Methodology, University College London, London, UK; MRC Clinical Trials Unit at UCL, Institute of Clinical Trials and Methodology, University College London, London, UK

## Abstract

**Background:**

Previous evidence indicates that adjuvant, short-course androgen deprivation therapy (ADT) improves metastasis-free survival when given with primary radiotherapy for intermediate-risk and high-risk localised prostate cancer. However, the value of ADT with postoperative radiotherapy after radical prostatectomy is unclear.

**Methods:**

RADICALS-HD was an international randomised controlled trial to test the efficacy of ADT used in combination with postoperative radiotherapy for prostate cancer. Key eligibility criteria were indication for radiotherapy after radical prostatectomy for prostate cancer, prostate-specific antigen less than 5 ng/mL, absence of metastatic disease, and written consent. Participants were randomly assigned (1:1) to radiotherapy alone (no ADT) or radiotherapy with 6 months of ADT (short-course ADT), using monthly subcutaneous gonadotropin-releasing hormone analogue injections, daily oral bicalutamide monotherapy 150 mg, or monthly subcutaneous degarelix. Randomisation was done centrally through minimisation with a random element, stratified by Gleason score, positive margins, radiotherapy timing, planned radiotherapy schedule, and planned type of ADT, in a computerised system. The allocated treatment was not masked. The primary outcome measure was metastasis-free survival, defined as distant metastasis arising from prostate cancer or death from any cause. Standard survival analysis methods were used, accounting for randomisation stratification factors. The trial had 80% power with two-sided α of 5% to detect an absolute increase in 10-year metastasis-free survival from 80% to 86% (hazard ratio [HR] 0·67). Analyses followed the intention-to-treat principle. The trial is registered with the ISRCTN registry, ISRCTN40814031, and ClinicalTrials.gov, NCT00541047.

**Findings:**

Between Nov 22, 2007, and June 29, 2015, 1480 patients (median age 66 years [IQR 61–69]) were randomly assigned to receive no ADT (n=737) or short-course ADT (n=743) in addition to postoperative radiotherapy at 121 centres in Canada, Denmark, Ireland, and the UK. With a median follow-up of 9·0 years (IQR 7·1–10·1), metastasis-free survival events were reported for 268 participants (142 in the no ADT group and 126 in the short-course ADT group; HR 0·886 [95% CI 0·688–1·140], p=0·35). 10-year metastasis-free survival was 79·2% (95% CI 75·4–82·5) in the no ADT group and 80·4% (76·6–83·6) in the short-course ADT group. Toxicity of grade 3 or higher was reported for 121 (17%) of 737 participants in the no ADT group and 100 (14%) of 743 in the short-course ADT group (p=0·15), with no treatment-related deaths.

**Interpretation:**

Metastatic disease is uncommon following postoperative bed radiotherapy after radical prostatectomy. Adding 6 months of ADT to this radiotherapy did not improve metastasis-free survival compared with no ADT. These findings do not support the use of short-course ADT with postoperative radiotherapy in this patient population.

**Funding:**

Cancer Research UK, UK Research and Innovation (formerly Medical Research Council), and Canadian Cancer Society.

## Introduction

In patients receiving radiotherapy as initial treatment for localised prostate cancer, short-course androgen deprivation therapy (ADT) improves metastasis-free survival and overall survival, and is a standard of care for those with intermediate-risk and high-risk clinically localised disease.^1^ In patients receiving postoperative radiotherapy after radical prostatectomy, the role of short-course ADT is less certain.

Two randomised controlled trials, GETUG-AFU 16 and RTOG 0534, have previously tested the addition of short-course ADT to salvage radiotherapy to the prostate bed.^2,3^ Both trials found evidence in favour of adding short-course ADT in terms of freedom from progression, their primary outcome measure, although that outcome measure also included prostate-specific antigen (PSA) progression. ADT will inevitably delay PSA progression, and that alone should not be sufficient to change practice. GETUG-AFU 16 also reported a statistically significant improvement in metastasis-free survival (hazard ratio [HR] 0·73, 95% CI 0·54–0·98; p=0·034), whereas RTOG 0534 found no good evidence of an improvement in time to metastasis (0·78, 0·52–1·17; p=0·083). Neither trial has yet reported clear evidence of benefit for adding short-course ADT in terms of cause-specific survival or overall survival.

Given the known morbidity of ADT,^4^ together with its uncertain long-term benefits in this setting, there has been no consensus on the use of short-course ADT in patients receiving postoperative radiotherapy after previous radical prostatectomy. Previous surveys of UK urological and oncological specialists found variable use of ADT. Of those responding urologists who recommend adjuvant treatment, 72% recommended radiotherapy alone; when ADT was used, 31% recommended short-term treatment (3–12 months) and 44% recommended either short-term or long-term treatment depending on the patient.^5,6^ The relevant clinical guidelines still make only weak recommendations; the ESMO Clinical Practice Guidelines for prostate cancer state that “ADT for 6 months…may be offered to men having salvage RT [radiotherapy]”.^7^ We conducted a large, international, randomised controlled trial to test the efficacy of short-course ADT used in combination with postoperative radiotherapy to the prostate bed. Given the limitations of PSA-based outcome measures in any trial of ADT,^8^ we used metastasis-free survival as the primary outcome measure.

## Methods

### Study design and participants

RADICALS was an international, phase 3, multicentre, open-label, randomised controlled trial in prostate cancer. The protocol addressed questions of the timing of radiotherapy after surgery and the use of ADT with postoperative radiotherapy across separate randomisations with overlapping patient groups.

RADICALS-HD recruited consenting patients with prostate localised cancer due for radiotherapy at any time after previous radical prostatectomy for prostatic adenocarcinoma. The exclusion criteria were previous pelvic radiotherapy, preoperative ADT for longer than 8 months, any ADT within 6 months before surgery, PSA greater than 5 ng/mL, metastatic disease, other active malignancy likely to interfere with protocol treatment or follow-up, or any postoperative hormone therapy. There were no age restrictions. Appropriate ethical review was in place for each participating country (appendix p 8). All participants gave written informed consent. The protocol is available online. This study is registered with the ISRCTN registry, ISRCTN40814031, and with ClinicalTrials.gov, NCT00541047.

### Randomisation and masking

Participants in the none-versus-short comparison of RADICALS-HD were randomly allocated to radiotherapy alone (no ADT) or radiotherapy with the addition of 6 months of ADT (short-course ADT). Site staff engaged patients about potential participation in the trial. Those who decided to participate were given the choice, with their clinical team, of being randomly assigned three-way 1:1:1 between no ADT, short-course ADT, and long-course ADT (adding 24 months of ADT), or two-way 1:1 between no ADT and short-course ADT or between short-course ADT and long-course ADT. The large majority of participants chose to be randomly assigned in one of the two-way comparisons, although a small minority of participants chose to be randomly assigned in the three-way comparison. Patients who were allocated to long-course ADT are not included in this analysis because of the small number of participants in the three-way randomisation. Recruitment and random allocation of participants was implemented centrally using minimisation with a random element, stratified by Gleason score, positive margins, radiotherapy timing, planned radiotherapy schedule, and planned type of ADT, in a computerised system. The allocated treatment was open label.

### Procedures

If allocated, ADT was to be initiated as soon as possible, and certainly within 2 months after randomisation, and continued for 6 months. Participants were given ADT using gonadotropin-releasing hormone analogue therapy according to site choice using their indicated route, usually subcutaneously, with once-monthly injections recommended. This was supplemented by 3 weeks of an anti-androgen started 1 week before the first gonadotropin-releasing hormone analogue therapy administration. Outside of Canada, daily oral bicalutamide monotherapy 150 mg or monthly subcutaneous degarelix (with nationally approved dosing) were acceptable alternatives. Dose reductions were not possible; treatment could be stopped early if indicated.

Radiotherapy was started approximately 2 months after starting ADT. The intended radiotherapy schedule was prespecified for each participant as 52·5 Gy in 20 fractions over 4 weeks or 66·0 Gy in 33 fractions over 6·5 weeks. The radiotherapy target volume was to include the prostate bed and could also include pelvic lymph nodes. Detailed radiotherapy guidance was given in the protocol.

Scheduled follow-up was every 4 months for the first 2 years after randomisation, then every 6 months up to 5 years, and annually thereafter. PSA measurements were taken at every follow-up appointment and as clinically indicated. Imaging was done according to routine clinical practice and was reported locally, without masking of treatment allocation. There was no central review of imaging.

Clinician-reported data were collected at each follow-up visit on diarrhoea, proctitis, cystitis, haematuria, and urethral stricture, graded according to Radiation Therapy Oncology Group toxicity score.^9^ Data for other adverse events were collected, and graded for severity if the event met the criteria to be classified as a serious adverse event.

An algorithm was used to identify deaths with uncertain cause, using the reported primary and contributory causes of death together with disease history during the trial.^10^ The process did not refer to allocated treatment. These uncertain causes of death of trial participants were centrally adjudicated by one of three clinical members of the Trial Management Group (CCP, NWC, or CNC). Additionally, for patients in England and Wales, national death registration data were used to confirm deaths and the information contributed to the reviews.

### Outcome measures

The primary outcome measure was metastasis-free survival, defined as distant metastasis arising from prostate cancer or death from any cause. Extra-pelvic nodal disease was considered to be metastatic. Secondary outcome measures were freedom from distant metastasis (distant metastasis or death from prostate cancer), overall survival (death from any cause), initiation of non-protocol ADT (ADT other than the policy randomly assigned), clinical progression-free survival (local or nodal progression, metastases, non-protocol ADT, or death from prostate cancer), freedom from treatment failure (PSA progression when on ADT), toxicity, and patient-reported outcome measures (PROMs). An additional secondary outcome of treatment failure, defined as PSA progression when on ADT, was not well reported by sites and is not presented. PROMs were collected only in the subset of people also in the RADICALS-RT trial;^11,12^ this included a small number of people, so PROMs are not analysed here.

Safety analyses were done according to allocated treatment, categorised as Radiation Therapy Oncology Group grade 1, 2, or 3 or higher, and analysed with a χ^2^ test. Worst grade of adverse events includes routine-assessed toxicities and serious adverse events.

### Statistical analysis

This comparison was originally designed as part of a three-way comparison with disease-specific survival as the primary outcome measure and an overall recruitment target of 3053 patients across three arms. However, recruitment was permitted between pairs of arms to facilitate recruitment in the pilot phase and this method was subsequently maintained. Therefore, in 2010, the trial was repowered for separate comparisons of short-course ADT versus long-course ADT (reported separately^13^) and no ADT versus short-course ADT. This was done without any reference to accumulating comparative data within the trial. This separated, pairwise comparison required approximately 1263 patients to observe 128 events.

After recruitment and treatment had been completed for all participants, the primary outcome measure was changed in 2019 to metastasis-free survival. This change followed new evidence from the ICECaP study^14^ that metastasis-free survival was a robust early surrogate outcome measure for disease-specific survival. This decision did not involve anyone privy to accumulating comparative data from RADICALS-HD.^15^ With 200 events from the 1480 participants, this final design had 80% power with two-sided α of 5% to detect an increase in 10-year metastasis-free survival from 80% to 86% (HR=0·67). Based on previous experiences with long-term trials, the calculations conservatively assumed that follow-up might be truncated early between 5 years and 10 years after randomisation for up to 30% of participants.

The full statistical analysis plan is published elsewhere^10^ and the details are summarised here. All analyses followed the intention-to-treat principle, with analyses according to allocated group, and were conducted in Stata version 17.0. Follow-up was estimated through reverse censoring on death. For time-to-event outcome measures, the statistical significance of differences between groups was evaluated with the log-rank test, stratified by randomisation stratification factors. Effect estimates were obtained from Cox regression models, also stratified by randomisation stratification factors. The Grambsch–Therneau test was used to test the proportional hazards assumption, with restricted mean survival time becoming the primary estimate of effect if non-proportional hazards were detected, with time restricted (t*) to 10 years. Time-to-event graphs were presented in KMunicate format.^16^ When a formal date for stopping ADT was not reported, patients were not included in summaries of time on treatment. Competing risks regression was used for prostate cancer-specific survival with other causes of death as competing risks. p<0·05 was considered to indicate statistical significance.

Two prespecified subgroup analyses were planned, by pre-radiotherapy PSA value and by Charlson Comorbidity Index score (excluding age), testing for interaction in the treatment effect.^17^ It was hypothesised that patients with higher pre-radiotherapy PSA, and those with less comorbidity, would benefit more from ADT. Exploratory subgroup analysis of all randomisation stratification factors was also planned.

The Independent Data Monitoring Committee (IDMC) met to review data from RADICALS on ten occasions. There were no formal stopping guidelines; the IDMC were asked to give advice on whether the accumulating data from the trial, together with results from other relevant trials, justified continuing recruitment of further patients or further follow-up. The IDMC did not recommend stopping the trial early.

### Role of the funding source

The funders of the study had no role in study design (other than organising initial peer review by independent reviewers), data collection, data analysis, data interpretation, or writing of the report. The sponsor took responsibility for these elements, delegated through their staff.

## Results

**Figure 1:**
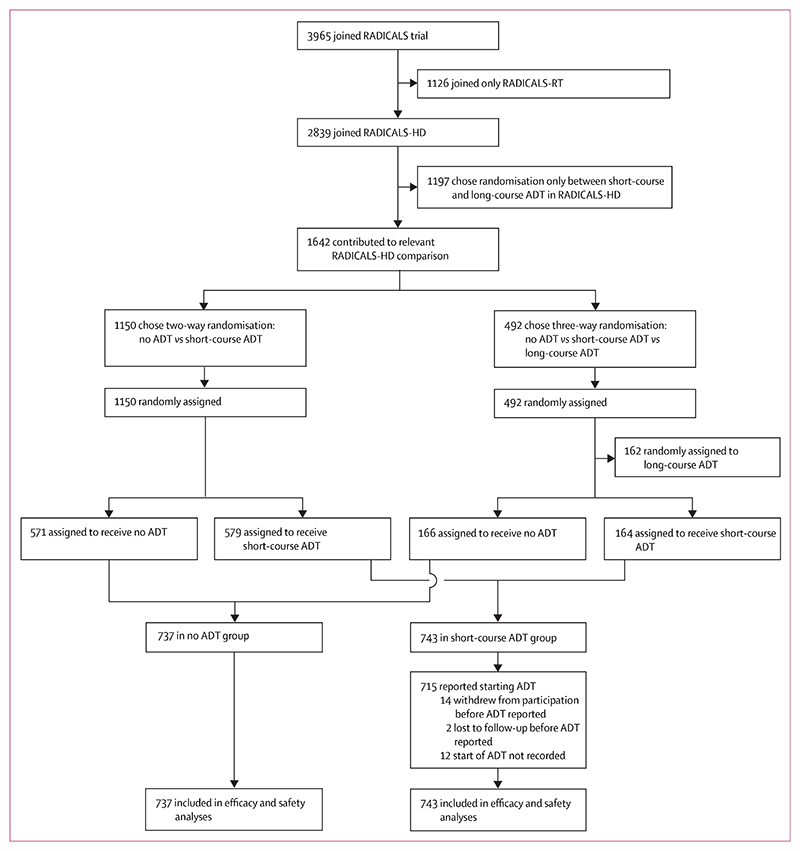
Trial profile ADT=androgen deprivation therapy.

Between Nov 22, 2007, and June 29, 2015, 1480 patients were randomly assigned to receive no ADT (n=737) or short-course (6 months) ADT (n=743) in addition to postoperative radiotherapy at 121 trial-accredited centres in Canada, Denmark, Ireland, and the UK. (figure 1). Of those, 1150 joined who had been randomly assigned between only these two groups and 330 joined who had been allocated to one of these two groups as part of the three-way randomisation that also included long-course ADT.

The median age of participants was 66 years (IQR 61–69); 1267 (86%) of 1478 had a Gleason score of 7 or higher and 240 (16%) of 1471 had stage T3B disease or higher (table 1). Data on race and ethnicity were not collected. Radiotherapy was initiated in the early salvage setting in 1057 (71%) of 1480 participants. The planned radiotherapy schedule was 66 Gy in 33 fractions for 1021 (69%) of 1480 participants, and the radiotherapy target was the prostate bed only for 1392 (94%) of 1480.

Follow-up at sites for the trial ended on Dec 31, 2021: 1209 of 1480 patients were still in follow-up at that date, 190 were known to have died, and 81 had stopped their participation or been lost to follow-up. Median follow-up was 9·0 years (IQR 7·1–10·1). Among those still in active follow-up at the end of the trial, minimum follow-up was 4·5 years. The database was locked on May 27, 2022.

In the short-course ADT group, median time from randomisation to starting hormone treatment was 7 days (IQR 1–14). 28 patients were allocated to short-course ADT with no record of starting treatment, 14 of whom formally withdrew their participation from the trial. A formal date for stopping hormone treatment was not reported for 80 of the patients allocated to short-course ADT.

**Figure 2:**
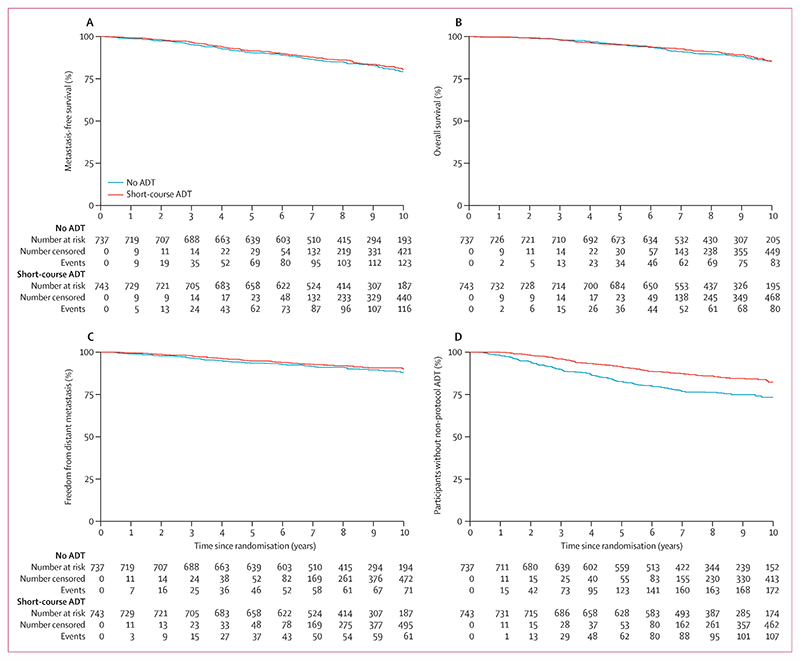
Primary and secondary outcome measures (A) Metastasis-free survival. (B) Overall survival. (C) Freedom from distant metastasis. (D) Time to non-protocol ADT. Risk tables present the number of participants who, at each timepoint, remain at risk, have been censored, or have had an event. All timepoints add up to the total number of patients. ADT=androgen deprivation therapy.

**Figure 3:**
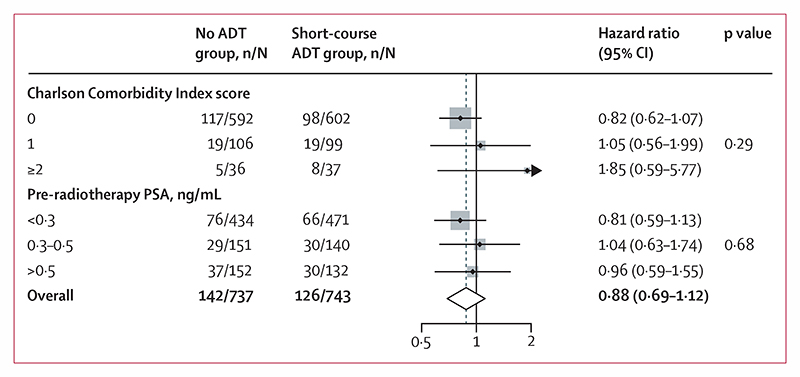
Pre-planned subgroup analyses Weighting is by sample size. Age did not contribute to the Charlson Comorbidity Index score. ADT=androgen deprivation therapy. PSA=prostate-specific antigen.

Metastasis-free survival events were reported for 268 patients, including 142 in the no ADT group and 126 in the short-course ADT group; 78 patients (44 no ADT and 34 short-course ADT) developed metastases but were still alive at the end of the trial, 59 patients (31 no ADT and 28 short-course ADT) had metastases followed by death, and 131 (67 no ADT and 64 short-course ADT) died without having metastases reported. There was no evidence that metastasis-free survival was improved in patients allocated to short-course ADT compared with those allocated to no ADT (HR 0·886 [95% CI 0·688–1·140], p=0·35; table 2, figure 2). There was no evidence of non-proportional hazards in the treatment effect. 10-year metastasis-free survival was 79·2% (95% CI 75·4–82·5) in the no ADT group and 80·4% (76·6–83·6) in the short-course ADT group. The metastasis-free survival treatment effect did not differ meaningfully in either of the two prespecified subgroup analyses, pre-radiotherapy PSA level (interaction p=0·68) and Charlson Comorbidity Index score (interaction p=0·29; figure 3), nor by any of the exploratory randomisation stratification factors (appendix p 2).

The secondary outcome measures in which there was clear evidence for a benefit of short-course ADT compared with no ADT were clinical progression-free survival and time to non-protocol (salvage) ADT. For clinical progression-free survival, the HR was 0·544 (95% CI 0·433–0·684; log-rank p<0·0001), although with some evidence of non-proportional hazards. For time to non-protocol ADT, the HR was 0·543 (0·422–0·699; log-rank p<0·0001) but with clear evidence of non-proportional hazards, so this is better summarised as improved restricted mean event-free time from 8·45 years (95% CI 8·24–8·66) to 9·17 years (9·01–9·32) with short-course ADT. We found no evidence for a benefit of short-course ADT for overall survival, nor for freedom from distant metastasis, which only included deaths from prostate. In a competing-risks regression model with non-prostate cancer death as the competing risk, the sub-HR was 0·814 (95% CI 0·586–1·130; p=0·22). Causes of death are presented in the appendix (p 3).

During follow-up, Radiation Therapy Oncology Group scale toxicity of grade 3 or higher was reported for 121 (17%) of 737 participants in the no ADT group and 100 (14%) of 743 in the short-course ADT group (p=0·15; table 3). The most commonly reported toxicities of grade 3 or higher were urethral stricture and haematuria. Of the serious adverse events reported, 18 (11 in the no ADT group and seven in the short-course ADT group) were reported as serious adverse reactions and two (both in the short-course ADT group) were reported as suspected unexpected serious adverse reactions (appendix p 4). Five deaths were reported as serious adverse events; none were considered as definitely, probably, or possibly related to trial treatment on clinical review.

## Discussion

In this randomised controlled trial, the addition of 6 months of ADT to postoperative prostate bed radiotherapy did not improve metastasis-free survival, but it did delay the time to salvage ADT. These data should inform clinical decision making; 6 months of ADT upfront, at the time of postoperative radiotherapy, improved the 10-year freedom from salvage ADT from 73·3% (95% CI 69·5–76·7) to 82·3% (78·7–85·3). However, given that it had no meaningful impact on metastasis-free survival, short-course ADT is unlikely to improve overall survival in this setting.

Two other randomised controlled trials have tested the use of short-course ADT with postoperative radiotherapy to the prostate bed. GETUG-AFU 16 was a randomised controlled trial for patients with PSA progression after radical prostatectomy with a primary outcome measure named as progression-free survival.^2^ However, that outcome measure could have been termed biochemical progression-free survival, because the definition of progression included PSA progression. 743 patients were randomly assigned to prostate bed radiotherapy with or without two doses of goserelin 10·8 mg given 3 months apart. In an updated analysis, at a median follow-up of 9·3 years,^2^ 10-year progression-free survival was 49% for radiotherapy alone and 64% for radiotherapy plus short-course ADT (HR 0·54 [95% CI 0·43–0·68]; p<0·0001). Metastasis-free survival was not a prespecified outcome measure of the trial, but 10-year metastasis-free survival was 69% for radiotherapy alone and 75% for radiotherapy plus short-course ADT (HR 0·73 [0·54–0·98]; p=0·039). No difference in overall survival was reported, with 85% and 86% alive at 10 years, respectively (HR 0·93 [0·63–1·39]; p=0·73). Similarly, RTOG 0534 was a trial that enrolled people with PSA progression after radical prostatectomy.^3^ Its primary outcome measure was freedom from progression and, like GETUG-AFU 16, that definition of progression included increasing PSA as well as clinical progression or death, with PSA driving the event count. 1194 men were randomly assigned to prostate bed radiotherapy with or without 4–6 months of ADT. With a median follow-up of 8·2 years,^3^ 5-year freedom from progression was 71% for radiotherapy alone and 81% for radiotherapy plus short-course ADT (HR 0·64 [0·50–0·82]; p<0·0001). There was no clear evidence of a benefit for adding short-course ADT in terms of distant metastases (0·78 [0·52–1·17]; p=0·083), prostate cancer death (0·79 [0·45–1·38]; p=0·17), or overall survival (0·89 [0·60–1·31]; p=0·245). The results from GETUG-AFU 16 and RTOG 0534 will be combined with those from RADICALS-HD in the DADSPORT meta-analysis (registered on PROSPERO, CRD42022325769), which will provide evidence concerning the effect of short-course ADT on long-term clinical outcomes in this setting. We note that all three trials are consistent with the possibility of an improvement in metastasis-free survival. The DADSPORT meta-analysis will also study the optimum duration of ADT, including the short-course versus long-course ADT comparison from RADICALS-HD.^13^

The design of RADICALS-HD differed from GETUG-AFU 16 and RTOG 0534 with regard to the primary outcome measure. Our view was that short-course ADT would be expected to delay PSA progression after postoperative radiotherapy, and yet such a positive finding should not be sufficient to justify its use. Rather, the case for or against short-course ADT should rest on more clinically meaningful outcome measures. When RADICALS-HD was first designed, the primary outcome measure was disease-specific survival. This was later amended to metastasis-free survival based on the work of ICECaP.^14^ The full details of this change and the broader history of RADICALS are presented elsewhere.^15^ The findings from ICECaP^14^ demonstrated metastasis-free survival to be a useful intermediate outcome measure in trials of non-metastatic prostate cancer: unless a substantial effect is seen on metastasis-free survival, it is unlikely that the intervention will improve overall survival. Therefore, given that these three trials have not demonstrated a substantial impact on metastasis-free survival for the addition of short-course ADT, we believe that prostate bed radiotherapy alone remains a standard treatment option.

The most promising findings of the RTOG 0534 trial were in the group that received not only the addition of short-course ADT but also pelvic nodal irradiation. In comparison with prostate bed radiotherapy alone, treatment to the prostate bed and the pelvic lymph nodes plus short-course ADT gave the best point estimate for metastasis-free survival benefit (HR 0·79 [95% CI 0·57–1·08], p=0·042) but this did not reach their threshold for statistical significance (p<0·0125). In current clinical practice, we believe that there is a choice to be made between prostate bed radiotherapy alone versus the combination of radiotherapy to the prostate bed and pelvic lymph node plus ADT. Patients prioritising safety might opt for prostate bed radiotherapy alone. Patients willing to accept the increased risk of adverse effects in the hope of greater efficacy might opt for radiotherapy to the prostate bed and the pelvic lymph nodes plus ADT.

The adverse effects of ADT are already well characterised. Therefore, this trial reduced the data collection burden on sites by focusing only on possible radiotherapy side-effects using the Radiation Therapy Oncology Group scale. We believe the focused collection of data and the use of follow-up schedules that reflected normal clinical practice facilitated the conduct of RADICALS-HD. This allowed the trial to recruit sufficient participants to be powered on long-term, clinically meaningful outcome measures.

RADICALS-HD has several limitations. First, the large majority of patients received radiotherapy to the prostate bed alone, whereas results from RTOG 0534 have shown some support for radiotherapy to the pelvic nodes in addition to the prostate bed.^3^ It remains unclear whether short-course ADT might be beneficial in patients receiving pelvic nodal radiotherapy. Second, RADICALS-HD included patients receiving radiotherapy both in the salvage setting, like in GETUG-AFU 16 and RTOG 0534, and also in the adjuvant setting. Based on the previous results of RADICALS-RT,^15^ and the ARTISTIC meta-analysis,^18^ postoperative radiotherapy is now typically given in the salvage, rather than the adjuvant, setting. Given that some patients receiving adjuvant radiotherapy would have been cured by surgery alone, a lower event rate would be expected than in the salvage radiotherapy setting. In this analysis, we found no evidence of a differential effect for short-course ADT according to the timing of radiotherapy. The DADSPORT meta-analysis (CRD42022325769) of these three trials will include a sensitivity analysis, restricted to those treated in the salvage setting. Third, the good prognosis of this patient group makes clinical trials difficult. Although the trial opened more than 15 years ago, and the comparison accrued around 1500 patients, there are too few events for RADICALS-HD to reliably report on any effect on overall survival or cancer-specific survival. However, given the lack of improvement in metastasis-free survival, it is very unlikely that any benefit will emerge in terms of overall or cause-specific survival. Fourth, although we did a prespecified subgroup analysis by baseline comorbidity score, only a small proportion of the patients had a Charlson Comorbidity Index score of 2 or higher. We cannot be confident that the trial results are generalisable to patients with substantial comorbidity. The trial was in active follow-up during the COVID-19 pandemic from 2020 onwards. Recruitment had been completed many years previously so neither accrual nor the allocated treatment would have been affected. There is no good reason to think follow-up would have been impacted separately by allocated treatment group during the pandemic. Fifth, data were not collected on ethnicity and race, so we cannot comment on how well the participants reflect the underlying population, especially in light of well known differences in prevalence;^19^ the trial would not have been powered to look reliably for consistency of effect by ethnicity and race.

The trial was run when bone scan and CT scan were regarded as the standard imaging modalities. In recent years, new imaging techniques, such as prostate-specific membrane antigen (PSMA) PET, have become available, and it is interesting to consider the impact that this might have had on interpreting the trial results. PSMA PET is more sensitive than conventional imaging techniques and this would tend to bring forward the detection of metastatic disease during follow-up and increase the event rate. However, patients with detectable metastatic disease at baseline were excluded from the trial, and so the use of PSMA PET would lead to a lower-risk trial population and a lower event rate. We do not envisage that the use of new imaging techniques would have had a differential impact between the two trial groups.

In conclusion, RADICALS-HD found that the addition of 6 months of ADT to postoperative prostate bed radiotherapy did not improve metastasis-free survival, although it did delay the time to salvage ADT. This reduction in salvage ADT could be considered insufficient to justify 6 months of ADT at the time of radiotherapy. However, it is unclear whether the reduction in salvage ADT means that some patients will avoid the need for salvage ADT entirely, or whether the start of salvage ADT is merely being delayed. In our view, these findings are not sufficient to routinely recommend the use of short-course ADT with postoperative radiotherapy.

## Supplementary Material

Appendix

## Figures and Tables

**Table 1:** Participant characteristics and pre-randomisation planned treatment

	No ADT group (n=737)	Short-course ADT (n=743)	All (n=1480)
Age, years	66 (61–69)	66 (61–69)	66 (61–69)
PSA at randomisation, ng/mL	0·22 (0·12–0·40)	0·20 (0·10–0·40)	0·21 (0·11–0·40)
<0·3	434 (59%)	471 (63%)	905 (61%)
0·3 to <0·5	151 (20%)	140 (19%)	291 (20%)
≥0·5	152 (21%)	132 (18%)	284 (19%)
Gleason score			
<7	102 (14%)	109 (15%)	211 (14%)
3 + 4	345 (47%)	359 (48%)	704 (48%)
4 + 3	205 (28%)	189 (25%)	394 (27%)
>7	83 (11%)	86 (12%)	169 (11%)
Missing	2	0	2
T stage			
1 or 2	298 (41%)	305 (41%)	603 (41%)
3a	325 (44%)	303 (41%)	628 (43%)
3b or 3c	109 (15%)	122 (17%)	231 (16%)
4	3 (<1%)	6 (1%)	9 (1%)
Missing	2	7	9
Lymph node involvement			
Node negative	418 (57%)	394 (53%)	812 (55%)
Node positive	25 (3%)	25 (3%)	50 (3%)
No dissection	292 (40%)	319 (43%)	611 (41%)
Missing	2	5	7
Positive margins			
Absent	285 (39%)	271 (36%)	556 (38%)
Present	452 (61%)	472 (64%)	924 (62%)
CAPRA-S score			
Low (0–2)	111 (15%)	110 (15%)	221 (15%)
Intermediate (3–5)	386 (53%)	379 (51%)	765 (52%)
High (≥6)	235 (32%)	249 (34%)	484 (33%)
Missing	5	5	10
Charlson Comorbidity Index score			
0	592 (81%)	602 (82%)	1194 (81%)
1	106 (14%)	99 (13%)	205 (14%)
≥2	36 (5%)	37 (5%)	73 (5%)
Missing	3	5	8
Country			
UK	584 (79%)	578 (78%)	1162 (79%)
Canada	145 (20%)	156 (21%)	301 (20%)
Denmark	6 (1%)	8 (1%)	14 (1%)
Ireland	2 (<1%)	1 (<1%)	3 (<1%)
Timing of radiotherapy			
Adjuvant	208 (28%)	215 (29%)	423 (29%)
Early salvage	529 (72%)	528 (71%)	1057 (71%)
Planned radiotherapy schedule			
52·5 Gy in 20 fractions	215 (29%)	222 (30%)	437 (30%)
66·0 Gy in 33 fractions	510 (69%)	511 (69%)	1021 (69%)
Other	12 (2%)	10 (1%)	22 (1%)
Planned radiotherapy target			
Prostate bed	700 (95%)	692 (93%)	1392 (94%)
Prostate bed and lymph nodes	37 (5%)	51 (7%)	88 (6%)
Planned hormone therapy			
Gonadotropin-releasing hormone analogue	613 (83%)	624 (84%)	1237 (84%)
Bicalutamide	124 (17%)	119 (16%)	243 (16%)

Data are median (IQR), n (%), or n. Percentages were calculated using the number of participants with available data as the denominator. ADT=androgen deprivation therapy. PSA=prostate-specific antigen. CAPRA-S=Cancer of the Prostate Risk Assessment Post-Surgical.

**Table 2:** Primary and secondary outcome measures

	No ADT group (n=737)	Short-course ADT group (n=743)	HR (95% CI)*	Log-rank p value*	Proportional hazards p value†
**Metastasis-free survival**					
Events	142	126	0·886 (0·688–1·140)	0·35	0·71
Metastases first	75	62	··	··	··
Prostate cancer death first	4	3	··	··	··
Death from other causes first	63	61	··	··	··
RMST (95% CI)‡	9·06 (8·90–9·22)	9·18 (9·03–9·33)	··	··	··
10-year metastasis-free survival (95% CI)	79·2% (75·4–82·5)	80·4% (76·6–83·6)	··	··	··
**Overall survival**					
Events	98	92	0·882 (0·651–1·194)	0·42	0·34
RMST (95% CI)‡	9·45 (9·33–9·57)	9·47 (9·35–9·59)	··	··	··
10-year overall survival (95% CI)	85·6% (82·2–88·4)	85·3% (81·7–88·3)	··	··	··
**Freedom from distant metastasis**					
Events	79	65	0·816 (0·579–1·150)	0·24	0·60
RMST (95% CI)‡	9·39 (9·26–9·53)	9·53 (9·41–9·65)	··	··	··
10-year freedom from distant metastasis (95% CI)	88·1% (85·0–90·6)	89·9% (87·0–92·2)	··	··	··
**Clinical progression-free survival**					
Events	210	131	0·544 (0·433–0·684)	<0·0001	0·071
RMST (95% CI)‡	8·16 (7·94–8·38)	8·96 (8·79–9·14)	··	··	··
10-year clinical progression-free survival (95% CI)	68·3% (64·3–71·9)	79·4% (75·8–82·6)	··	··	··
**Time to non-protocol ADT**					
Events	176	109	0·543 (0·422–0·699)	<0·0001	0·0041
RMST (95% CI)‡	8·45 (8·24–8·66)	9·17 (9·01–9·32)	··	··	··
10-year freedom from non-protocol ADT (95% CI)	73·3% (69·5–76·7)	82·3% (78·7–85·3)	··	··	··

ADT=androgen deprivation therapy. HR=hazard ratio. RMST=restricted mean survival time.

* Adjusted for randomisation stratification factors.

† Grambsch–Therneau test of non-proportional hazards.

‡ Restricted to 10 years.

**Table 3:** Maximum toxicity grade reported on Radiation Therapy Oncology Group scales

	No ADT group (n=737)				Short-course ADT group (n=743)			p value
	Grade 1–2	Grade 3	Grade 4		Grade 1–2	Grade 3	Grade 4	
Any toxicity	407 (55%)	114 (15%)	7 (1%)		442 (59%)	90 (12%)	10 (1%)	0·17
Diarrhoea	307 (42%)	12 (2%)	1 (<1%)		322 (43%)	13 (2%)	0	0·67
Proctitis	200 (27%)	23 (3%)	0		248 (33%)	20 (3%)	0	0·028
Cystitis	199 (27%)	15 (2%)	2 (<1%)		214 (29%)	17 (2%)	2 (<1%)	0·84
Haematuria	141 (19%)	51 (7%)	2 (<1%)		155 (21%)	28 (4%)	2 (<1%)	0·058
Urethral stricture	74 (10%)	49 (7%)	3 (<1%)		74 (10%)	51 (7%)	7 (1%)	0·65

Data are presented as n (%). Data were missing for six participants in the no ADT group and nine participants in the short-course ADT group. No grade 5 events were recorded. ADT=androgen deprivation therapy.

## Data Availability

The RADICALS trial data are held at the MRC Clinical Trials Unit at UCL, which encourages optimal use of data by using a controlled access approach to data sharing. Requests for data can be made at any time and can be initiated by email to mrcctu.datareleaserequest@ucl.ac.uk or via our website. There is a formal application process, whereby the request will undergo review by the trial team, as well as independent researchers, to ensure that the proposed research is both ethical and has a strong scientific rationale. Data will not be released if this would compromise the ongoing research. The specific data and associated documents to be shared will be dependent on the nature of the individual request and this will be documented in a formal data sharing agreement.

## Research in context

### Evidence before this study

In patients receiving postoperative radiotherapy after radical prostatectomy, the role of short-course androgen deprivation therapy (ADT) is uncertain. Two randomised controlled trials, GETUG-AFU 16 and RTOG 0534, have tested the addition of short-course ADT to salvage radiotherapy to the prostate bed. Both found evidence in favour of short-course ADT in terms of freedom from prostate-specific antigen (PSA) progression. However, ADT will inevitably delay PSA progression, and that alone should not be sufficient to change practice. The impact of short-course ADT on longer-term, clinically meaningful outcome measures is not known.

### Added value of this study

To our knowledge, RADICALS-HD is the first randomised trial of short-course ADT in men having postoperative radiotherapy that has used a long-term, clinically meaningful, primary outcome measure of metastasis-free survival. The results do not suggest that the impact of short-course ADT on PSA progression will translate into a clinically meaningful benefit in metastasis-free survival or overall survival.

### Implications of all the available evidence

These findings are not sufficient to routinely recommend the use of short-course ADT with postoperative radiotherapy for people with prostate cancer. A meta-analysis of all the randomised trials addressing this issue is warranted and will provide the best evidence to guide practice.

## References

[1] Kishan AU, Sun Y, Hartman H (2022). Androgen deprivation therapy use and duration with definitive radiotherapy for localised prostate cancer: an individual patient data meta-analysis. Lancet Oncol.

[2] Carrie C, Magné N, Burban-Provost P (2019). Short-term androgen deprivation therapy combined with radiotherapy as salvage treatment after radical prostatectomy for prostate cancer (GETUG-AFU 16): a 112-month follow-up of a phase 3, randomised trial. Lancet Oncol.

[3] Pollack A, Karrison TG, Balogh AG (2022). The addition of androgen deprivation therapy and pelvic lymph node treatment to prostate bed salvage radiotherapy (NRG Oncology/RTOG 0534 SPPORT): an international, multicentre, randomised phase 3 trial. Lancet.

[4] Taylor LG, Canfield SE, Du XL (2009). Review of major adverse effects of androgen-deprivation therapy in men with prostate cancer. Cancer.

[5] Lee LW, Clarke NW, Ramani VA, Cowan RA, Wylie JP, Logue JP (2005). Adjuvant and salvage treatment after radical prostatectomy: current practice in the UK. Prostate Cancer Prostatic Dis.

[6] Morris SL, Parker C, Huddart R, Horwich A, Dearnaley D (2004). Current opinion on adjuvant and salvage treatment after radical prostatectomy. Clin Oncol (R Coll Radiol).

[7] Parker C, Castro E, Fizazi K (2020). Prostate cancer: ESMO Clinical Practice Guidelines for diagnosis, treatment and follow-up. Ann Oncol.

[8] Spratt DE (2018). Evidence-based risk stratification to guide hormone therapy use with salvage radiation therapy for prostate cancer. Int J Radiat Oncol Biol Phys.

[9] Cox JD, Stetz J, Pajak TF (1995). Toxicity criteria of the Radiation Therapy Oncology Group (RTOG) and the European Organization for Research and Treatment of Cancer (EORTC). Int J Radiat Oncol Biol Phys.

[10] Parker CC, Parmar MKB, Sydes MR, Cook A (2022). RADICALS trial statistical analysis plan.

[11] Parker CC, Petersen PM, Cook AD (2024). Timing of radiotherapy (RT) after radical prostatectomy (RP): long-term outcomes in the RADICALS-RT trial [NCT00541047]. Ann Oncol.

[12] Parker CC, Clarke NW, Cook AD (2020). Timing of radiotherapy after radical prostatectomy (RADICALS-RT): a randomised, controlled phase 3 trial. Lancet.

[13] Parker CC, Kynaston H, Cook AD (2024). Duration of androgen deprivation therapy with postoperative radiotherapy for prostate cancer: a comparison of long-course versus short-course androgen deprivation therapy in the RADICALS-HD randomised trial. Lancet.

[14] Xie W, Regan MM, Buyse M (2017). Metastasis-free survival is a strong surrogate of overall survival in localized prostate cancer. J Clin Oncol.

[15] Parker CC, Clarke NW, Catton C (2022). RADICALS-HD: reflections before the results are known. Clin Oncol (R Coll Radiol).

[16] Morris TP, Jarvis CI, Cragg W, Phillips PPJ, Choodari-Oskooei B, Sydes MR (2019). Proposals on Kaplan–Meier plots in medical research and a survey of stakeholder views: KMunicate. BMJ Open.

[17] Charlson ME, Pompei P, Ales KL, MacKenzie CR (1987). A new method of classifying prognostic comorbidity in longitudinal studies: development and validation. J Chronic Dis.

[18] Vale CL, Fisher D, Kneebone A (2020). Adjuvant or early salvage radiotherapy for the treatment of localised and locally advanced prostate cancer: a prospectively planned systematic review and meta-analysis of aggregate data. Lancet.

[19] James ND, Tannock I, N’Dow J (2024). The *Lancet* Commission on prostate cancer: planning for the surge in cases. Lancet.

