# Supplementary material for: Adding 6 months of androgen deprivation therapy to postoperative radiotherapy for prostate cancer: a comparison of short-course versus no androgen deprivation therapy in the RADICALS-HD randomised controlled trial
Source: Lancet. Author manuscript; Available in PMC 2024 Aug 14. (PMC7616360; doi:10.1016/S0140-6736(24)00548-8)
Supplement: Appendix [file EMS197882-supplement-Appendix.pdf]

THE LANCET

Supplementary appendix

This appendix formed part of the original submission and has been peer reviewed. We post it as supplied by the authors.

Supplement to: Parker CC, Clarke NW, Cook AD, et al. Adding 6 months of androgen deprivation therapy to postoperative radiotherapy for prostate cancer: a comparison of short-course versus no androgen deprivation therapy in the RADICALS-HD randomised controlled trial. *Lancet* 2024; published online May 16. https://doi.org/10.1016/S0140-6736(24)00548-8.

WebAppendix

Adding 6 months of androgen deprivation therapy to postoperative radiotherapy for prostate cancer: a comparison of short-course versus no androgen deprivation therapy in the RADICALS-HD randomised controlled trial
(doi: 10.1016/S0140-6736(24)00548-8)

Table of Contents

Page	Item
2	Supplementary Table 1: Subgroup analysis of randomisation stratification factors
3	Supplementary Table 2: Deaths, by cause
4	Supplementary Table 3: Serious adverse events, causality and resolution
5	Sites contributing to comparison
8	Note on ethical approval
Separate	Full site of trials unit staff, site investigators and site staff
Separate	Protocol

Supplementary Table 1: Subgroup analysis of randomisation stratification factors

	No ADT (n=737)		Short-course ADT (n=743)		Treatment effect		
	N	Events	N	Events	HR	(95% CI)	p‡
**Gleason score**							
<7	102	17	109	15	0.90	(0.45, 1.80)	0.928
7	552	99	548	87	0.89	(0.67, 1.18)	
>7	83	26	86	24	0.79	(0.45, 1.38)	
**Positive margins**							
Absent	285	49	271	52	1.13	(0.77, 1.67)	0.116
Present	452	93	472	74	0.76	(0.56, 1.03)	
**Planned RT schedule**							
52.5Gy, 20 fractions	215	46	222	42	0.93	(0.61, 1.41)	0.933
66Gy, 33 fractions	510	93	511	82	0.87	(0.64, 1.16)	
Other	12	3	10	2	0.76	(0.13, 4.59)	
**Timing of RT**							
Adjuvant	208	32	215	34	1.07	(0.66, 1.74)	0.325
Salvage	529	110	528	92	0.82	(0.62, 1.08)	
**Planned ADT**							
GnRH agonist	613	123	624	115	0.92	(0.72, 1.19)	0.224
Bicalutamide	124	19	119	11	0.56	(0.27, 1.18)	

‡ Chi-square test for heterogeneity

Key

ADT = Androgen deprivation therapy

GnRH = Gonadotrophin-releasing hormone

Gy = Gray

HR = Hazard Ratio

P = p-value

RT = Radiotherapy

Supplementary Table 2: Deaths, by cause

	**No ADT** (Deaths=98)		**Short-course ADT** (Deaths=92)	
	N	(%)	N	(%)
Prostate cancer	29	(31%)	18	(20%)
Other cancer	32	(34%)	31	(35%)
Cardio-vascular	8	(9%)	8	(9%)
Other	25	(27%)	32	(36%)
Unknown	*4*		*3*	

Supplementary Table 3: Serious adverse events, causality and resolution

Item	No ADT (n=737)		Short-course ADT (n=743)		
	N	(%)	N	(%)	p
**Total SAE reported ‡**	**33**		**27**		
**Total SAR reported ‡**	**11**		**7**		
**Total SUSAR reported ‡**	**0**		**2**		
**Relatedness to treatment after central review**					
Definitely	4	(13%)	0	(0%)	0.182
Probably	6	(20%)	5	(20%)	
Possibly	5	(17%)	6	(24%)	
Unlikely	1	(3%)	4	(16%)	
Unrelated	14	(47%)	10	(40%)	
Missing	*3*	*(n/a)*	*2*	*(n/a)*	
**Resolution**					
Resolved	21	(64%)	18	(69%)	
Resolved with sequelae	6	(18%)	5	(19%)	
Ongoing	3	(9%)	1	(4%)	
Fatal Ψ	3	(9%)	2	(8%)	
Missing	*0*	*(n/a)*	*1*	*(n/a)*	

‡ No participants had more than one SAE / SAR / SUSAR reported
Ψ None of the fatal SAEs were reported as related to treatment

Key

ADT = Androgen deprivation therapy

SAE = Serious Adverse Event

Sites contributing to comparison

Note: Full list of sites participating across RADICALS and their nominated staff follow after the table.

97 = London, UK: Mount Vernon Hospital [Middlesex]
61 = Sherbrooke, Canada: CIUSSS de l'Estrie - Centre hospitalier universitaire de Sherbrooke
58 = London, UK: Guy's Hospital
54 = Toronto, Canada: Princess Margaret Hospital
50 = Winnipeg, Canada: CancerCare Manitoba
45 = Canterbury, UK: Kent and Canterbury Hospital
43 = Stockport, UK: Stepping Hill Hospital
39 = Manchester, UK: Christie Hospital
38 = Birmingham, UK: Queen Elizabeth Hospital
36 = Northampton, UK: Northampton General Hospital
30 = Coventry, UK: University Hospital Coventry and Warwickshire
28 = Crewe, UK: Leighton Hospital
28 = Leeds, UK: St James University Hospital
26 = Sutton, UK: Royal Marsden Hospital
25 = Romford, UK: Queen's Hospital
25 = Wakefield, UK: Pinderfields Hospital
24 = Guildford, UK: Royal Surrey County Hospital
22 = London, UK: Royal Marsden Hospital
21 = Montreal, Canada: CHUM - Hopital Notre-Dame
21 = Ottawa, Canada: Ottawa Health Research Institute
21 = Plymouth, UK: Derriford Hospital
20 = Maidstone, UK: Maidstone Hospital
20 = Salford, UK: Salford Royal Hospital
20 = Wolverhampton, UK: New Cross Hospital
19 = Bradford, UK: Bradford Royal Infirmary
19 = Toronto, Canada: Odette Cancer Centre - Sunnybrook Health Sciences Centre
17 = Cardiff, UK: University Hospital of Wales
16 = Exeter, UK: Royal Devon and Exeter Hospital
16 = Wirral, UK: Clatterbridge Centre for Oncology
15 = Brighton, UK: Royal Sussex County Hospital
15 = London, UK: Charing Cross Hospital
15 = Norwich, UK: Norfolk and Norwich University Hospital
15 = Southend, UK: Southend University Hospital
15 = Trois-Riveries, Canada: Centre Hospitalier Regional de Trois-Rivieries
14 = Blackburn, UK: Royal Blackburn Hospital
14 = London, UK: Croydon University Hospital
14 = Preston, UK: Royal Preston Hospital
13 = Inverness, UK: Raigmore Hospital
12 = Bath, UK: Royal United Hospital
12 = Lincoln, UK: Lincoln County Hospital
12 = Shrewsbury, UK: Royal Shrewsbury Hospital
11 = Bodelwyddan, UK: Glan Clwyd Hospital
11 = Colchester, UK: Essex County Hospital
11 = Quebec City, Canada: Hotel-Dieu de Quebec
11 = Scunthorpe, UK: Scunthorpe General Hospital
11 = Taunton, UK: Musgrove Park Hospital
11 = Truro, UK: Royal Cornwall Hospital
10 = Aarhus, Denmark: Aarhus Kommunehospital
10 = Derby, UK: Royal Derby Hospital
10 = Stoke, UK: Royal Stoke University Hospital
9 = Birmingham, UK: Birmingham Heartlands Hospital
9 = Bournemouth, UK: Royal Bournemouth Hospital
9 = Bristol, UK: Bristol Haematology & Oncology Centre
9 = Dorchester, UK: Dorset County Hospital

9 = Nottingham, UK: Nottingham University Hospitals
9 = Poole, UK: Poole Hospital
9 = Southampton, UK: Southampton General Hospital
8 = Edinburgh, UK: Western General Hospital
8 = London, Canada: London Regional Cancer Program [ON]
8 = Weston-super-Mare, UK: Weston General Hospital
7 = Ayr, UK: Ayr Hospital
7 = Oldham, UK: Royal Oldham Hospital
7 = York, UK: York District Hospital
6 = Aberdeen, UK: Aberdeen Royal Infirmary
6 = Hereford, UK: Hereford County Hospital
6 = Kingston, Canada: Cancer Centre of Southeastern Ontario at Kingston General Hospital
6 = Kitchener, Canada: Grand River Regional Cancer Centre, Ontario
6 = London, UK: Whipps Cross University Hospital
6 = London, UK: North Middlesex Hospital
6 = Oxford, UK: Churchill Hospital
6 = Vancouver, Canada: BC Cancer Agency-Fraser Valley
5 = Cheltenham, UK: Cheltenham General Hospital
5 = Kelowna, Canada: BC Cancer Centre for Southern Interior
5 = London, UK: St Marys Hospital
5 = London, UK: St Bartholomews Hospital
5 = Vancouver, Canada: Vancouver Cancer Centre
4 = Belfast, UK: Belfast City Hospital
4 = Glasgow, UK: Beatson West of Scotland Cancer Centre
4 = Harlow, UK: Princess Alexandra Hospital
4 = Ipswich, UK: Ipswich Hospital
4 = Middlesbrough, UK: James Cook University Hospital
4 = Newcastle, UK: Freeman Hospital
4 = Redditch, UK: Alexandra Hospital
4 = St Johns, Canada: Dr. H. Bliss Murphy Cancer Centre [NL]
4 = Victoria, Canada: Vancouver Island Cancer Centre
3 = Airedale, UK: Airedale General Hospital
3 = Barnstaple, UK: North Devon District Hospital
3 = Berkshire, UK: Royal Berkshire Hospital
3 = Bristol, UK: Southmead Hospital
3 = Cambridge, UK: Addenbrooke's Hospital
3 = Galway, Ireland: University College Hospital
3 = Peterborough, UK: Peterborough District Hospital
3 = Swindon, UK: Great Western Hospital
3 = Torbay, UK: Torbay District General Hospital
3 = Warwick, UK: Warwick Hospital
2 = Aalborg, Denmark: Aalborg University Hospital
2 = Barrie, Canada: Royal Victoria Hospital
2 = Basingstoke, UK: Basingstoke and North Hampshire Hospital
2 = Eastbourne, UK: Eastbourne District General Hospital
2 = Glasgow, UK: The New Victoria ACH
2 = Herlev, Denmark: Amtssygehuset i Herlev - Herlev University Hospital
2 = Larbert, UK: Forth Valley Royal Hospital
2 = Newport, UK: Royal Gwent Hospital
2 = Sutton Coldfield, UK: Good Hope Hospital
2 = Worthing, UK: Worthing Hospital
1 = Aylesbury, UK: Stoke Mandeville Hospital
1 = Chelmsford, UK: Broomfield Hospital
1 = Chester, UK: Countess of Chester Hospital
1 = Halifax, Canada: Queen Elizabeth II Health Sciences Centre/ Nova Scotia Cancer Centre
1 = London, UK: St Georges Hospital
1 = Manchester, UK: Withington Hospital
1 = Montreal, Canada: Hospital Maisonneuve-Rosemont
1 = Oshawa, Canada: Lakeridge Health
1 = Sutton-in-Ashfield, UK: King's Mill Hospital

1 = Swansea, UK: Singleton Hospital
1 = Worcester, UK: Worcestershire Royal Hospital
1 = Yeovil, UK: Yeovil

Note on ethical approval

Each participating country took a different approach to ethics committee approval. This was the standard approach advised for that nation at that time.

Country	Approach
UK	National approval: Health Research Authority's Research Ethics Committees
Ireland	(one centre): Galway Clinical Ethics Committee
Canada	Hospital by hospital approval, including: Ontario Cancer Research Ethics Board
Denmark	One hospital for all: De videnskabsetiske komiteer for Region Hovedstaden (The Regional Committee for the Capital Region)

RADICALS OVERSIGHT COMMITTEES, STAFF AND COLLABORATORS

Version: 11-Jul-2023

CONTENTS

NOTES 2
TRIAL MANAGEMENT GROUP 3
INDEPENDENT DATA MONITORING COMMITTEE 4
TRIAL STEERING COMMITTEE 5
TRIALS UNIT STAFF 6
INVESTIGATORS AND COLLABORATORS: SITE STAFF 8
PARTICIPANTS 88

NOTES

Investigators and site staff are those who have formally appeared at any time on a site’s Delegation Logs.

CTU staff are those who have worke8d on or contributed to the trial any time from the outset until the date of this report.

The independent members of the Independent Data Monitoring Committee and Trial Oversight Committee play an important role in the conduct of the trial.

Industry collaborators are a subset of the people who have worked, on relevant sections, with the trial staff to ensure the trial runs efficiently.

TRIAL MANAGEMENT GROUP
(Listing only member external to the trials unit or senior staff at the trials unit)

Area	Status	Member	Geography	Role
Clinical/Surgical	Current	Charles Catton	Toronto, Canada	Country Lead
~~	~~~	Noel Clarke	Manchester, UK	
~~	~~~	William Cross	York, UK	Surgical Champion
~~	~~~	Howard Kynaston	Cardiff, UK	
~~	~~~	John Logue	Stockport, UK	RT advisor
~~	~~~	Peter Meidahl	Copenhagen, Denmark	Country Lead
~~	~~~	Chris Parker	Sutton, UK	Chief Investigator
~~	~~~	Heather Payne	London, UK	RT advisor
~~	~~~	Rajendra Persad	Bristol, UK	Surgical Champion
~~	~~~	Fred Saad	Montreal, Canada	
~~	Previous	Kilian Mellon	Leicester, UK	
~~	~~~	Chris Morash	Canada, UK	
~~	~~~	Garrett Durkan	Galway, Ireland	Country Lead
Nurse specialist	Current	Lorna Bower		
~~	Previous	Jane Gosling		
~~	~~~	Angela Lee		
PPI	Current	Peter Neville		
~~	~~~	Alan Stirling		
~~	Previous	Peter Barton		
~~	~~~	Ian Jamieson		
~~	~~~	Mike Sawkins		
~~	~~~	Jim Stansfeld		
Senior trials units	Current	Adrian Cook	MRC CTU at UCL	
~~	~~~	Max Parmar	MRC CTU at UCL	
~~	~~~	Wendy Parulekar	CCTG	
~~	~~~	Cheryl Pugh	MRC CTU at UCL	
~~	~~~	Matthew Sydes	MRC CTU at UCL	
~~	Previous	Claire Amos	MRC CTU at UCL	
~~	~~~	Silvia Forcat	MRC CTU at UCL	
~~	~~~	Barbara Uscinska	MRC CTU at UCL	

Key: PPI = Patient and public involvement
RT = Radiotherapy

Note: The full list of trials units staff is detailed below in a subsequent section.

INDEPENDENT DATA MONITORING COMMITTEE
(All members independent)

Member	Status	Role
Murray Brunt	Current	Chair 2
Mary Gospodorawicz	Current	Member
Jim Montie	~~~	~~~
Cindy Billingham	~~~	~~~
David Landau	Previous	Chair 1

TRIAL STEERING COMMITTEE
(Listing only independent members)

Member	Status	Role	
John Chester	Current	Chair 4	MRC CTU Cancer umbrella TSC
Emma Crosbie	~~~	~~~	MRC CTU Cancer umbrella TSC
Lucy Kilburn	~~~	~~~	MRC CTU Cancer umbrella TSC
Richard Stephens	~~~	~~~	MRC CTU Cancer umbrella TSC
Jeremy Whelan	Previous	Chair 2	MRC CTU Cancer umbrella TSC
Anne Thomas	~~~	Chair 3	MRC CTU Cancer umbrella TSC
Anne Russell	~~~	Member	MRC CTU Cancer umbrella TSC
Judith Bliss	~~~	~~~	MRC CTU Cancer umbrella TSC
Hisham Mehanna	~~~	~~~	MRC CTU Cancer umbrella TSC
David Guthrie	Previous	Chair 1	MRC CTU Cancer Urology TSC
Stan Dische	~~~	Member	MRC CTU Cancer Urology TSC
Michael Jewett	~~~	~~~	MRC CTU Cancer Urology TSC
John Scholefield	~~~	~~~	MRC CTU Cancer Urology TSC

TRIALS UNIT STAFF

Country	Trials Unit	Role	Status	Name
UK	MRC CTU at UCL	Statistician	Current	Adrian Cook
~~	~~~	~~~	Previous	Chris Brawley
~~	~~~	~~~	~~~	Andrew Embleton
~~	~~~	~~~	~~~	Gordana Jovic
~~	~~~	~~~	~~~	Rachel Morgan
~~	~~~	~~~	~~~	Max Parmar
~~	~~~	~~~	~~~	Matthew Sydes
~~	~~~	Clinical Project Manager	Current	Cheryl Pugh
~~	~~~	~~~	Previous	Claire Amos
~~	~~~	~~~	~~~	Silvia Forcat
~~	~~~	~~~	~~~	Barbara Uscinska
~~	~~~	Trial Manager	Previous	Cindy Goldstein
~~	~~~	~~~	~~~	Claire Murphy
~~	~~~	~~~	~~~	Dipa Noor
~~	~~~	~~~	~~~	Holly Pickering
~~	~~~	~~~	~~~	Carol Roach
~~	~~~	~~~	~~~	Hannah Sims
~~	~~~	Data Manager	Current	Christos Maniatis
~~	~~~	~~~	Previous	Katherine Beaney
~~	~~~	~~~	~~~	Katharine Bellenger
~~	~~~	~~~	~~~	Jenna Grabey
~~	~~~	~~~	~~~	Anna Herasimtschuk
~~	~~~	~~~	~~~	Paul Patterson
~~	~~~	~~~	~~~	Helena Ribeiro
~~	~~~	~~~	~~~	Fatimah Seray-Wurie
~~	~~~	~~~	~~~	Ben Spittle
~~	~~~	~~~	~~~	Lilian Tsang
~~	~~~	DMS PM	Current	Christina Chung
~~	~~~	~~~	Previous	Nancy Tappenden
~~	~~~	Data Scientist	Current	Fatima Mohamed
~~	~~~	~~~	Previous	Dominic Hague
~~	~~~	~~~	~~~	Lindsey Masters
~~	~~~	Programmer	Current	Preetha Shaji
~~	~~~	~~~	Previous	Zaheer Islam
~~	~~~	~~~	~~~	Mary Rauchenberger
Canada	CCTG	Physician Coordinator	Current	Wendy Parulekar
~~	~~~	~~~	Previous	Ralph Meyer
~~	~~~	~~~	~~~	Harriet Richardson
~~	~~~	Study Coordinator	Current	Cathy Davidson
~~	~~~	~~~	Previous	Conor Dellar
~~	~~~	~~~	~~~	Kate Whelan
~~	~~~	Clinical Trials Associate	Current	Karen Richardson
~~	~~~	~~~	Previous	Sue Casey

TRIALS UNIT STAFF

Country	Trials Unit	Role	Status	Name
~~	~~~	Research Associate	Previous	Mandy Fletcher
~~	~~~	~~~	~~~	Karen Murphy
~~	~~~	Oracle	Previous	Teddy Brown

INVESTIGATORS AND COLLABORATORS: SITE STAFF

Staff on site delegation logs

Country	Site	Names	Role
Canada	Abbotsford, BC: Fraser Valley Cancer Centre	Anand Karvat	Site PI
~~	~~~	Arthur Cheung	Clinical/Surgical
~~	~~~	Winkle Kwan	Clinical/Surgical
~~	~~~	Cheryl Carrasco	Principal CRA
~~	~~~	Gloria Garrioch	Principal CRA
~~	~~~	Janice Jong	Principal CRA
~~	~~~	Jen Darker	Principal CRA
~~	~~~	Cheryl Carrasco	Ethics CRA
~~	~~~	Gloria Garrioch	Ethics CRA
~~	~~~	Janice Jong	Ethics CRA
~~	~~~	Jen Darker	Ethics CRA
~~	~~~	Arlissa Johnson	Additional CRA
~~	~~~	Cathy Jackson	Additional CRA
~~	~~~	Cheryl Carrasco	Additional CRA
~~	~~~	Debbie Jepson	Additional CRA
~~	~~~	Donna Mitchell	Additional CRA
~~	~~~	Gloria Garrioch	Additional CRA
~~	~~~	Janice Jong	Additional CRA
~~	~~~	Michael W Braun	Additional CRA
~~	~~~	Monica Fourt	Additional CRA
~~	~~~	Noelle Baird	Additional CRA
~~	~~~	Rosanne Serpanchy	Additional CRA
~~	~~~	Sandeep Sandhu	Additional CRA
~~	~~~	Sue McIndoe	Additional CRA
~~	~~~	Helen Wu	Pharmacist
Canada	Barrie, ON: Royal Victoria Regional Health Centre	Christiaan Stevens	Site PI
~~	~~~	Adam Gladwish	Clinical/Surgical
~~	~~~	Frederick Yoon	Clinical/Surgical
~~	~~~	Gerard Morton	Clinical/Surgical
~~	~~~	Jason Yu	Clinical/Surgical
~~	~~~	Kimberley MarshGray	Principal CRA
~~	~~~	Michele Harris	Principal CRA
~~	~~~	Christine DiMarco	Ethics CRA
~~	~~~	Kayla Gerrity	Ethics CRA

INVESTIGATORS AND COLLABORATORS: SITE STAFF

Staff on site delegation logs

Country	Site	Names	Role
~~	~~~	Caitlin Pascoe	Additional CRA
~~	~~~	Cara Murphy	Additional CRA
~~	~~~	Ian Ding	Additional CRA
~~	~~~	Kayla Gerrity	Additional CRA
~~	~~~	Melanie Crawford	Additional CRA
~~	~~~	Michele Harris	Additional CRA
~~	~~~	Patricia MacIsaac	Additional CRA
~~	~~~	Rachelle Beausoleil	Additional CRA
~~	~~~	Sujata Pokhrel	Additional CRA
Canada	Brandon, MB: Western Manitoba Cancer Centre	Bashir Bashir	Site PI
~~	~~~	William Hunter	Site PI
~~	~~~	Arbind Dubey	Clinical/Surgical
~~	~~~	Bashir Bashir	Clinical/Surgical
~~	~~~	Gokulan Sivananthan	Clinical/Surgical
~~	~~~	William Hunter	Clinical/Surgical
~~	~~~	Joelle DuMontier	Principal CRA
~~	~~~	Leanne Anderson	Principal CRA
~~	~~~	Joelle DuMontier	Ethics CRA
~~	~~~	Leanne Anderson	Ethics CRA
~~	~~~	Leanne Anderson	Additional CRA
Canada	CAHN	Maroie Barkati	Site PI
~~	~~~	Carole Lambert	Clinical/Surgical
~~	~~~	Cynthia Menard	Clinical/Surgical
~~	~~~	Daniel Taussky	Clinical/Surgical
~~	~~~	Fred Saad	Clinical/Surgical
~~	~~~	Guila Delouya	Clinical/Surgical
~~	~~~	JeanPaul Bahary	Clinical/Surgical
~~	~~~	MarieClaude Beauchemin	Clinical/Surgical
~~	~~~	Pierre Rousseau	Clinical/Surgical
~~	~~~	Sophie Lavertu	Clinical/Surgical
~~	~~~	Adriana Carbonaro	Principal CRA
~~	~~~	Alexandra Frazzi	Principal CRA
~~	~~~	Chantal Lafleur	Principal CRA
~~	~~~	Siew Siew Pan	Principal CRA

INVESTIGATORS AND COLLABORATORS: SITE STAFF

Staff on site delegation logs

Country	Site	Names	Role
~~	~~~	Diane Trudel	Ethics CRA
~~	~~~	Mom Phat	Ethics CRA
~~	~~~	Chantal Lafleur	Additional CRA
~~	~~~	Mom Phat	Additional CRA
~~	~~~	Nidale ElSokhn	Additional CRA
~~	~~~	Silvine Benth	Additional CRA
Canada	Edmonton, AB: Cross Cancer Institute	Brita Danielson	Site PI
~~	~~~	Albert Murtha	Clinical/Surgical
~~	~~~	Don Yee	Clinical/Surgical
~~	~~~	Jim Rose	Clinical/Surgical
~~	~~~	John Oliver Amanie	Clinical/Surgical
~~	~~~	Matthew B Parliament	Clinical/Surgical
~~	~~~	Nadeem Pervez	Clinical/Surgical
~~	~~~	Nawaid Usmani	Clinical/Surgical
~~	~~~	Robert Pearcey	Clinical/Surgical
~~	~~~	Samir Patel	Clinical/Surgical
~~	~~~	Karen Tracey	Principal CRA
~~	~~~	Nirmal Joshi	Principal CRA
~~	~~~	Karen Tracey	Ethics CRA
~~	~~~	Nirmal Joshi	Ethics CRA
~~	~~~	Wanda Churchill	Ethics CRA
~~	~~~	Beverly Larson	Additional CRA
~~	~~~	Candra Williams	Additional CRA
~~	~~~	Carlie Smith	Additional CRA
~~	~~~	Debbie Mallett	Additional CRA
~~	~~~	Leni Santiago Garcia	Additional CRA
~~	~~~	Monika Lang	Additional CRA
~~	~~~	Nirmal Joshi	Additional CRA
~~	~~~	Wanda Churchill	Additional CRA
~~	~~~	Shelley Parker	Pharmacist
~~	~~~	Carol Borynec	Other
~~	~~~	Caroline Shewchuk	Other
~~	~~~	Colin Gramlich	Other
~~	~~~	Margaret Batz	Other

INVESTIGATORS AND COLLABORATORS: SITE STAFF

Staff on site delegation logs

Country	Site	Names	Role
~~	~~~	Sylvia McCrudden	Other
Canada	Halifax, NS: QEII Health Sciences Centre	David Bowes	Site PI
~~	~~~	Helmut Hollenhorst	Site PI
~~	~~~	Abdulla AlRashdan	Clinical/Surgical
~~	~~~	David Bowes	Clinical/Surgical
~~	~~~	Derek Wilke	Clinical/Surgical
~~	~~~	Nikhilesh Patil	Clinical/Surgical
~~	~~~	Paul K Joseph	Clinical/Surgical
~~	~~~	Robert DH Rutledge	Clinical/Surgical
~~	~~~	Erin Little	Principal CRA
~~	~~~	Heather Beaton	Principal CRA
~~	~~~	Kendra Dill	Principal CRA
~~	~~~	Emily Moffatt	Ethics CRA
~~	~~~	Jennifer MacVicar	Ethics CRA
~~	~~~	Joan Nieforth	Ethics CRA
~~	~~~	Robin Simpson	Ethics CRA
~~	~~~	Stevie Dugas	Ethics CRA
~~	~~~	Alison Avery	Additional CRA
~~	~~~	Angela MacDonald	Additional CRA
~~	~~~	Brittany Bond	Additional CRA
~~	~~~	Camryn Salyzyn	Additional CRA
~~	~~~	Connie Zinck	Additional CRA
~~	~~~	Donna Sutherland	Additional CRA
~~	~~~	Heather Walker	Additional CRA
~~	~~~	Jillian McCracken	Additional CRA
~~	~~~	Kara Bursey	Additional CRA
~~	~~~	Kathy MacIsaac	Additional CRA
~~	~~~	Kelsey van der Rijt	Additional CRA
~~	~~~	Lane Carvery	Additional CRA
~~	~~~	Lorrie Yunace	Additional CRA
~~	~~~	Lynn Hubley	Additional CRA
~~	~~~	Susan Burbridge	Additional CRA
~~	~~~	Tanya Yeo	Additional CRA
~~	~~~	Tina Rose	Additional CRA

INVESTIGATORS AND COLLABORATORS: SITE STAFF
Staff on site delegation logs

Country	Site	Names	Role
~~	~~~	Victoria Roberts	Additional CRA
~~	~~~	Claudia Harding	Pharmacist
~~	~~~	June Hodder	Pharmacy technician
~~	~~~	Kim BrucePayne	Other
Canada	Hamilton, ON: Juravinski Cancer Centre at Hamilton Health Sciences	Thomas B Corbett	Site PI
~~	~~~	Himu Lukka	Clinical/Surgical
~~	~~~	Ian S Dayes	Clinical/Surgical
~~	~~~	Jason W T Wong	Clinical/Surgical
~~	~~~	Malti Behn Patel	Clinical/Surgical
~~	~~~	Susan O Gudelis	Clinical/Surgical
~~	~~~	Thedoros Tsakirdis	Clinical/Surgical
~~	~~~	Diane DeRosa	Principal CRA
~~	~~~	Elaine Hill	Ethics CRA
~~	~~~	Yvonne Kinrade	Ethics CRA
~~	~~~	Anne Laughlin	Additional CRA
~~	~~~	Barbara Makepeace	Additional CRA
~~	~~~	Bianca Bier	Additional CRA
~~	~~~	Carmela Oliverio	Additional CRA
~~	~~~	Catherine Bucci	Additional CRA
~~	~~~	Diane DeRosa	Additional CRA
~~	~~~	Linda Nielsen	Additional CRA
~~	~~~	Lynn Marshall	Additional CRA
~~	~~~	Robin Eady	Additional CRA
Canada	Kelowna, BC: Cancer Centre for the Southern Interior	David W Petrik	Site PI
~~	~~~	Mohamed Manji	Site PI
~~	~~~	Bernard Lee	Clinical/Surgical
~~	~~~	David Kim	Clinical/Surgical
~~	~~~	David W Petrik	Clinical/Surgical
~~	~~~	Francois Bachand	Clinical/Surgical
~~	~~~	Juanita Crook	Clinical/Surgical
~~	~~~	Melanie Reed	Clinical/Surgical
~~	~~~	Mohamed Manji	Clinical/Surgical
~~	~~~	Ross Halperin	Clinical/Surgical
~~	~~~	Allana Scarfo	Principal CRA

INVESTIGATORS AND COLLABORATORS: SITE STAFF
Staff on site delegation logs

Country	Site	Names	Role
~~	~~~	Cherie Bates	Principal CRA
~~	~~~	Jeaneen Rudolph	Principal CRA
~~	~~~	Jessica Stojcic	Principal CRA
~~	~~~	Allana Scarfo	Ethics CRA
~~	~~~	Cherie Bates	Ethics CRA
~~	~~~	Jeaneen Rudolph	Ethics CRA
~~	~~~	Jessica Stojcic	Ethics CRA
~~	~~~	Aldyn Overend	Additional CRA
~~	~~~	Allana Scarfo	Additional CRA
~~	~~~	Brianna Creelman	Additional CRA
~~	~~~	Carol Winn	Additional CRA
~~	~~~	Cherie Bates	Additional CRA
~~	~~~	Danielle GeorgeChayka	Additional CRA
~~	~~~	Debbie Opitz	Additional CRA
~~	~~~	Flo Kronstal	Additional CRA
~~	~~~	Janet Yanchuk	Additional CRA
~~	~~~	Jeaneen Rudolph	Additional CRA
~~	~~~	Karen Wilkie	Additional CRA
~~	~~~	Kirsten Allen	Additional CRA
~~	~~~	Marie McClelland	Additional CRA
~~	~~~	Michaella Watson	Additional CRA
~~	~~~	Nancy Hartt	Additional CRA
~~	~~~	Shannon Cotts	Additional CRA
~~	~~~	Layton Carefoot	Pharmacist
~~	~~~	TuongVan Kim	Pharmacist
~~	~~~	Jill A Hayes	Other
~~	~~~	Tanya Lew	Other
Canada	Kingston, ON: Kingston Health Sciences Centre	Khaled O Zaza	Site PI
~~	~~~	Aamer Mahmud	Clinical/Surgical
~~	~~~	Carey Shenfield	Clinical/Surgical
~~	~~~	Maria Kalyvas	Clinical/Surgical
~~	~~~	Michael Brundage	Clinical/Surgical
~~	~~~	Vikaash Kumar	Clinical/Surgical
~~	~~~	Amber Burley	Principal CRA

INVESTIGATORS AND COLLABORATORS: SITE STAFF

Staff on site delegation logs

Country	Site	Names	Role
~~	~~~	Carrie Lindsay	Principal CRA
~~	~~~	Fred Zeltser	Principal CRA
~~	~~~	Jessica Ferguson	Principal CRA
~~	~~~	Amber Burley	Ethics CRA
~~	~~~	Angel Zhou	Ethics CRA
~~	~~~	Ashlee Young	Ethics CRA
~~	~~~	Carrie Lindsay	Ethics CRA
~~	~~~	Chloe Sutton	Ethics CRA
~~	~~~	Cindy Carlson	Ethics CRA
~~	~~~	Deborah Leach	Ethics CRA
~~	~~~	Denise KirbyBello	Ethics CRA
~~	~~~	Heather Doucette	Ethics CRA
~~	~~~	James Medd	Ethics CRA
~~	~~~	Jennifer Pritchard	Ethics CRA
~~	~~~	Jessica Ferguson	Ethics CRA
~~	~~~	Karen MacVicar	Ethics CRA
~~	~~~	Kristina Carmichael	Ethics CRA
~~	~~~	Noureen Hassan	Ethics CRA
~~	~~~	Rhonda Harpell	Ethics CRA
~~	~~~	Stephanie Raposo	Ethics CRA
~~	~~~	Tracy Vermette	Ethics CRA
~~	~~~	Abby Murano	Additional CRA
~~	~~~	Angel Zhou	Additional CRA
~~	~~~	Ashlee Young	Additional CRA
~~	~~~	Carrie Hartman	Additional CRA
~~	~~~	Carrie Lindsay	Additional CRA
~~	~~~	Chloe Sutton	Additional CRA
~~	~~~	Christine Maize	Additional CRA
~~	~~~	Craig Spencer	Additional CRA
~~	~~~	Hala ElKerdawy	Additional CRA
~~	~~~	Heather Doucette	Additional CRA
~~	~~~	Jackie Edwards	Additional CRA
~~	~~~	James Medd	Additional CRA
~~	~~~	Jennifer Pritchard	Additional CRA

INVESTIGATORS AND COLLABORATORS: SITE STAFF

Staff on site delegation logs

Country	Site	Names	Role
~~	~~~	Jessica Ferguson	Additional CRA
~~	~~~	Kristina Carmichael	Additional CRA
~~	~~~	Nancy Paul	Additional CRA
~~	~~~	Noureen Hassan	Additional CRA
~~	~~~	Stephanie Raposo	Additional CRA
~~	~~~	Stephanie Willing	Additional CRA
~~	~~~	Virginia Tennant	Additional CRA
~~	~~~	Yvonne Moelker	Additional CRA
~~	~~~	Marleen RossSmith	Pharmacist
~~	~~~	Ashley Ross	Other
~~	~~~	Mike Brander	Other
~~	~~~	Scott Bonner	Other
Canada	Kitchener, ON: Grand River Regional Cancer Centre at Grand River Hosp	Joda Kuk	Site PI
~~	~~~	Darindra Gopaul	Clinical/Surgical
~~	~~~	Jochen Knackstedt	Clinical/Surgical
~~	~~~	Ramana Rachakonda	Clinical/Surgical
~~	~~~	Ronald Hamilton	Clinical/Surgical
~~	~~~	Elyse Wellhauser	Principal CRA
~~	~~~	Kelly Walker	Principal CRA
~~	~~~	Kristin Krokoszynski	Principal CRA
~~	~~~	Susan Janke	Principal CRA
~~	~~~	Carol Ballantyne	Ethics CRA
~~	~~~	Anissa Mumin	Additional CRA
~~	~~~	Atif Siddiqui	Additional CRA
~~	~~~	Brenda S Shantz	Additional CRA
~~	~~~	Carla Girolametto	Additional CRA
~~	~~~	Carol Ballantyne	Additional CRA
~~	~~~	Debra Hendel	Additional CRA
~~	~~~	Elyse Wellhauser	Additional CRA
~~	~~~	Jennifer Gearing	Additional CRA
~~	~~~	Kelly Walker	Additional CRA
~~	~~~	Kristin Krokoszynski	Additional CRA
~~	~~~	MaryBeth Morrison	Additional CRA
~~	~~~	Sheeba Thallury	Additional CRA

INVESTIGATORS AND COLLABORATORS: SITE STAFF
Staff on site delegation logs

Country	Site	Names	Role
~~	~~~	Stephanie Nemirov	Additional CRA
Canada	London, ON: London Regional Cancer Program	George B Rodrigues	Site PI
~~	~~~	Belal Ahmad	Clinical/Surgical
~~	~~~	David DSouza	Clinical/Surgical
~~	~~~	Glenn Bauman	Clinical/Surgical
~~	~~~	Michael I Lock	Clinical/Surgical
~~	~~~	Tracy Sexton	Clinical/Surgical
~~	~~~	Varagur M Venkatesan	Clinical/Surgical
~~	~~~	Kes Sebborn	Principal CRA
~~	~~~	Christina Gurzanski	Ethics CRA
~~	~~~	Laura Bailey	Ethics CRA
~~	~~~	Mary Beth Husson	Ethics CRA
~~	~~~	Patricia Moore	Ethics CRA
~~	~~~	Shannon Kenny	Ethics CRA
~~	~~~	Albert Gratton	Additional CRA
~~	~~~	Craig Johnson	Additional CRA
~~	~~~	Danylle Corkery	Additional CRA
~~	~~~	Darlene tenHaaf	Additional CRA
~~	~~~	Elenor DiLullo	Additional CRA
~~	~~~	Heather Mayer	Additional CRA
~~	~~~	Jennifer A Hare	Additional CRA
~~	~~~	Kate Loughlin	Additional CRA
~~	~~~	Lois Eichenberger	Additional CRA
~~	~~~	Mary Beth Husson	Additional CRA
~~	~~~	Patricia Moore	Additional CRA
~~	~~~	Peggy Francis	Additional CRA
~~	~~~	Stephen Mardell	Additional CRA
~~	~~~	Susan Grant	Additional CRA
~~	~~~	Lori Sax	Pharmacist
~~	~~~	Mary Jane Camara	Other
Canada	Montreal, QC: CIUSSS de l'Est-de-l'Île de Montréal Hôpital Maisonneuve-Rosemont	Peter Vavassis	Site PI
~~	~~~	Alma Sylvestre	Clinical/Surgical
~~	~~~	Benoit Laliberte	Clinical/Surgical
~~	~~~	Celine Lemaire	Clinical/Surgical

INVESTIGATORS AND COLLABORATORS: SITE STAFF

Staff on site delegation logs

Country	Site	Names	Role
~~	~~~	David H A Nguyen	Clinical/Surgical
~~	~~~	Michael Yassa	Clinical/Surgical
~~	~~~	Nader Khaouam	Clinical/Surgical
~~	~~~	Josee AbiSaad	Principal CRA
~~	~~~	Linda RoyHuneault	Principal CRA
~~	~~~	Veronique Tran	Principal CRA
~~	~~~	Josee AbiSaad	Ethics CRA
~~	~~~	Linda RoyHuneault	Ethics CRA
~~	~~~	Veronique Tran	Ethics CRA
~~	~~~	AnneMarie Chatelain	Additional CRA
~~	~~~	Jacqueline Fortin	Additional CRA
~~	~~~	Linda RoyHuneault	Additional CRA
~~	~~~	Nicole Lachance	Additional CRA
~~	~~~	Stefanie Houle	Additional CRA
Canada	Montreal, QC: McGill University	Marie Duclos	Site PI
~~	~~~	George Shenouda	Clinical/Surgical
~~	~~~	Luis Souhami	Clinical/Surgical
~~	~~~	Sergio Luiz Faria	Clinical/Surgical
~~	~~~	MarieClaude Joncas	Principal CRA
~~	~~~	Penny Chipman	Principal CRA
~~	~~~	Rajesh Sharma	Principal CRA
~~	~~~	Brenda Lee	Ethics CRA
~~	~~~	Carolynna Olha	Ethics CRA
~~	~~~	Charlotte Corwin	Ethics CRA
~~	~~~	Linda Casey	Ethics CRA
~~	~~~	Stephanie Larocque	Ethics CRA
~~	~~~	Wayne Briand	Ethics CRA
~~	~~~	Jamila Adnaan	Additional CRA
~~	~~~	Mokhtar Ghoul	Additional CRA
~~	~~~	Zoe Koulouris	Pharmacist
~~	~~~	MarieFrance Robert	Other
Canada	Oshawa, ON: Lakeridge Health Oshawa	Wayne Koll	Site PI
~~	~~~	Audrey Li	Clinical/Surgical
~~	~~~	Fawaad Iqbal	Clinical/Surgical

INVESTIGATORS AND COLLABORATORS: SITE STAFF
Staff on site delegation logs

Country	Site	Names	Role
~~	~~~	Jimmy Mui	Clinical/Surgical
~~	~~~	Sten Myrehaug	Clinical/Surgical
~~	~~~	Ann Mueller	Principal CRA
~~	~~~	Helen Norton	Principal CRA
~~	~~~	Linda Klich	Principal CRA
~~	~~~	Virginia Albrecht	Principal CRA
~~	~~~	Edeliza Mendoza	Ethics CRA
~~	~~~	Nicole Stevens	Ethics CRA
~~	~~~	Aileen Manganaro	Additional CRA
~~	~~~	Angelina Singson	Additional CRA
~~	~~~	Ann Mueller	Additional CRA
~~	~~~	Ashane Somasiri	Additional CRA
~~	~~~	Dianne McKee	Additional CRA
~~	~~~	Helen Norton	Additional CRA
~~	~~~	Jane Froese	Additional CRA
~~	~~~	Linda Klich	Additional CRA
~~	~~~	Shannon Kift	Additional CRA
~~	~~~	Janet Slessor	Pharmacist
~~	~~~	Barbara MacGregor	Other
~~	~~~	Nancy Froude	Other
~~	~~~	Pamela Howitt	Other
Canada	Ottawa, ON: Ottawa Hosp Research Institute	Shawn Malone	Site PI
~~	~~~	Alain Haddad	Clinical/Surgical
~~	~~~	Choan E	Clinical/Surgical
~~	~~~	Gad A Perry	Clinical/Surgical
~~	~~~	JeanMarc Bourque	Clinical/Surgical
~~	~~~	Libni Eapen	Clinical/Surgical
~~	~~~	Robert M MacRae	Clinical/Surgical
~~	~~~	Scott Morgan	Clinical/Surgical
~~	~~~	Wayne S Kendal	Clinical/Surgical
~~	~~~	Caroline Proulx	Principal CRA
~~	~~~	Margaret MacGillivray	Principal CRA
~~	~~~	Scott Grimes	Principal CRA
~~	~~~	Stella Park	Principal CRA

INVESTIGATORS AND COLLABORATORS: SITE STAFF
Staff on site delegation logs

Country	Site	Names	Role
~~	~~~	Archana Tikoo	Ethics CRA
~~	~~~	Elina Iordanidi	Ethics CRA
~~	~~~	Femina Kanji	Ethics CRA
~~	~~~	Julie Nguyen	Ethics CRA
~~	~~~	Koralee Berghout	Ethics CRA
~~	~~~	Lisa Turriff	Ethics CRA
~~	~~~	Maria Sanchez	Ethics CRA
~~	~~~	MarieClaude Reeves	Ethics CRA
~~	~~~	Mary Spearman	Ethics CRA
~~	~~~	Matt Fish	Ethics CRA
~~	~~~	Nancy Eze	Ethics CRA
~~	~~~	Amy Carkner	Additional CRA
~~	~~~	Anna OBrien	Additional CRA
~~	~~~	Caroline Proulx	Additional CRA
~~	~~~	Joanne Roach	Additional CRA
~~	~~~	Kimberly Hicks	Additional CRA
~~	~~~	Lisa MacMullin	Additional CRA
~~	~~~	Margaret MacGillivray	Additional CRA
~~	~~~	Nancy Page	Additional CRA
~~	~~~	Natalie Wright	Additional CRA
~~	~~~	Patti Spencer	Additional CRA
~~	~~~	Scott Grimes	Additional CRA
~~	~~~	Stella Park	Additional CRA
Canada	Quebec City, QC: Hotel-Dieu de Quebec	Eric Vigneault	Site PI
~~	~~~	AndreGuy Martin	Clinical/Surgical
~~	~~~	JoseLuis Gomez	Clinical/Surgical
~~	~~~	Lara Hathout	Clinical/Surgical
~~	~~~	Leonello Cusan	Clinical/Surgical
~~	~~~	Luis Diaz de Bedoya	Clinical/Surgical
~~	~~~	William Foster	Clinical/Surgical
~~	~~~	Josee Allard	Principal CRA
~~	~~~	Isabelle Desrosiers	Ethics CRA
~~	~~~	Josee Allard	Ethics CRA
~~	~~~	Danielle Cossette	Additional CRA

INVESTIGATORS AND COLLABORATORS: SITE STAFF
Staff on site delegation logs

Country	Site	Names	Role
~~	~~~	Josee Allard	Additional CRA
~~	~~~	MarieAndree Lajoie	Additional CRA
~~	~~~	Nathalie Dufour	Additional CRA
~~	~~~	Sophie Pouliot	Additional CRA
Canada	Sherbrooke, QC: CIUSSS de l'Estrie - Centre hospitalier universitaire de Sherbrooke	Abdenour Nabid	Site PI
~~	~~~	Annie Ebacher	Clinical/Surgical
~~	~~~	Audrey TetreaultLaflamme	Clinical/Surgical
~~	~~~	Isabelle C Gauthier	Clinical/Surgical
~~	~~~	Michel Carmel	Clinical/Surgical
~~	~~~	Myriam Bouchard	Clinical/Surgical
~~	~~~	Rachel Bujold	Clinical/Surgical
~~	~~~	Robert Sabbagh	Clinical/Surgical
~~	~~~	Sophie Couture	Principal CRA
~~	~~~	Sophie Couture	Ethics CRA
~~	~~~	Alexandra Lamoureux	Additional CRA
~~	~~~	Cynthia Ladouceur	Additional CRA
~~	~~~	Diane Pearson	Additional CRA
~~	~~~	Genevieve Cote	Additional CRA
~~	~~~	Helene Dion	Additional CRA
~~	~~~	Julie Larouche	Additional CRA
~~	~~~	Tania Marquis	Additional CRA
Canada	Toronto, ON: Dr. H. Bliss Murphy Cancer Centre	Jinka R Sathya	Site PI
~~	~~~	John Thoms	Site PI
~~	~~~	Alia Norman	Clinical/Surgical
~~	~~~	Asim Kamran	Clinical/Surgical
~~	~~~	Craig Pochini	Clinical/Surgical
~~	~~~	Jonathan Greenland	Clinical/Surgical
~~	~~~	Allison Joy	Principal CRA
~~	~~~	Elysia Desai	Principal CRA
~~	~~~	Kelli Mitchell	Principal CRA
~~	~~~	Arifur Rahman	Ethics CRA
~~	~~~	Dawne Putt	Ethics CRA
~~	~~~	Erin Baker	Ethics CRA
~~	~~~	Lauren Rickert	Ethics CRA

INVESTIGATORS AND COLLABORATORS: SITE STAFF
Staff on site delegation logs

Country	Site	Names	Role
~~	~~~	Liz Fuller	Ethics CRA
~~	~~~	Matthew Nelder	Ethics CRA
~~	~~~	Stacy Whittle	Ethics CRA
~~	~~~	Allison Joy	Additional CRA
~~	~~~	Ashley Hoskins	Additional CRA
~~	~~~	Breanne Teasdale	Additional CRA
~~	~~~	Chrystal Whiteway	Additional CRA
~~	~~~	Denise Galway	Additional CRA
~~	~~~	Elysia Desai	Additional CRA
~~	~~~	Erin Pinnell	Additional CRA
~~	~~~	Gail House	Additional CRA
~~	~~~	Geoffrey Blackwood	Additional CRA
~~	~~~	Jamie ODea	Additional CRA
~~	~~~	Kelli Mitchell	Additional CRA
~~	~~~	Kimberley Manning	Additional CRA
~~	~~~	Liz Fuller	Additional CRA
~~	~~~	Lorilee Noel	Additional CRA
~~	~~~	Zeta Hannaford	Pharmacist
Canada	Toronto, ON: Odette Cancer Centre	Hans T Chung	Site PI
~~	~~~	Cyril Danjoux	Clinical/Surgical
~~	~~~	Danny Vesprini	Clinical/Surgical
~~	~~~	Douglas Andrew Loblaw	Clinical/Surgical
~~	~~~	Eric Chia Lin Tseng	Clinical/Surgical
~~	~~~	Ewa Szumacher	Clinical/Surgical
~~	~~~	Gerard Morton	Clinical/Surgical
~~	~~~	Laurence Klotz	Clinical/Surgical
~~	~~~	Patrick CF Cheung	Clinical/Surgical
~~	~~~	Robert K Nam	Clinical/Surgical
~~	~~~	Stanley Liu	Clinical/Surgical
~~	~~~	William Chu	Clinical/Surgical
~~	~~~	Anam Shahid	Principal CRA
~~	~~~	Andrea DeAbreu	Principal CRA
~~	~~~	Arlynne Marquez	Principal CRA
~~	~~~	Kristina Commisso	Principal CRA

INVESTIGATORS AND COLLABORATORS: SITE STAFF

Staff on site delegation logs

Country	Site	Names	Role
~~	~~~	Senny Chan	Principal CRA
~~	~~~	Vivette Escueta	Principal CRA
~~	~~~	Andrea DeAbreu	Ethics CRA
~~	~~~	Arlynne Marquez	Ethics CRA
~~	~~~	Kristina Commisso	Ethics CRA
~~	~~~	Senny Chan	Ethics CRA
~~	~~~	Zeeba Bhounr	Ethics CRA
~~	~~~	Anam Shahid	Additional CRA
~~	~~~	Andrea DeAbreu	Additional CRA
~~	~~~	Angela Commisso	Additional CRA
~~	~~~	Arlynne Marquez	Additional CRA
~~	~~~	Leila Malek	Additional CRA
~~	~~~	Zeeba Bhounr	Additional CRA
~~	~~~	Michael Leung	Pharmacist
Canada	Toronto, ON: University Health Network Princess Margaret Cancer Centre	Charles Catton	Site PI
~~	~~~	Alejandro Berlin	Clinical/Surgical
~~	~~~	Andrew J Bayley	Clinical/Surgical
~~	~~~	Cynthia Menard	Clinical/Surgical
~~	~~~	Joelle Helou	Clinical/Surgical
~~	~~~	Mary Gospodarowicz	Clinical/Surgical
~~	~~~	Michael Milosevic	Clinical/Surgical
~~	~~~	Nafisha Lalani	Clinical/Surgical
~~	~~~	Padraig Warde	Clinical/Surgical
~~	~~~	Peter Chung	Clinical/Surgical
~~	~~~	Robert Bristow	Clinical/Surgical
~~	~~~	Srinivas Raman	Clinical/Surgical
~~	~~~	Andrei Rotarescu	Principal CRA
~~	~~~	Evan Strom	Principal CRA
~~	~~~	Ida Lee	Principal CRA
~~	~~~	Mary Tannourji	Principal CRA
~~	~~~	Melissa Lem	Principal CRA
~~	~~~	Navneet Arora	Principal CRA
~~	~~~	Suzana Djuric	Principal CRA
~~	~~~	Andrei Rotarescu	Ethics CRA

INVESTIGATORS AND COLLABORATORS: SITE STAFF
Staff on site delegation logs

Country	Site	Names	Role
~~	~~~	Evan Strom	Ethics CRA
~~	~~~	Ida Lee	Ethics CRA
~~	~~~	Mary Tannourji	Ethics CRA
~~	~~~	Melissa Lem	Ethics CRA
~~	~~~	Navneet Arora	Ethics CRA
~~	~~~	Suzana Djuric	Ethics CRA
~~	~~~	Aleksandra Petrovska	Additional CRA
~~	~~~	Aleksandra Topalovich	Additional CRA
~~	~~~	Amadeus Chui	Additional CRA
~~	~~~	Andrei Rotarescu	Additional CRA
~~	~~~	Arundhati Shukla	Additional CRA
~~	~~~	Avi Petroff	Additional CRA
~~	~~~	Bernadeth Lao	Additional CRA
~~	~~~	Cynthia Torres	Additional CRA
~~	~~~	Daeria Lawson	Additional CRA
~~	~~~	Daniel Ang	Additional CRA
~~	~~~	Debbie Tsuji	Additional CRA
~~	~~~	Emily Gregorcic	Additional CRA
~~	~~~	Erin Velec	Additional CRA
~~	~~~	Evan Strom	Additional CRA
~~	~~~	Heidi Chan	Additional CRA
~~	~~~	Ida Lee	Additional CRA
~~	~~~	Jessy Abed	Additional CRA
~~	~~~	John Hsien	Additional CRA
~~	~~~	Judy Quintos	Additional CRA
~~	~~~	Karen Tse	Additional CRA
~~	~~~	Kathryn Sabate	Additional CRA
~~	~~~	Kris Wallace	Additional CRA
~~	~~~	Kyoko Tiessen	Additional CRA
~~	~~~	Maria Jose Gaitan Ruiz	Additional CRA
~~	~~~	Mary Tannourji	Additional CRA
~~	~~~	Nanthini Tharahan	Additional CRA
~~	~~~	Nicole Gumapac	Additional CRA
~~	~~~	Pat Merante	Additional CRA

INVESTIGATORS AND COLLABORATORS: SITE STAFF
Staff on site delegation logs

Country	Site	Names	Role
~~	~~~	Ramesh Mirmooji	Additional CRA
~~	~~~	Sandra Pineda	Additional CRA
~~	~~~	Sarah Degendorfer	Additional CRA
~~	~~~	Sarah Ramotar	Additional CRA
~~	~~~	Sarin Peres	Additional CRA
~~	~~~	Suzana Djuric	Additional CRA
~~	~~~	Vaishaali Thevarajah	Additional CRA
~~	~~~	Vickie Kong	Additional CRA
~~	~~~	Zaynab Muraj	Additional CRA
Canada	Trois-Rivieres, QC: Centre hospitalier regional de Trois-Rivieres	Francois Vincent	Site PI
~~	~~~	Benoit Lebrun	Clinical/Surgical
~~	~~~	Julie Harvey	Clinical/Surgical
~~	~~~	LindaSuzanne Vincent	Clinical/Surgical
~~	~~~	MarieEve Sicard	Clinical/Surgical
~~	~~~	Rafika Dahmane	Clinical/Surgical
~~	~~~	MarieEve Caron	Principal CRA
~~	~~~	MarieEve Caron	Ethics CRA
~~	~~~	Andreanne Thibeault	Additional CRA
~~	~~~	Julie Samson	Additional CRA
~~	~~~	Line Marineau	Additional CRA
~~	~~~	MarieEve Bisson	Additional CRA
~~	~~~	Vanessa Gagne	Additional CRA
~~	~~~	Julie Montour	Pharmacist
Canada	Vancouver, BC: Vancouver Cancer Centre	Abraham S Alexander	Site PI
~~	~~~	Jennifer Goulart	Site PI
~~	~~~	Abraham S Alexander	Clinical/Surgical
~~	~~~	Caroline Holloway	Clinical/Surgical
~~	~~~	Daniel Glick	Clinical/Surgical
~~	~~~	Emilie Priestley	Clinical/Surgical
~~	~~~	Howard H Pai	Clinical/Surgical
~~	~~~	Howard Joe	Clinical/Surgical
~~	~~~	Isabelle Vallieres	Clinical/Surgical
~~	~~~	Jacqueline Lam	Clinical/Surgical
~~	~~~	Jan Lim	Clinical/Surgical

INVESTIGATORS AND COLLABORATORS: SITE STAFF
Staff on site delegation logs

Country	Site	Names	Role
~~	~~~	Jennifer Goulart	Clinical/Surgical
~~	~~~	Joycelin Canavan	Clinical/Surgical
~~	~~~	Negin Shahid	Clinical/Surgical
~~	~~~	Nelson Leong	Clinical/Surgical
~~	~~~	Paul A Blood	Clinical/Surgical
~~	~~~	Emily White	Principal CRA
~~	~~~	Heather Lockyer	Principal CRA
~~	~~~	Michael A Miller	Principal CRA
~~	~~~	Sally Hodgson	Principal CRA
~~	~~~	Sarah Irons	Principal CRA
~~	~~~	Senz Hamilton	Principal CRA
~~	~~~	Emily White	Ethics CRA
~~	~~~	Heather Lockyer	Ethics CRA
~~	~~~	Michael A Miller	Ethics CRA
~~	~~~	Sally Hodgson	Ethics CRA
~~	~~~	Sarah Irons	Ethics CRA
~~	~~~	Senz Hamilton	Ethics CRA
~~	~~~	Alia Lomas	Additional CRA
~~	~~~	Cathy Lacey	Additional CRA
~~	~~~	Eleanor Holwerda	Additional CRA
~~	~~~	Heather Lockyer	Additional CRA
~~	~~~	Mia AnnaMaria Clements	Additional CRA
~~	~~~	Michael A Miller	Additional CRA
~~	~~~	Senz Hamilton	Additional CRA
Canada	Victoria, BC: Vancouver Island Cancer Centre	Charmaine KimSing	Site PI
~~	~~~	Alan I So	Clinical/Surgical
~~	~~~	Graeme Duncan	Clinical/Surgical
~~	~~~	Martin E Gleave	Clinical/Surgical
~~	~~~	Michael McKenzie	Clinical/Surgical
~~	~~~	Michael Peacock	Clinical/Surgical
~~	~~~	Mira Keyes	Clinical/Surgical
~~	~~~	S Larry Goldenberg	Clinical/Surgical
~~	~~~	Scott Tyldesley	Clinical/Surgical
~~	~~~	Sree Rodda	Clinical/Surgical

INVESTIGATORS AND COLLABORATORS: SITE STAFF

Staff on site delegation logs

Country	Site	Names	Role
~~	~~~	Thomas A Pickles	Clinical/Surgical
~~	~~~	Tina Wanting Zhang	Clinical/Surgical
~~	~~~	William James Morris	Clinical/Surgical
~~	~~~	Dana Matuszewski	Principal CRA
~~	~~~	Devon Poznanski	Principal CRA
~~	~~~	Lejla Gavranovic	Principal CRA
~~	~~~	Lorenz Yeung	Principal CRA
~~	~~~	Sandy Chang	Principal CRA
~~	~~~	Dana Matuszewski	Ethics CRA
~~	~~~	Devon Poznanski	Ethics CRA
~~	~~~	Lejla Gavranovic	Ethics CRA
~~	~~~	Lorenz Yeung	Ethics CRA
~~	~~~	Sandy Chang	Ethics CRA
~~	~~~	Sheena Sibug	Additional CRA
~~	~~~	Subashini Karunakaran	Additional CRA
~~	~~~	Lynne Nakashima	Pharmacist
Canada	Winnipeg, MB: CancerCare Manitoba	Aldrich Dixon Ong	Site PI
~~	~~~	Amitava D Chowdhury	Clinical/Surgical
~~	~~~	Arbind Dubey	Clinical/Surgical
~~	~~~	Atul Sharma	Clinical/Surgical
~~	~~~	Bashir Bashir	Clinical/Surgical
~~	~~~	Darrel E Drachenberg	Clinical/Surgical
~~	~~~	David Dawe	Clinical/Surgical
~~	~~~	Gokulan Sivananthan	Clinical/Surgical
~~	~~~	Hanbo Zhang	Clinical/Surgical
~~	~~~	Harvey Quon	Clinical/Surgical
~~	~~~	Jeffrey Graham	Clinical/Surgical
~~	~~~	Maged Nashed	Clinical/Surgical
~~	~~~	Rashmi Koul	Clinical/Surgical
~~	~~~	Shahida Ahmed	Clinical/Surgical
~~	~~~	William Hunter	Clinical/Surgical
~~	~~~	Amber DelisleCorps	Principal CRA
~~	~~~	Chinedu Ifeanyi	Principal CRA
~~	~~~	Christopher Schroeder	Principal CRA

INVESTIGATORS AND COLLABORATORS: SITE STAFF

Staff on site delegation logs

Country	Site	Names	Role
~~	~~~	Elizabeth Lylyk	Principal CRA
~~	~~~	Heidi Kampen	Principal CRA
~~	~~~	Maggie Putnam	Principal CRA
~~	~~~	Megan Ridler	Principal CRA
~~	~~~	Melissa Bridges	Principal CRA
~~	~~~	Sandy Yap	Principal CRA
~~	~~~	Swati Singla	Principal CRA
~~	~~~	Tianna Mohammed	Principal CRA
~~	~~~	Amber DelisleCorps	Ethics CRA
~~	~~~	Chinedu Ifeanyi	Ethics CRA
~~	~~~	Christopher Schroeder	Ethics CRA
~~	~~~	Christy Turner	Ethics CRA
~~	~~~	Darlene Zwarych	Ethics CRA
~~	~~~	DeepKumar Patel	Ethics CRA
~~	~~~	Elizabeth Lylyk	Ethics CRA
~~	~~~	Heidi Kampen	Ethics CRA
~~	~~~	Jennifer McNish	Ethics CRA
~~	~~~	Jill Jacinto	Ethics CRA
~~	~~~	JoAnn Weir	Ethics CRA
~~	~~~	Joyce Wei	Ethics CRA
~~	~~~	Laurel Johnston	Ethics CRA
~~	~~~	Leah Bergen	Ethics CRA
~~	~~~	Lovely Cadiz	Ethics CRA
~~	~~~	Maggie Putnam	Ethics CRA
~~	~~~	Megan Ridler	Ethics CRA
~~	~~~	Melissa Bridges	Ethics CRA
~~	~~~	Raquel Gamez	Ethics CRA
~~	~~~	Sandy Yap	Ethics CRA
~~	~~~	Swati Singla	Ethics CRA
~~	~~~	Theresa Moore	Ethics CRA
~~	~~~	Tianna Mohammed	Ethics CRA
~~	~~~	Allison Wood	Additional CRA
~~	~~~	Arpita Majumdar	Additional CRA
~~	~~~	Ashley Ouelette	Additional CRA

INVESTIGATORS AND COLLABORATORS: SITE STAFF

Staff on site delegation logs

Country	Site	Names	Role
~~	~~~	Barb Ammeter	Additional CRA
~~	~~~	Caitlin Kitkowski	Additional CRA
~~	~~~	Cristina Francisco	Additional CRA
~~	~~~	Debbie Last	Additional CRA
~~	~~~	Gina Garrett	Additional CRA
~~	~~~	Heather Davies	Additional CRA
~~	~~~	Heather Long	Additional CRA
~~	~~~	Heidi Kampen	Additional CRA
~~	~~~	Jacqueline Chahine	Additional CRA
~~	~~~	Jamie Vaughn	Additional CRA
~~	~~~	Jill Jacinto	Additional CRA
~~	~~~	Joelle DuMontier	Additional CRA
~~	~~~	Julia Giovannini	Additional CRA
~~	~~~	Kathi Klapp	Additional CRA
~~	~~~	Kathleen Marek	Additional CRA
~~	~~~	Kathy Cherepak	Additional CRA
~~	~~~	Kathy Trakalo	Additional CRA
~~	~~~	KuanLin Li	Additional CRA
~~	~~~	Laurie Mills	Additional CRA
~~	~~~	Leanne Anderson	Additional CRA
~~	~~~	Lisa Peak	Additional CRA
~~	~~~	Lori Walker	Additional CRA
~~	~~~	Mandy Squires	Additional CRA
~~	~~~	Maria Morales	Additional CRA
~~	~~~	Marilyn Pawl	Additional CRA
~~	~~~	Megan Ridler	Additional CRA
~~	~~~	Melanie R Watson	Additional CRA
~~	~~~	Patricia Benjaminson	Additional CRA
~~	~~~	Priscilla Santos	Additional CRA
~~	~~~	Rayleen Rudnicki	Additional CRA
~~	~~~	Rhonda Nichol	Additional CRA
~~	~~~	Robyn Guarino	Additional CRA
~~	~~~	Rose Woloshyn	Additional CRA
~~	~~~	Sandy Yap	Additional CRA

INVESTIGATORS AND COLLABORATORS: SITE STAFF
Staff on site delegation logs

Country	Site	Names	Role
~~	~~~	Shauna Priebe	Additional CRA
~~	~~~	Stacey Rizzuto	Additional CRA
~~	~~~	Taehee Ann	Additional CRA
~~	~~~	Theresa Moore	Additional CRA
~~	~~~	Tiffani Eska	Additional CRA
~~	~~~	Tracy Chornopyski	Additional CRA
~~	~~~	Valerie Kuzyk	Additional CRA
~~	~~~	Zeljka Bakija	Additional CRA
Denmark	Aalborg: Aalborg University Hosp	Ms Mette Moe Kempel	Site PI
~~	~~~	Mr Niels Harving	Clinical/Surgical
~~	~~~	Mr Finn Hejlesen	Point of contact (1st)
~~	~~~	Ms Kirsten Steffensen	Research Nurse
Denmark	Aarhus: Aarhus Kommunehospital	Dr Simon Buus	Site PI
~~	~~~	Dr Henrik Schultz	Clinical/Surgical
~~	~~~	Dr Henrik Schultz	Clinical/Surgical
~~	~~~	Dr Kirsten Fode	Clinical/Surgical
~~	~~~	Dr Lise Bentzen	Clinical/Surgical
~~	~~~	Dr Michael Borre	Clinical/Surgical
~~	~~~	Dr Slavka Lucakova	Clinical/Surgical
~~	~~~	Dr Yasmin Lassen	Clinical/Surgical
~~	~~~	Ms Helle Lemng Kruse	Point of contact (1st)
~~	~~~	Ms Helle Nørmark Iversen	Point of contact (1st)
~~	~~~	Ms Vibeke Laursen	Point of contact (1st)
~~	~~~	Ms Birgit Kaa Bach	Research Nurse
Denmark	Copenhagen: Rigshospitalet University Hosp	Dr Peter Meidhal Petersen	Site PI
~~	~~~	Dr Anne Juel Christensen	Clinical/Surgical
~~	~~~	Prof Klaus Brasso	Clinical/Surgical
~~	~~~	Ms Anne Sofie Lunau	Point of contact (1st)
~~	~~~	Ms Sine Stilling	Point of contact (1st)
~~	~~~	Ms Stine Thim	Point of contact (1st)
~~	~~~	Ms Karina Klovgaard	Point of contact (2nd)
~~	~~~	Ms Anna Torin Lehmann	Research Nurse
~~	~~~	Ms Lisa Gruschy	Research Nurse
~~	~~~	Ms Lone Lund Poder	Research Nurse

INVESTIGATORS AND COLLABORATORS: SITE STAFF

Staff on site delegation logs

Country	Site	Names	Role
~~	~~~	Ms Maria Vandborg	Research Nurse
~~	~~~	Ms Trine Moller Christensen	Research Nurse
~~	~~~	Ms Kristine Ribers	Other
Denmark	Herlev: Amtssygehuset i Herlev (Herlev University Hosp)	Dr Henriette Lindberg	Site PI
~~	~~~	Ms Kathrine Friser Kokholm	Point of contact (1st)
~~	~~~	Ms Tine Christensen	Point of contact (1st)
~~	~~~	Ms Charlotte Saxe	Trial Coordinator
~~	~~~	Ms Eva Rønnengart	Trial Coordinator
~~	~~~	Kirstine Nybom	Other
Ireland	Galway: University College Hosp Galway	Mr Garret Durkan	Site PI
~~	~~~	Dr Joseph Martin	Clinical/Surgical
~~	~~~	Mr Eamonn Rogers	Clinical/Surgical
~~	~~~	Prof Frank Sullivan	Clinical/Surgical
~~	~~~	Ms Chiaw Woon Teh	Point of contact (1st)
~~	~~~	Ms Marian Jennings	Point of contact (1st)
~~	~~~	Ms Ann Wright	Point of contact (2nd)
~~	~~~	Ms Catriona Mahoney	Research Nurse
~~	~~~	Ms Liz Raftery	Research Nurse
~~	~~~	Ms Mary Marg Byrne	Research Nurse
~~	~~~	Ms Veronica McInerney	Research Nurse
~~	~~~	Ms Emma Deenihan	Administrator
~~	~~~	Ms Maria Spillane	Other
UK	Aberdeen: Aberdeen Royal Inf	Dr Graham MacDonald	Site PI
~~	~~~	Dr Donald Bissett	Clinical/Surgical
~~	~~~	Dr Judith Grant	Clinical/Surgical
~~	~~~	Mr Rory Lynch	Point of contact (1st)
~~	~~~	Mrs Rachel Moir	Point of contact (1st)
~~	~~~	Ms Marie McWilliam	Point of contact (1st)
~~	~~~	Ms Kirsty Shearer	Point of contact (2nd)
~~	~~~	Mrs Margaret Smith	Research Nurse
~~	~~~	Ms Sue Rodwell	Research Nurse
~~	~~~	Ms Shelagh Bonner-Shand	Trial Coordinator
UK	Airedale: Airedale General Hosp	Dr Ganesan Jeyasanger	Site PI
~~	~~~	Dr Ann Henry	Clinical/Surgical

INVESTIGATORS AND COLLABORATORS: SITE STAFF

Staff on site delegation logs

Country	Site	Names	Role
~~	~~~	Dr Nathalie Casanova	Clinical/Surgical
~~	~~~	Dr Simon Brown	Clinical/Surgical
~~	~~~	Mrs Alison Shaw	Point of contact (1st)
~~	~~~	Mrs Pipa Hill	Point of contact (1st)
~~	~~~	Ms Helen Henson	Point of contact (1st)
~~	~~~	Ms Maxine Briggs	Point of contact (2nd)
~~	~~~	Mrs Sharron Parkinson	Research Nurse
~~	~~~	Ms Judy McAlister	Research Nurse
~~	~~~	Ms Louise Binns	Research Nurse
~~	~~~	Mrs Jasmine Hartley	Research Asst
~~	~~~	Dr Katy Clark	Other
UK	Aylesbury: Stoke Mandeville Hosp	Dr Katherine Hyde	Site PI
~~	~~~	Dr Ami Sabharwal	Clinical/Surgical
~~	~~~	Dr Andrew Weaver	Clinical/Surgical
~~	~~~	Dr Christopher Alcock	Clinical/Surgical
~~	~~~	Dr Joanne Brady	Clinical/Surgical
~~	~~~	Dr Niki Panakis	Clinical/Surgical
~~	~~~	Dr Philip Camilleri	Clinical/Surgical
~~	~~~	Dr Thinn Pwint	Clinical/Surgical
~~	~~~	Dr Janice Carpenter	Point of contact (1st)
~~	~~~	Mrs Alice Ngumo	Point of contact (1st)
~~	~~~	Mrs Cheryl Padilla-Harris	Research Nurse
~~	~~~	Mrs Gail Varley	Research Nurse
~~	~~~	Mrs Tracey Stammers	Research Nurse
~~	~~~	Ms Emma Hogbin	Research Nurse
~~	~~~	Ms Hazel Wynn	Research Nurse
~~	~~~	Ms Helena Stone	Research Nurse
~~	~~~	Ms Roisin Kavanagh	Pharmacist
UK	Ayr: Ayr Hosp	Dr Nicholas Macleod	Site PI
~~	~~~	Dr Aisha Tufail	Clinical/Surgical
~~	~~~	Dr Aqilah Othman	Clinical/Surgical
~~	~~~	Dr Hilary Glen	Clinical/Surgical
~~	~~~	Dr Janet Graham	Clinical/Surgical
~~	~~~	Dr Jawaher Ansari	Clinical/Surgical

INVESTIGATORS AND COLLABORATORS: SITE STAFF
Staff on site delegation logs

Country	Site	Names	Role
~~	~~~	Dr Maryon Hardie	Clinical/Surgical
~~	~~~	Dr Nick Mcleod	Clinical/Surgical
~~	~~~	Dr Patricia Roxburgh	Clinical/Surgical
~~	~~~	Dr Rana Mahmood	Clinical/Surgical
~~	~~~	Miss Kristy Ross	Clinical/Surgical
~~	~~~	Ms Claudia Coubrough	Point of contact (1st)
~~	~~~	Ms Claudia Turley	Point of contact (1st)
~~	~~~	Mr Mark Wilson	Research Nurse
~~	~~~	Mrs Jane McClements	Research Nurse
~~	~~~	Ms Chloe Cowan	Research Nurse
~~	~~~	Ms Margaret McKernan	Research Nurse
~~	~~~	Ms Maureen Templeton	Research Nurse
~~	~~~	Mr Philip Cannon	Trial Coordinator
~~	~~~	Ms Clare Love	Data Manager
~~	~~~	Ms Elaine Allan	Administrator
~~	~~~	Mr Brian McGlynn	Other
~~	~~~	Mr Ross Carruthers	Other
UK	Bangor: Ysbyty Gwynedd	Dr Nikhil Oommen	Site PI
~~	~~~	Dr Rachel Williams	Clinical/Surgical
~~	~~~	Dr Thomas Coventry	Clinical/Surgical
~~	~~~	Dr Zulfiqer Ali	Clinical/Surgical
~~	~~~	Mr Gareth Jones	Point of contact (1st)
~~	~~~	Ms Dianne Thomas	Point of contact (1st)
~~	~~~	Mrs Caryl Butterworth	Research Nurse
~~	~~~	Ms Rebecca Burns	Research Nurse
~~	~~~	Ms Wendy Saxton	Research Nurse
~~	~~~	Sister Hayley Tapping	Research Nurse
~~	~~~	Mr Sion Lewis	Trial Coordinator
~~	~~~	Mrs Angela Evans	Trial Coordinator
~~	~~~	Miss Kelly Andrews	Administrator
~~	~~~	Miss Rachel Thomas	Administrator
~~	~~~	Ms Beth Walker	MDT coordinator
~~	~~~	Ms Susan Owen	Radiographer
UK	Barnstaple: North Devon District Hosp	Dr Mohini Varughese	Site PI

INVESTIGATORS AND COLLABORATORS: SITE STAFF
Staff on site delegation logs

Country	Site	Names	Role
~~	~~~	Dr Anne McCormack	Clinical/Surgical
~~	~~~	Dr Denise Sheehan	Clinical/Surgical
~~	~~~	Dr Elizabeth Kershaw	Clinical/Surgical
~~	~~~	Dr Elizabeth Toy	Clinical/Surgical
~~	~~~	Dr Maria Martinez	Clinical/Surgical
~~	~~~	Dr Victoria Ford	Clinical/Surgical
~~	~~~	Ms Becky Holbrook	Point of contact (1st)
~~	~~~	Mrs Susan Collard	Research Nurse
~~	~~~	Mrs Samantha Ley	Trial Coordinator
~~	~~~	Ms Frances Goodhind	Pharmacist
~~	~~~	Mrs Lynne Van-Koutrik	Other
UK	Basingstoke: Basingstoke & North Hampshire Hosp	Dr Richard Shaffer	Site PI
~~	~~~	Dr Sangeeta Paisey	Site PI
~~	~~~	Dr Hilawati Yusof	Clinical/Surgical
~~	~~~	Dr Katherine Aitken	Clinical/Surgical
~~	~~~	Dr Rosalyne Westley	Clinical/Surgical
~~	~~~	Dr Teresa Guerrero-Urbano	Clinical/Surgical
~~	~~~	Beata Krysta	Point of contact (1st)
~~	~~~	Mrs Abigail Edwards	Point of contact (1st)
~~	~~~	Mr Godfrey Bownie-Mukumbu	Point of contact (2nd)
~~	~~~	Miss Rachel Bryan	Research Nurse
~~	~~~	Mrs Jackie Smith	Research Nurse
~~	~~~	Ms Liz Happle	Research Nurse
~~	~~~	Mrs Adrienn Fazekasne Fulep	Trial Coordinator
~~	~~~	Dr David Barlow	Data Manager
~~	~~~	Miss Louise Beattie	Data Manager
~~	~~~	Ms Victoria Corner	Research Asst
~~	~~~	Ms Julie Gwilt	Administrator
~~	~~~	Ms Dee Jackman	MDT coordinator
UK	Bath: Royal United Hosp	Dr Olivera Frim	Site PI
~~	~~~	Dr Abigail Jenner	Clinical/Surgical
~~	~~~	Dr Christine Elwell	Clinical/Surgical
~~	~~~	Dr Hugh Newman	Clinical/Surgical
~~	~~~	Dr Jessica Mason	Clinical/Surgical

INVESTIGATORS AND COLLABORATORS: SITE STAFF

Staff on site delegation logs

Country	Site	Names	Role
~~	~~~	Dr Mark Beresford	Clinical/Surgical
~~	~~~	Dr Susan Masson	Clinical/Surgical
~~	~~~	Dr Zoe Hudson	Clinical/Surgical
~~	~~~	Mr Jonathan McFarlane	Clinical/Surgical
~~	~~~	Mr Mark Mantle	Clinical/Surgical
~~	~~~	Miss Abigail Pocock	Point of contact (1st)
~~	~~~	Mrs Samantha Curtis	Point of contact (1st)
~~	~~~	Ms Bryony Robertson	Point of contact (2nd)
~~	~~~	Mrs Beatrice Hamilton	Research Nurse
~~	~~~	Mrs Christine Cox	Research Nurse
~~	~~~	Mrs Rowan Appleby	Research Nurse
~~	~~~	Mrs Ruth Brydon-Hill	Research Nurse
~~	~~~	Ms Rebecca Wassall	Research Nurse
~~	~~~	Sister Tania Williams (Née Allen)	Research Nurse
~~	~~~	Ms Eve Tomlinson	Administrator
~~	~~~	Miss Joanne Avis	Other
UK	Belfast: Belfast City Hosp	Prof Joe O'Sullivan	Site PI
~~	~~~	Dr Aiden Cole	Clinical/Surgical
~~	~~~	Dr Darren Mitchell	Clinical/Surgical
~~	~~~	Dr David Stewart	Clinical/Surgical
~~	~~~	Dr Fionnuala Houghton	Clinical/Surgical
~~	~~~	Dr Jackie Harney	Clinical/Surgical
~~	~~~	Dr Jonathan McAleese	Clinical/Surgical
~~	~~~	Dr Poh Lin Shum	Clinical/Surgical
~~	~~~	Dr Ruth Johnston	Clinical/Surgical
~~	~~~	Dr Stephen Stranex	Clinical/Surgical
~~	~~~	Dr Suneil Jain	Clinical/Surgical
~~	~~~	Mr Chris Hagan	Clinical/Surgical
~~	~~~	Mr Nambi Rajan	Clinical/Surgical
~~	~~~	Mr Patrick Keane	Clinical/Surgical
~~	~~~	Mr Peter Clarke	Point of contact (1st)
~~	~~~	Mrs Gail Gilchrist	Point of contact (2nd)
~~	~~~	Mrs Grace Totten	Point of contact (2nd)
~~	~~~	Ms Emma Hanna	Data Manager

INVESTIGATORS AND COLLABORATORS: SITE STAFF

Staff on site delegation logs

Country	Site	Names	Role
~~	~~~	Miss Jane Cousins	Radiographer
~~	~~~	Miss Karen McKenna	Radiographer
~~	~~~	Miss Stacey Murray	Radiographer
~~	~~~	Mrs Stacey Hetherington	Radiographer
~~	~~~	Ms Grace Brown	Radiographer
~~	~~~	Dr Ciara Lyons	Other
~~	~~~	Mrs Jo McAllister	Other
~~	~~~	Ms Sharon Hynds	Other
UK	Birmingham: Birmingham Heartlands Hosp	Dr Anjali Zarkar	Site PI
~~	~~~	Mrs Kavitha Shetty	Clinical/Surgical
~~	~~~	Ms Rachel Lokes	Point of contact (1st)
~~	~~~	Ms Sundip Sohanpal	Point of contact (1st)
~~	~~~	Mrs Madhura Chandrashekara	Point of contact (2nd)
~~	~~~	Ms Ann Schumacher	Point of contact (2nd)
~~	~~~	Mrs Ellen Drew	Research Nurse
~~	~~~	Mrs Tina Gamble	Research Nurse
~~	~~~	Ms Mary (Ellen) Drew	Research Nurse
~~	~~~	Mr James Whitehouse	Trial Coordinator
~~	~~~	Mrs Julie Fletcher	Trial Coordinator
UK	Birmingham: Good Hope Hosp	Dr Daniel Ford	Site PI
~~	~~~	Dr John Glaholm	Clinical/Surgical
~~	~~~	Miss Rachael O’Beney	Point of contact (1st)
~~	~~~	Mr Daniel Lenton	Point of contact (1st)
~~	~~~	Mrs Helen Thomas	Research Nurse
~~	~~~	Ms Helen Taylor	Research Nurse
~~	~~~	Mr James Whitehouse	Trial Coordinator
~~	~~~	Ms Sundip Sohanpal	Data Manager
UK	Birmingham: Queen Elizabeth Hosp (Birmingham)	Dr Anjali Zarkar	Site PI
~~	~~~	Dr Ahmed El-Modir	Clinical/Surgical
~~	~~~	Dr Daniel Ford	Clinical/Surgical
~~	~~~	Dr John Glaholm	Clinical/Surgical
~~	~~~	Mr A Doherty	Clinical/Surgical
~~	~~~	Prof Nicholas James	Clinical/Surgical
~~	~~~	Miss Tarandip Samra	Point of contact (1st)

INVESTIGATORS AND COLLABORATORS: SITE STAFF
Staff on site delegation logs

Country	Site	Names	Role
~~	~~~	Mrs Liliam Ross	Point of contact (1st)
~~	~~~	Ms Rachel Lokes	Point of contact (1st)
~~	~~~	Miss Josephine Marange	Research Nurse
~~	~~~	Miss Rebekah Stephens	Research Nurse
~~	~~~	Mr Andrew Morrison	Trial Coordinator
~~	~~~	Mr Phillip Watson-Jones	Trial Coordinator
~~	~~~	Miss Melanie Gunn	Data Manager
~~	~~~	Miss Zhane Peterkin	Data Manager
~~	~~~	Mr George Preece	Data Manager
~~	~~~	Mrs Aliyah Mannan	Data Manager
~~	~~~	Mr Zayn Qureshi	MDT coordinator
~~	~~~	Mrs Elizabeth Southgate	Radiographer
~~	~~~	Mrs Maryanne Okubanjo	Radiographer
~~	~~~	Mrs Samantha Chetiyawardana	Radiographer
~~	~~~	Ms Darlletta Olaniyi-Oduntan	Radiographer
~~	~~~	Ms Liz Kedge	Radiographer
~~	~~~	Dr Robert Stevenson	Other
UK	Blackburn: Burnley General Hosp	Dr Omi Parikh	Site PI
~~	~~~	Dr Catherine Thompson	Clinical/Surgical
~~	~~~	Dr Danya Abdulwahid	Clinical/Surgical
~~	~~~	Dr Imran Haidar	Clinical/Surgical
~~	~~~	Dr Jennifer King	Clinical/Surgical
~~	~~~	Dr Marcus Wise	Clinical/Surgical
~~	~~~	Dr Natalie Charnley	Clinical/Surgical
~~	~~~	Dr Parth Desai	Clinical/Surgical
~~	~~~	Mrs Jacqueline Thomas	Point of contact (1st)
~~	~~~	Ms Jeanette Hargreaves	Point of contact (1st)
~~	~~~	Mrs Janet Ryan-Smith	Point of contact (2nd)
~~	~~~	Mrs Angela Hugill	Research Nurse
~~	~~~	Mrs Helen Frankland	Research Nurse
~~	~~~	Mrs Lynsey Waring	Research Nurse
~~	~~~	Ms Diane Forrest	Research Nurse
~~	~~~	Ms Jan Flaherty	Research Nurse
~~	~~~	Ms Karen Beard	Research Nurse

INVESTIGATORS AND COLLABORATORS: SITE STAFF
Staff on site delegation logs

Country	Site	Names	Role
~~	~~~	Ms Karen Jewers	Research Nurse
~~	~~~	Ms Karen Riley	Research Nurse
~~	~~~	Ms Sarah Ainsworth	Research Nurse
~~	~~~	Ms Sarah Keith	Research Nurse
~~	~~~	Mrs Sue Ashworth	Trial Coordinator
~~	~~~	Miss Bethany Fielding	Other
~~	~~~	Mr Stephen Kilroy	Other
~~	~~~	Ms Joanne Henry	Other
~~	~~~	Ms Vivienne Tickle	Other
UK	Blackburn: Royal Blackburn Hosp	Dr Omi Parikh	Site PI
~~	~~~	Dr Catherine Thompson	Clinical/Surgical
~~	~~~	Dr Danya Abdulwahid	Clinical/Surgical
~~	~~~	Dr Imran Haidar	Clinical/Surgical
~~	~~~	Dr Jennifer King	Clinical/Surgical
~~	~~~	Dr Marcus Wise	Clinical/Surgical
~~	~~~	Dr Parth Desai	Clinical/Surgical
~~	~~~	Ms Jeanette Hargreaves	Point of contact (1st)
~~	~~~	Mrs Janet Ryan-Smith	Point of contact (2nd)
~~	~~~	Mrs Lynsey Waring	Point of contact (2nd)
~~	~~~	Mrs Angela Hugill	Research Nurse
~~	~~~	Mrs Helen Frankland	Research Nurse
~~	~~~	Ms Diane Forrest	Research Nurse
~~	~~~	Ms Jan Flaherty	Research Nurse
~~	~~~	Ms Karen Beard	Research Nurse
~~	~~~	Ms Karen Jewers	Research Nurse
~~	~~~	Ms Karen Riley	Research Nurse
~~	~~~	Ms Louise Dawson	Research Nurse
~~	~~~	Ms Sarah Ainsworth	Research Nurse
~~	~~~	Ms Sarah Keith	Research Nurse
~~	~~~	Mrs Julie Ditchfield	Trial Coordinator
~~	~~~	Mrs Sue Ashworth	Trial Coordinator
~~	~~~	Miss Bethany Fielding	Other
~~	~~~	Mr Stephen Kilroy	Other
~~	~~~	Ms Joanne Henry	Other

INVESTIGATORS AND COLLABORATORS: SITE STAFF
Staff on site delegation logs

Country	Site	Names	Role
~~	~~~	Ms Vivienne Tickle	Other
UK	Bodelwyddan: Glan Clwyd Hosp	Dr Nikhil Oommen	Site PI
~~	~~~	Dr Aileen Flavin	Clinical/Surgical
~~	~~~	Dr Amir Al-Samarraie	Clinical/Surgical
~~	~~~	Dr Zulfiqer Ali	Clinical/Surgical
~~	~~~	Mr H Toussi	Clinical/Surgical
~~	~~~	Mr Vaiku Srinivasan	Clinical/Surgical
~~	~~~	Miss Rachel Manley	Point of contact (1st)
~~	~~~	Mrs Jane Heron	Point of contact (1st)
~~	~~~	Mrs Llinos Davies	Point of contact (1st)
~~	~~~	Ms Heather Thomas	Point of contact (1st)
~~	~~~	Mrs Charley-Anne Rutter	Research Nurse
~~	~~~	Mrs Joanne Lewis	Research Nurse
~~	~~~	Ms Jane Stockport	Research Nurse
~~	~~~	Sister Hayley Tapping	Research Nurse
~~	~~~	Miss Faye Hughes	Trial Coordinator
~~	~~~	Mrs Kim Jackson	Trial Coordinator
~~	~~~	Ms Annette Bolger	Data Manager
~~	~~~	Ms Cathryn Wood	Radiographer
~~	~~~	Dr Nick Smith	Other
UK	Boston: Pilgrim Hosp	Dr Thiagarajan Sreenivasan	Site PI
~~	~~~	Dr Miguel Panades	Clinical/Surgical
~~	~~~	Mr Simon Archer	Point of contact (1st)
~~	~~~	Mrs Kinga Szymiczek	Point of contact (1st)
~~	~~~	Ms Amy Kirkby	Point of contact (1st)
~~	~~~	Ms Joanne Fletcher	Point of contact (1st)
~~	~~~	Ms Tara Lawrence nee Palmer	Point of contact (1st)
~~	~~~	Miss Karen Metcalf	Research Nurse
~~	~~~	Mrs Isobel Thomas	Research Nurse
~~	~~~	Mrs Victoria Knight (n. Sherburn)	Research Nurse
~~	~~~	Ms Anita Young	Research Nurse
~~	~~~	Ms Helen Ginnelly	Research Nurse
~~	~~~	Mrs Beverley Mashegede	Data Manager
UK	Bournemouth: Royal Bournemouth Hosp	Dr Sue Brock	Site PI

INVESTIGATORS AND COLLABORATORS: SITE STAFF
Staff on site delegation logs

Country	Site	Names	Role
~~	~~~	Dr Joseph Davies	Clinical/Surgical
~~	~~~	Ms Sarah Savage	Point of contact (1st)
~~	~~~	Mrs Nicky Naraine	Research Nurse
~~	~~~	Ms Eve Broadley	Research Nurse
~~	~~~	Ms Stephanie Jones	Research Nurse
~~	~~~	Miss Laura Purandare	Data Manager
~~	~~~	Miss Taslima Rabbi	Data Manager
~~	~~~	Mr Luke Vamplew	Data Manager
~~	~~~	Ms Kate Preece	Data Manager
~~	~~~	Ms Ruby McCully	Administrator
UK	Bradford: Bradford Royal Inf	Dr Sree Rodda	Site PI
~~	~~~	Dr Ann Henry	Clinical/Surgical
~~	~~~	Dr Lisa Owen	Clinical/Surgical
~~	~~~	Dr Louise Karsera	Clinical/Surgical
~~	~~~	Dr Mark Teo	Clinical/Surgical
~~	~~~	Ms Chandran Nallathambi	Clinical/Surgical
~~	~~~	Ms Shefali Parikh	Clinical/Surgical
~~	~~~	Miss Jannika Lazarte	Point of contact (1st)
~~	~~~	Mrs Jane Sewell	Point of contact (1st)
~~	~~~	Miss Lucille Kenyon	Research Nurse
~~	~~~	Mrs Hayley Inman	Research Nurse
~~	~~~	Mrs Helen Robertshaw	Research Nurse
~~	~~~	Ms Anne Marie Kay	Research Nurse
~~	~~~	Ms Linda Bamford	Research Nurse
~~	~~~	Miss Amy Pendrill	Data Manager
~~	~~~	Mr Richard Benton	Data Manager
~~	~~~	Miss Eleanor Waldron	Administrator
~~	~~~	Mrs Dawn McNulty	Administrator
~~	~~~	Ms Eleanor Moore	Administrator
~~	~~~	Ms Lauren Cotterill	MDT coordinator
~~	~~~	Ms Deirdre Naylor	Pharmacist
~~	~~~	Ms Joanne Price	Pharmacist
UK	Brighton: Royal Sussex County Hosp	Dr Angus Robinson	Site PI
~~	~~~	Dr Andrew Webb	Clinical/Surgical

INVESTIGATORS AND COLLABORATORS: SITE STAFF
Staff on site delegation logs

Country	Site	Names	Role
~~	~~~	Dr Ashok Nikapota	Clinical/Surgical
~~	~~~	Dr David Bloomfield	Clinical/Surgical
~~	~~~	Dr Duncan Gilbert	Clinical/Surgical
~~	~~~	Dr Fiona McKinna	Clinical/Surgical
~~	~~~	Dr Marie Wilkins	Clinical/Surgical
~~	~~~	Miss Catherine Hunter	Point of contact (1st)
~~	~~~	Mr Paul Frattaroli	Point of contact (1st)
~~	~~~	Ms Jessica Dlodlo	Point of contact (1st)
~~	~~~	Ms Joanne Brown	Point of contact (1st)
~~	~~~	Mr Andrew Hart	Research Nurse
~~	~~~	Ms Madalena Leitao	Research Nurse
~~	~~~	Miss Jean Tremlett	Trial Coordinator
~~	~~~	Ms Bobbie Young	Trial Coordinator
~~	~~~	Ms Jane Peterson	Trial Coordinator
~~	~~~	Ms Tracy Blythe	Trial Coordinator
~~	~~~	Miss Samantha Hodges	Data Manager
~~	~~~	Ms Carla Chambers	Data Manager
~~	~~~	Ms Ranee Lactao	Data Manager
~~	~~~	Mr Simon Hooper	Radiographer
~~	~~~	Ms Philippa Carr	Radiographer
UK	Bristol: Bristol Haematology & Oncology Centre	Dr Amit Bahl	Site PI
~~	~~~	Dr Amarnath Challipalli	Clinical/Surgical
~~	~~~	Dr Mark Beresford	Clinical/Surgical
~~	~~~	Dr Paula Wilson	Clinical/Surgical
~~	~~~	Dr Serena Hilman	Clinical/Surgical
~~	~~~	Dr Susan Masson	Clinical/Surgical
~~	~~~	Mr David Gillatt	Clinical/Surgical
~~	~~~	Mr Marc Coe	Point of contact (1st)
~~	~~~	Mr Martin Woods	Point of contact (1st)
~~	~~~	Ms Vivienne Lee	Point of contact (1st)
~~	~~~	Mr Matt Baxter	Point of contact (2nd)
~~	~~~	Miss Mary Kisanga	Research Nurse
~~	~~~	Mr Peter Robertson	Research Nurse
~~	~~~	Mrs Dorothy Griffiths	Research Nurse

INVESTIGATORS AND COLLABORATORS: SITE STAFF
Staff on site delegation logs

Country	Site	Names	Role
~~	~~~	Miss Amelia Lowe	Trial Coordinator
~~	~~~	Ms Helen Saldanha	Trial Coordinator
~~	~~~	Ms Rhiannon Macefield	Trial Coordinator
~~	~~~	Ms Ruth Pegler	Trial Coordinator
~~	~~~	Ms Verity Henson (nee Savidge)	Trial Coordinator
~~	~~~	Mr Robert Hollister	Data Manager
~~	~~~	Ms Laura Sims	Data Manager
~~	~~~	Mrs Lucie Wheeler	Administrator
~~	~~~	Miss Mary Simmonds	Radiographer
~~	~~~	Mrs Sarah Zelley	Radiographer
~~	~~~	Mrs Sue Cowley	Radiographer
~~	~~~	Mrs Sue Yarrow	Radiographer
~~	~~~	Ms Dawn Bowers	Radiographer
UK	Bristol: Southmead Hosp	Mr Raj Persad	Site PI
~~	~~~	Dr Amarnath Challipalli	Clinical/Surgical
~~	~~~	Dr Amit Bahl	Clinical/Surgical
~~	~~~	Dr Chris Herbert	Clinical/Surgical
~~	~~~	Dr Mark Beresford	Clinical/Surgical
~~	~~~	Dr Paula Wilson	Clinical/Surgical
~~	~~~	Mr Anthony Koupparis	Clinical/Surgical
~~	~~~	Mr David Gillatt	Clinical/Surgical
~~	~~~	Mr Edward Rowe	Clinical/Surgical
~~	~~~	Mrs Samantha Clarke	Point of contact (1st)
~~	~~~	Ms Emily Perry	Point of contact (1st)
~~	~~~	Ms Lyndsey Johnson	Point of contact (1st)
~~	~~~	Ms Marta Cobos-Arrivabene	Point of contact (1st)
~~	~~~	Ms Rebecca Cousins	Point of contact (1st)
~~	~~~	Ms Victoria Garner	Point of contact (1st)
~~	~~~	Ms Carol Brain	Point of contact (2nd)
~~	~~~	Ms Constance Shiridzinomwa	Point of contact (2nd)
~~	~~~	Ms Kathryn Jones	Point of contact (2nd)
~~	~~~	Mrs Sarah Kirkby	Research Nurse
~~	~~~	Mrs Suriya Kirkpatrick	Research Nurse
~~	~~~	Ms Charlotte Phipps	Research Nurse

INVESTIGATORS AND COLLABORATORS: SITE STAFF
Staff on site delegation logs

Country	Site	Names	Role
~~	~~~	Ms Helen Corderoy	Research Nurse
~~	~~~	Ms Carolyn Smith	Trial Coordinator
~~	~~~	Ms Kate Alderton	Trial Coordinator
~~	~~~	Mrs Kerry Richmond-Russell	Data Manager
~~	~~~	Ms Ann Treasure	Administrator
~~	~~~	Miss Clare Wyatt	MDT coordinator
~~	~~~	Ms Courtney Manning	MDT coordinator
~~	~~~	Dr Jon Oxley	Other
~~	~~~	Mr Luke Shelton	Other
~~	~~~	Mr Mohamed Abd Alazeez	Other
~~	~~~	Ms Gail Kemp	Other
~~	~~~	Ms Helen Chilcott	Other
UK	Cambridge: Addenbrooke's Hosp	Dr Yvonne Rimmer	Site PI
~~	~~~	Dr Cathryn Woodward	Clinical/Surgical
~~	~~~	Dr Danish Mazhar	Clinical/Surgical
~~	~~~	Dr Helen Patterson	Clinical/Surgical
~~	~~~	Dr Luke Hughes-Davies	Clinical/Surgical
~~	~~~	Dr Ramya Ramanujachar	Clinical/Surgical
~~	~~~	Dr Richard Benson	Clinical/Surgical
~~	~~~	Dr Robert Thomas	Clinical/Surgical
~~	~~~	Dr Sanjay Raj	Clinical/Surgical
~~	~~~	Dr Simon Russell	Clinical/Surgical
~~	~~~	Ms Juliette Harnwell	Point of contact (1st)
~~	~~~	Mr Matthew Stone	Point of contact (2nd)
~~	~~~	Miss Vicky Joslin	Trial Coordinator
~~	~~~	Mrs Vanessa Goss	Trial Coordinator
~~	~~~	Ms Angelique Laubscher	Administrator
~~	~~~	Ms Ellie Couch	Administrator
~~	~~~	Ms Katherine Beesley	Administrator
~~	~~~	Mrs Sue Foxwell	MDT coordinator
~~	~~~	Miss Nicola Robinson	Radiographer
~~	~~~	Mrs Jo Treeby	Radiographer
~~	~~~	Ms Kathryn Vickery	Radiographer
UK	Canterbury: Kent & Canterbury Hosp	Dr Rakesh Raman	Site PI

INVESTIGATORS AND COLLABORATORS: SITE STAFF
Staff on site delegation logs

Country	Site	Names	Role
~~	~~~	Dr Albert Edwards	Clinical/Surgical
~~	~~~	Dr Alice Rendall	Clinical/Surgical
~~	~~~	Dr Carys Thomas	Clinical/Surgical
~~	~~~	Dr Christos Mikropoulos	Clinical/Surgical
~~	~~~	Dr Clary Evans	Clinical/Surgical
~~	~~~	Dr Gemma Hegarty	Clinical/Surgical
~~	~~~	Dr Ilyas Ahmed	Clinical/Surgical
~~	~~~	Dr Ioannis Trigonis	Clinical/Surgical
~~	~~~	Dr Jessica Gough	Clinical/Surgical
~~	~~~	Dr Jessica Little	Clinical/Surgical
~~	~~~	Dr Joao Galante	Clinical/Surgical
~~	~~~	Dr Kannon Nathan	Clinical/Surgical
~~	~~~	Dr Natasha Mithal	Clinical/Surgical
~~	~~~	Dr Patryk Brulinski	Clinical/Surgical
~~	~~~	Dr Rohit Malde	Clinical/Surgical
~~	~~~	Dr Van Sim	Clinical/Surgical
~~	~~~	Mr Arafat Mirza	Clinical/Surgical
~~	~~~	Mrs Laura Kehoe	Point of contact (1st)
~~	~~~	Mrs Laura Mould	Point of contact (1st)
~~	~~~	Mrs Louise Gladwell	Point of contact (1st)
~~	~~~	Mrs Julie Buckley	Research Nurse
~~	~~~	Ms Carolyn Hargreaves	Research Nurse
~~	~~~	Ms Elizabeth Williamson	Research Nurse
~~	~~~	Ms Joanne Williams	Research Nurse
~~	~~~	Ms Julie-Ann Davies	Research Nurse
~~	~~~	Ms Michelle Swann	Research Nurse
~~	~~~	Ms Pauline Wood	Research Nurse
~~	~~~	Ms Rachel Ryan	Research Nurse
~~	~~~	Ms Susan Drakeley	Research Nurse
~~	~~~	Sister Denise Crawford	Research Nurse
~~	~~~	Mrs Rachel Larkins	Trial Coordinator
~~	~~~	Ms Bonny Appleby	Trial Coordinator
~~	~~~	Ms Hilary Zurakovsky	Trial Coordinator
~~	~~~	Ms Karen Robinson	Trial Coordinator

INVESTIGATORS AND COLLABORATORS: SITE STAFF
Staff on site delegation logs

Country	Site	Names	Role
~~	~~~	Ms Marian Wood	Trial Coordinator
~~	~~~	Ms Sharon Middleton	Trial Coordinator
~~	~~~	Mrs Laura Mould	Administrator
~~	~~~	Ms Lorraine Tasker	MDT coordinator
~~	~~~	Miss Katy Taylor	Radiographer
~~	~~~	Miss Natalie Catt	Radiographer
~~	~~~	Ms Helen Coppins	Radiographer
~~	~~~	Ms Karen Walker	Radiographer
~~	~~~	Ms Margaret Lipsham	Radiographer
UK	Cardiff: University Hosp of Wales	Mr Krishna Narahari	Site PI
~~	~~~	Dr John Staffurth	Clinical/Surgical
~~	~~~	Mr Owen Hughes	Clinical/Surgical
~~	~~~	Ms Hin Fan Chan	Clinical/Surgical
~~	~~~	Prof Howard Kynaston	Clinical/Surgical
~~	~~~	Prof Malcolm Mason	Clinical/Surgical
~~	~~~	Miss Elizabeth Bois (nee Harris)	Point of contact (1st)
~~	~~~	Ms Colette Clements	Point of contact (1st)
~~	~~~	Mr Kevin Pearse	Research Nurse
~~	~~~	Mrs Clare Jones	Research Nurse
~~	~~~	Mrs Loveness Chikopela	Research Nurse
~~	~~~	Mrs Samantha Holliday	Research Nurse
~~	~~~	Mrs Helen Clark	Data Manager
UK	Chelmsford: Broomfield Hosp	Dr Abdel Hamid	Site PI
~~	~~~	Dr Kiran Kancherla	Clinical/Surgical
~~	~~~	Dr Priscilla Leone	Clinical/Surgical
~~	~~~	Mr Bryan Singizi	Point of contact (1st)
~~	~~~	Ms Sian Gibson	Point of contact (1st)
~~	~~~	Ms Lauren Perkins	Point of contact (2nd)
~~	~~~	Ms Mandy Austin	Point of contact (2nd)
~~	~~~	Ms Elizabeth Dawson	Research Nurse
~~	~~~	Sister Tracey Camburn	Research Nurse
~~	~~~	Ms Valerie Ramsay	Trial Coordinator
~~	~~~	Mr Christian Barnett	Data Manager
UK	Cheltenham: Cheltenham General Hosp	Dr Jo Bowen	Site PI

INVESTIGATORS AND COLLABORATORS: SITE STAFF
Staff on site delegation logs

Country	Site	Names	Role
~~	~~~	Dr Audrey Cook	Clinical/Surgical
~~	~~~	Dr Chris Shimell	Clinical/Surgical
~~	~~~	Dr Colin Binks	Clinical/Surgical
~~	~~~	Dr Peter Jenkins	Clinical/Surgical
~~	~~~	Dr Roger Owen	Clinical/Surgical
~~	~~~	Dr Sai Jonnada	Clinical/Surgical
~~	~~~	Miss Sarah Beazer	Point of contact (1st)
~~	~~~	Mr Matthew Tan	Point of contact (1st)
~~	~~~	Mrs Julie Allen	Point of contact (1st)
~~	~~~	Mrs Kate Trigg-Hogarth	Point of contact (2nd)
~~	~~~	Miss Amy Skelton	Research Nurse
~~	~~~	Mrs Chris Ford	Research Nurse
~~	~~~	Mrs Elaine Pratten	Research Nurse
~~	~~~	Mrs Sue Wronski	Research Nurse
~~	~~~	Mrs Susan Anderson	Research Nurse
~~	~~~	Mrs Barbara Broomfield	Trial Coordinator
~~	~~~	Mrs Rachel Sayers	Trial Coordinator
~~	~~~	Ms Janet Forkes	Trial Coordinator
~~	~~~	Ms Rehana Bakawala	Trial Coordinator
~~	~~~	Mr Nigel Johnson	Data Manager
~~	~~~	Mrs Julia Hall	Data Manager
~~	~~~	Mrs Lin Crossley	Data Manager
~~	~~~	Ms Jennifer Healey-Mariano	Research Asst
~~	~~~	Miss Eleanor Andrews (nee Moore)	Administrator
UK	Chester: Countess of Chester Hosp	Dr Azman Ibrahim	Site PI
~~	~~~	Miss Elizabeth Gallimore	Point of contact (1st)
~~	~~~	Mrs Sue Green	Point of contact (1st)
~~	~~~	Ms Carys Jones	Point of contact (1st)
~~	~~~	Ms Jude Prince	Point of contact (1st)
~~	~~~	Ms Mary Aldous	Research Nurse
~~	~~~	Ms Denise Archer	Data Manager
~~	~~~	Ms Helen Eccleson	Data Manager
UK	Chesterfield: Chesterfield Royal Hosp	Dr Omar Din	Site PI
~~	~~~	Dr Peter Kirkbride	Clinical/Surgical

INVESTIGATORS AND COLLABORATORS: SITE STAFF

Staff on site delegation logs

Country	Site	Names	Role
~~	~~~	Ms Lucy Smith	Clinical/Surgical
~~	~~~	Ms Lesley Stevenson	Point of contact (1st)
~~	~~~	Ms Vittoria Sorice	Point of contact (1st)
~~	~~~	Mrs Nicky Ford	Point of contact (2nd)
~~	~~~	Mrs Melanie Chrystal	Research Nurse
~~	~~~	Ms Julie Toms	Research Nurse
~~	~~~	Sister Kim Wood	Research Nurse
~~	~~~	Ms Alison Redfearn	Trial Coordinator
~~	~~~	Miss Alexandra Firth	Data Manager
~~	~~~	Mr John Martindale	Data Manager
~~	~~~	Mrs Janine Smedley (nee McCabe)	Data Manager
UK	Colchester: Essex County Hosp	Dr Dakshinamoorthy Muthu Kumar	Site PI
~~	~~~	Dr Bruce Sizer	Clinical/Surgical
~~	~~~	Dr Devy Basu	Clinical/Surgical
~~	~~~	Dr Muthar Kumar	Clinical/Surgical
~~	~~~	Dr Priscilla Leone	Clinical/Surgical
~~	~~~	Mr Louies Mabelin	Point of contact (1st)
~~	~~~	Mrs Liz Hunting	Point of contact (1st)
~~	~~~	Ms Celine Driscoll	Point of contact (1st)
~~	~~~	Ms Lorna Dewar	Trial Coordinator
~~	~~~	Mrs Lucy Thorogood	Data Manager
~~	~~~	Ms Nyssa Barke	Administrator
UK	Cornwall: Royal Cornwall Hosp	Dr Alastair H Thomson	Site PI
~~	~~~	Dr Aaron Gould	Clinical/Surgical
~~	~~~	Dr Duncan Wheatley	Clinical/Surgical
~~	~~~	Miss Elizabeth Firth	Point of contact (1st)
~~	~~~	Mr Christopher Hocking	Point of contact (1st)
~~	~~~	Ms Anita Steele	Point of contact (1st)
~~	~~~	Ms Corrine Penhaligon	Point of contact (1st)
~~	~~~	Miss Alice Topps	Point of contact (2nd)
~~	~~~	Ms Catherine Pentecost	Point of contact (2nd)
~~	~~~	Ms Jane Broom	Point of contact (2nd)
~~	~~~	Ms Sarah Askew	Point of contact (2nd)
~~	~~~	Mrs Thea Barlow	Research Nurse

INVESTIGATORS AND COLLABORATORS: SITE STAFF
Staff on site delegation logs

Country	Site	Names	Role
~~	~~~	Mrs Thea Barlow	Research Nurse
~~	~~~	Ms Emma Duley	Research Nurse
~~	~~~	Ms Emma Kent	Research Nurse
~~	~~~	Dr Darren Beech	Trial Coordinator
~~	~~~	Miss Melissa Poultney	Data Manager
~~	~~~	Mr Luke Townley	Data Manager
~~	~~~	Mr William Pynsent	Data Manager
~~	~~~	Ms Kerena Partridge	Data Manager
~~	~~~	Mr John Madine	Research Asst
~~	~~~	Miss Louise Johns	Administrator
~~	~~~	Mrs Kay Pollard	Administrator
~~	~~~	Ms Johanna Skewes	Pharmacist
UK	Coventry: University Hosp Coventry & Warwickshire	Dr Jane Worlding	Site PI
~~	~~~	Dr Andrew Chan	Clinical/Surgical
~~	~~~	Dr Andrew Stockdale	Clinical/Surgical
~~	~~~	Dr Caroline Humber	Clinical/Surgical
~~	~~~	Dr Medy Tsalic	Clinical/Surgical
~~	~~~	Dr Yakhub Khan	Clinical/Surgical
~~	~~~	Manreet Thind	Point of contact (1st)
~~	~~~	Mr Albert Mislang	Point of contact (1st)
~~	~~~	Ms Kerry Geraghty	Point of contact (1st)
~~	~~~	Manju Sunny	Point of contact (2nd)
~~	~~~	Mrs Laura Stanley	Point of contact (2nd)
~~	~~~	Mrs Elaine Simmons	Research Nurse
~~	~~~	Mrs Lesley Hayward	Research Nurse
~~	~~~	Mrs Rosaleen Laverick	Research Nurse
~~	~~~	Ms Fiona Tranter	Research Nurse
~~	~~~	Ms Kay Sanders	Research Nurse
~~	~~~	Ms Su Ngwenya	Research Nurse
~~	~~~	Sister Judith Lake	Research Nurse
~~	~~~	Mr Pritpal Panesar	Trial Coordinator
~~	~~~	Ms Maggie Brown	Trial Coordinator
~~	~~~	Miss Emily Steventon	Data Manager
~~	~~~	Mrs Sue Elwell	Data Manager

INVESTIGATORS AND COLLABORATORS: SITE STAFF
Staff on site delegation logs

Country	Site	Names	Role
~~	~~~	Mrs Theresa Griffiths	Data Manager
~~	~~~	Miss Melanie Sinfield	Administrator
~~	~~~	Ms Gemma Mansell	Administrator
~~	~~~	Ms Marie Connor	MDT coordinator
UK	Crewe: Leighton Hosp	Dr Anna Tran	Site PI
~~	~~~	Dr James Wylie	Clinical/Surgical
~~	~~~	Dr John Logue	Clinical/Surgical
~~	~~~	Mr P Irwin	Clinical/Surgical
~~	~~~	Mr P Javle	Clinical/Surgical
~~	~~~	Miss Adele Hough	Point of contact (1st)
~~	~~~	Mrs Caroline Walker	Point of contact (1st)
~~	~~~	Ms Carolyn Mansfield	Point of contact (1st)
~~	~~~	Ms Joanne Hughes	Point of contact (1st)
~~	~~~	Ms Lesley Sumner	Point of contact (1st)
~~	~~~	Ms Rachel Smith	Point of contact (1st)
~~	~~~	Miss Annabel Tomlinson	Point of contact (2nd)
~~	~~~	Mrs Chris Hough	Research Nurse
~~	~~~	Ms Vanessa Adamson	Research Nurse
~~	~~~	Mrs Carole Bennion	Administrator
~~	~~~	Ms Jenny Butler-Barnes	Other
UK	Derby: Royal Derby Hosp	Dr Prantik Das	Site PI
~~	~~~	Dr Dakshinamoorthy Muthukumar	Clinical/Surgical
~~	~~~	Dr Prabir Chakraborti	Clinical/Surgical
~~	~~~	Mr Pugazhenthi Pattu	Clinical/Surgical
~~	~~~	Ms Dawn Ennis	Point of contact (1st)
~~	~~~	Ms Sue Marriott	Research Nurse
~~	~~~	Mr Colin Ward	Pharmacist
UK	Doncaster: Doncaster Royal Inf	Dr Carmel Pezaro	Site PI
~~	~~~	Dr Catherine Ferguson	Clinical/Surgical
~~	~~~	Dr Mymoona Alzouebi	Clinical/Surgical
~~	~~~	Ms Lucy Smith	Clinical/Surgical
~~	~~~	Mrs Nicola Wilkinson	Point of contact (1st)
~~	~~~	Ms Georgia Hooton	Point of contact (2nd)
~~	~~~	Miss Jennifer Taylor	Research Nurse

INVESTIGATORS AND COLLABORATORS: SITE STAFF

Staff on site delegation logs

Country	Site	Names	Role
~~	~~~	Mrs Amy Neal	Research Nurse
~~	~~~	Mrs Nicole Jeffcutt	Research Nurse
~~	~~~	Ms Barbara Burlace	Research Nurse
~~	~~~	Sister Kim Wood	Research Nurse
~~	~~~	Miss Alexandra Firth	Data Manager
~~	~~~	Mr John Martindale	Data Manager
~~	~~~	Mrs Janine Smedley (nee McCabe)	Data Manager
~~	~~~	Dr Virgil Sivoglo	Other
UK	Dorchester: Dorset County Hosp	Dr Benjamin Masters	Site PI
~~	~~~	Mr Naveed Afzal	Site PI
~~	~~~	Dr Perric Crellin	Clinical/Surgical
~~	~~~	Mr Andrew Cornaby	Clinical/Surgical
~~	~~~	Mr Stephen Andrews	Clinical/Surgical
~~	~~~	Mrs Josie Goodsell	Point of contact (1st)
~~	~~~	Ms Kate Taylor	Point of contact (1st)
~~	~~~	Mr Piet Bakker	Research Nurse
~~	~~~	Mr Simon Sharpe	Research Nurse
~~	~~~	Mrs Jackie Gibbins	Research Nurse
~~	~~~	Mrs Sally Love	Research Nurse
~~	~~~	Ms Beverley Anderson	Research Nurse
~~	~~~	Ms Sally Breakspear	Research Nurse
~~	~~~	Ms Sarah Horton	Research Nurse
~~	~~~	Ms Stephanie Jones	Research Nurse
~~	~~~	Ms Tracy Glen	Research Nurse
~~	~~~	Mr Andrew Gibbins	Data Manager
~~	~~~	Mr Andrew Rees	Data Manager
~~	~~~	Ms Suzy Wignall	Data Manager
UK	Dudley: Russells Hall Hosp	Dr Pek Keng-Koh	Site PI
~~	~~~	Dr Prakash Ramachandra	Clinical/Surgical
~~	~~~	Lesley Jones	Point of contact (1st)
~~	~~~	Sister Kath Harrow	Point of contact (1st)
~~	~~~	Ms Karen McGarry	Point of contact (2nd)
~~	~~~	Mrs Lucy Smith	Research Nurse
~~	~~~	Ms Karen Kanyi	Research Nurse

INVESTIGATORS AND COLLABORATORS: SITE STAFF
Staff on site delegation logs

Country	Site	Names	Role
~~	~~~	Ms Angela Watts	Other
UK	Eastbourne: Eastbourne District General Hosp	Dr Caroline Manetta	Site PI
~~	~~~	Dr Duncan Gilbert	Clinical/Surgical
~~	~~~	Dr Fiona McKinna	Clinical/Surgical
~~	~~~	Mrs Jo-Anne Taylor	Point of contact (1st)
~~	~~~	Ms Shelley Baumber	Point of contact (2nd)
~~	~~~	Mrs Kay Jones-Skipper	Research Nurse
~~	~~~	Ms Lauren McCrisken	Research Nurse
~~	~~~	Ms Amanda Williams	Trial Coordinator
~~	~~~	Ms Joanna Howard	Data Manager
UK	Edinburgh: Western General Hosp	Dr Duncan McLaren	Site PI
~~	~~~	Mr Param Mariappan	Clinical/Surgical
~~	~~~	Ms Susan Forman	Point of contact (1st)
~~	~~~	Ms Catherine Woods	Point of contact (2nd)
~~	~~~	Mrs Barbara Mayne	Research Nurse
~~	~~~	Ms Lisa Egan	Research Nurse
~~	~~~	Ms Kathleen Fiddes	Trial Coordinator
~~	~~~	Mr Brian Rogers	Data Manager
~~	~~~	Mr David Jeffrey	Data Manager
~~	~~~	Ms Alison Clark	Data Manager
~~	~~~	Ms Fiona Gardiner	Data Manager
~~	~~~	Ms Maria Clarke	Data Manager
~~	~~~	Dr Douglas Young	Other
UK	Exeter: Royal Devon & Exeter Hosp	Dr Rajaguru Srinivasan	Site PI
~~	~~~	Dr Victoria Ford	Site PI
~~	~~~	Dr Denise Sheehan	Clinical/Surgical
~~	~~~	Dr John McGrath	Clinical/Surgical
~~	~~~	Mr M Crundwell	Clinical/Surgical
~~	~~~	Mr Mark Stott	Clinical/Surgical
~~	~~~	Mrs Claire Webb	Point of contact (1st)
~~	~~~	Mrs Ingrid Seath	Point of contact (1st)
~~	~~~	Ms Ellen Matkins	Point of contact (1st)
~~	~~~	Ms Jane Piper	Point of contact (1st)
~~	~~~	Ms Kizzy Baines	Point of contact (1st)

INVESTIGATORS AND COLLABORATORS: SITE STAFF

Staff on site delegation logs

Country	Site	Names	Role
~~	~~~	Ms Alison Roantree	Research Nurse
~~	~~~	Ms Elizabeth Davey	Research Nurse
~~	~~~	Ms Shannon McGinley	Trial Coordinator
~~	~~~	Mr John Anderson	Data Manager
~~	~~~	Miss Theresa Lawless	Administrator
~~	~~~	Ms Katie Timmings	Radiographer
UK	Glasgow: Beatson West of Scotland Cancer Centre	Dr Azmat Sadozye	Site PI
~~	~~~	Dr Abdulla Al-hasso	Clinical/Surgical
~~	~~~	Dr David Dodds	Clinical/Surgical
~~	~~~	Dr Jan Wallace	Clinical/Surgical
~~	~~~	Dr Martin Russell	Clinical/Surgical
~~	~~~	Dr Norma Sidek	Clinical/Surgical
~~	~~~	Dr Rana Mahmood	Clinical/Surgical
~~	~~~	Dr Rob Jones	Clinical/Surgical
~~	~~~	Mrs Ailsa Griffen	Point of contact (1st)
~~	~~~	Niall Finnegan	Point of contact (1st)
~~	~~~	Ms Chloe Cowan	Research Nurse
~~	~~~	Ms Lorraine Barwell	Research Nurse
~~	~~~	Ms Antonia MacMillan	Trial Coordinator
~~	~~~	Ms Sian Shirley	Trial Coordinator
~~	~~~	Ms Claire Lawless	Data Manager
~~	~~~	Ms Elaine Allan	Administrator
UK	Glasgow: The New Victoria ACH (Ambulatory Care Hosp)	Dr Abdulla Al-hasso	Site PI
~~	~~~	Dr Jawaher Ansari	Clinical/Surgical
~~	~~~	Mr Naeem Akhtar	Clinical/Surgical
~~	~~~	Mrs Emma Moody	Point of contact (1st)
~~	~~~	Ms Louise Humpreys	Point of contact (1st)
~~	~~~	Mrs Karen Bell	Research Nurse
~~	~~~	Ms Claudia Turley	Research Nurse
~~	~~~	Ms Donna McWilliam	Research Nurse
~~	~~~	Ms Rebecca O'Neil	Research Nurse
~~	~~~	Ms Suzannah Peck	Research Nurse
UK	Guildford: Royal Surrey County Hosp	Dr Chee Goh	Site PI
~~	~~~	Dr Aruna Medisetti	Clinical/Surgical

INVESTIGATORS AND COLLABORATORS: SITE STAFF

Staff on site delegation logs

Country	Site	Names	Role
~~	~~~	Dr Jenny Nobes	Clinical/Surgical
~~	~~~	Dr Julian Money-Kyrle	Clinical/Surgical
~~	~~~	Dr Richard Shaffer	Clinical/Surgical
~~	~~~	Dr Robert Laing	Clinical/Surgical
~~	~~~	Dr Sara Khaksar	Clinical/Surgical
~~	~~~	Dr Teresa Guerrero-Urbano	Clinical/Surgical
~~	~~~	Mrs Jules Jones	Point of contact (1st)
~~	~~~	Ms Laura Matthews	Point of contact (1st)
~~	~~~	Ms Raidah Auladin	Point of contact (1st)
~~	~~~	Ms Sarah Stone	Point of contact (1st)
~~	~~~	Mrs Barbara Molony-Oates	Research Nurse
~~	~~~	Mrs Caterina Bissa	Research Nurse
~~	~~~	Mrs Jane Woods	Research Nurse
~~	~~~	Mrs Min Wu	Trial Coordinator
~~	~~~	Ms Daisy May Smale	Trial Coordinator
~~	~~~	Ms Frances Sidi	Administrator
~~	~~~	Miss Weronika Rabsztyn	Radiographer
~~	~~~	Mr Fabio Di Maria	Radiographer
~~	~~~	Mr Joshua Harding	Radiographer
~~	~~~	Mrs Annie Tindall	Radiographer
~~	~~~	Ms Ceri Jamieson	Radiographer
~~	~~~	Ms Marianne Dabbs	Radiographer
~~	~~~	Ms Stephanie Bird	Radiographer
UK	Harlow: Princess Alexandra Hosp (Harlow)	Dr Lucinda Melcher	Site PI
~~	~~~	Ms Amelia Daniel	Point of contact (1st)
~~	~~~	Ms Teresa Light	Point of contact (1st)
~~	~~~	Ms Lily Robinson	Point of contact (2nd)
~~	~~~	Ms Nikki Staines	Point of contact (2nd)
~~	~~~	Ms Amanda Lewis	Research Nurse
~~	~~~	Ms Joanne Kellaway	Research Nurse
~~	~~~	Ms Evelyn Holmes	Pharmacist
UK	Hereford: Hereford County Hosp	Dr Warren Grant	Site PI
~~	~~~	Dr Audrey Cook	Clinical/Surgical
~~	~~~	Mr Josh Follows	Point of contact (1st)

INVESTIGATORS AND COLLABORATORS: SITE STAFF

Staff on site delegation logs

Country	Site	Names	Role
~~	~~~	Mrs Janine Jones (Birch)	Point of contact (1st)
~~	~~~	Mrs Melanie Evans	Point of contact (1st)
~~	~~~	Miss Lisa King	Point of contact (2nd)
~~	~~~	Miss Sophie Cooper	Point of contact (2nd)
~~	~~~	Ms Sophie Myles	Point of contact (2nd)
~~	~~~	Miss Nicola Williamson	Research Nurse
~~	~~~	Miss Serrafina Carini	Research Nurse
~~	~~~	Mrs Susan Anderson	Research Nurse
~~	~~~	Ms Claire Hughes	Research Nurse
~~	~~~	Ms Lily Mercer	Research Nurse
~~	~~~	Ms Janet Forkes	Trial Coordinator
~~	~~~	Mrs Zara Roberts	Data Manager
~~	~~~	Mrs Laura Lees	Administrator
~~	~~~	Mr Andy Hedges	Pharmacist
UK	Herts: Mount Vernon Hosp	Dr Peter Ostler	Site PI
~~	~~~	Prof Peter Hoskin	Site PI
~~	~~~	Dr Jeanette Dickson	Clinical/Surgical
~~	~~~	Dr Nicola Anyamene	Clinical/Surgical
~~	~~~	Dr Robert Hughes	Clinical/Surgical
~~	~~~	Dr Roberto Alonzi	Clinical/Surgical
~~	~~~	Miss Lucy Collins	Point of contact (1st)
~~	~~~	Ms Justina Kailey	Point of contact (1st)
~~	~~~	Ms Justina Kailey	Point of contact (1st)
~~	~~~	Ms Lesley Mitchell	Point of contact (1st)
~~	~~~	Ms Sara Abbassi	Point of contact (2nd)
~~	~~~	Ms Jessica Milner	Research Nurse
~~	~~~	Ms Julia Bici	Research Nurse
~~	~~~	Miss Aamna Rashid	Trial Coordinator
~~	~~~	Ms Paulina Kowalewska	Trial Coordinator
~~	~~~	Ms Suzanne Jenkins	Trial Coordinator
~~	~~~	Miss Stephanie Stapleton	Data Manager
~~	~~~	Ms Sandra Garrido-Perez	Other
UK	Hull: Castle Hill Hosp	Mr Matthew Simms	Site PI
~~	~~~	Dr Faheem Bashir	Clinical/Surgical

INVESTIGATORS AND COLLABORATORS: SITE STAFF
Staff on site delegation logs

Country	Site	Names	Role
~~	~~~	Dr Sanjay Dixit	Clinical/Surgical
~~	~~~	Mrs Julie Rawlings	Point of contact (1st)
~~	~~~	Mrs Diane Clark	Point of contact (2nd)
~~	~~~	Mrs Linzi Bone	Research Nurse
~~	~~~	Ms Sarah Palmer	Research Nurse
~~	~~~	Mr Kristian Plowman	Data Manager
~~	~~~	Mrs Dawn Jones	Data Manager
~~	~~~	Mrs Rhian Horne	Pharmacist
UK	Inverness: Raigmore Hosp	Dr Neil McPhail	Site PI
~~	~~~	Dr Alison Nicholls	Clinical/Surgical
~~	~~~	Dr Aristoula Papakostidi	Clinical/Surgical
~~	~~~	Dr Carol Macgregor	Clinical/Surgical
~~	~~~	Dr David Whillis	Clinical/Surgical
~~	~~~	Dr Kay Kelly	Clinical/Surgical
~~	~~~	Mr Sudhir Borgaonkar	Clinical/Surgical
~~	~~~	Mrs Rachel Mackay	Point of contact (1st)
~~	~~~	Mr Sean Neville	Research Nurse
~~	~~~	Mrs Anglise Addison	Research Nurse
~~	~~~	Mrs Georgina Simpson	Research Nurse
~~	~~~	Mrs Sandra Brown	Research Nurse
~~	~~~	Ms Morag McNally	Research Nurse
~~	~~~	Mrs Alison Macdonald	Data Manager
~~	~~~	Mrs Seonaid Arnott	Data Manager
~~	~~~	Ms Anna Skene	Data Manager
~~	~~~	Ms Glenda Sinclair	Data Manager
~~	~~~	Ms Debbie Lister	MDT coordinator
~~	~~~	Mr Jude Madeleine	Pharmacist
~~	~~~	Ms Audrey Campbell	Pharmacist
~~	~~~	Ms Zoe Urquhart	Pharmacist
~~	~~~	Mrs Margaret Cormack	Radiographer
~~	~~~	Mrs Sheena Telfer	Radiographer
~~	~~~	Mrs Victoria Doughty	Radiographer
~~	~~~	Mr Steve Colligan	Other
UK	Ipswich: Ipswich Hosp	Dr Christopher Scrase	Site PI

INVESTIGATORS AND COLLABORATORS: SITE STAFF

Staff on site delegation logs

Country	Site	Names	Role
~~	~~~	Dr Ramachandran Venkitaraman	Clinical/Surgical
~~	~~~	Mr Paul Ridley	Point of contact (1st)
~~	~~~	Ms Susan Upson	Point of contact (1st)
~~	~~~	Mrs Amanda Ford	Radiographer
~~	~~~	Mrs Charlotte Etheridge	Other
UK	Kidderminster: Kidderminster General Hosp	Dr Lisa Capaldi	Site PI
~~	~~~	Dr Mark Churn	Clinical/Surgical
~~	~~~	Mrs Helen Tranter	Point of contact (1st)
~~	~~~	Mr Jacob Taylor	Point of contact (2nd)
~~	~~~	Mrs Julie Wollaston	Research Nurse
~~	~~~	Ms Helen Knott	Research Nurse
~~	~~~	Ms Sally Stringer (pr. Davis)	Research Nurse
~~	~~~	Ms Linda Higgins	Data Manager
UK	Larbert: Forth Valley Royal Hosp	Dr Norma Sidek	Site PI
~~	~~~	Dr Martin Russell	Clinical/Surgical
~~	~~~	Mr James Tweedle	Clinical/Surgical
~~	~~~	Mr Seamus Teahan	Clinical/Surgical
~~	~~~	Miss Stephanie Brogan (nee Roddie)	Point of contact (1st)
~~	~~~	Mrs Lynn Prentice	Point of contact (1st)
~~	~~~	Mrs Anne Todd	Point of contact (2nd)
~~	~~~	Mrs Lesley Symon	Point of contact (2nd)
~~	~~~	Mrs Sally Young	Research Nurse
~~	~~~	Ms Susan Erskine	MDT coordinator
~~	~~~	Mrs Maureen Hamill	Other
UK	Leeds: St James University Hosp (Leeds)	Dr David Bottomley	Site PI
~~	~~~	Dr Ann Henry	Clinical/Surgical
~~	~~~	Dr Anne Kiltie	Clinical/Surgical
~~	~~~	Dr Carmel Loughrey	Clinical/Surgical
~~	~~~	Dr Catherine Coyle	Clinical/Surgical
~~	~~~	Dr Ian Boon	Clinical/Surgical
~~	~~~	Mr Edmund Breckin	Point of contact (1st)
~~	~~~	Mrs Pam Shuttleworth	Point of contact (1st)
~~	~~~	Ms Beccy Smith	Point of contact (1st)
~~	~~~	Mrs Jude Clarke	Research Nurse

INVESTIGATORS AND COLLABORATORS: SITE STAFF

Staff on site delegation logs

Country	Site	Names	Role
~~	~~~	Ms Emily Davies	Research Nurse
~~	~~~	Ms Gemma Austin (nee Glover)	Research Nurse
~~	~~~	Ms Claire Pratt	Trial Coordinator
~~	~~~	Ms Fatima Murad	Trial Coordinator
~~	~~~	Mr James Goulding	Administrator
~~	~~~	Ms Eleanor Moore	Administrator
~~	~~~	Ms Gill Smith	MDT coordinator
UK	Leicester: Leicester Royal Inf	Dr Subramanian Vasanthan	Site PI
~~	~~~	Dr Christopher Kent	Clinical/Surgical
~~	~~~	Dr Lesley Speed	Clinical/Surgical
~~	~~~	Mr Leyshon Griffiths	Clinical/Surgical
~~	~~~	Mr Roger Kockelbergh	Clinical/Surgical
~~	~~~	Ms Janet Potterton	Point of contact (1st)
~~	~~~	Ms Sallyanne Christmas	Point of contact (2nd)
~~	~~~	Mrs Julia Walker	Research Nurse
~~	~~~	Ms Jill Cooke	Research Nurse
~~	~~~	Ms Amy Branson (née Dineen)	Research Asst
~~	~~~	Ms Amy King	Administrator
UK	Lincoln: Lincoln County Hosp	Dr Thiagarajan Sreenivasan	Site PI
~~	~~~	Dr Karin Baria	Clinical/Surgical
~~	~~~	Dr Miguel Panades	Clinical/Surgical
~~	~~~	Mr Ian Mark	Clinical/Surgical
~~	~~~	Mr Nazeer Dahar	Clinical/Surgical
~~	~~~	Mr Pallon Daruwala	Clinical/Surgical
~~	~~~	Mrs Olesya Francis	Point of contact (1st)
~~	~~~	Ms Kathryn Hoare	Point of contact (1st)
~~	~~~	Ms Rachel Newton	Point of contact (1st)
~~	~~~	Mr Andrew Sloan	Point of contact (2nd)
~~	~~~	Mr Simon Archer	Research Nurse
~~	~~~	Ms Claire Key	Research Nurse
~~	~~~	Ms Helen Ginnelly	Research Nurse
~~	~~~	Ms Sarah Coombs	Research Nurse
~~	~~~	Miss Samantha Bateman	Data Manager
~~	~~~	Mrs Jane Hall	Radiographer

INVESTIGATORS AND COLLABORATORS: SITE STAFF
Staff on site delegation logs

Country	Site	Names	Role
~~	~~~	Mrs Maryanne Okubanjo	Radiographer
~~	~~~	Ms Carol Lockwood	Radiographer
~~	~~~	Ms Amy Cunningham	Other
UK	Liverpool: Royal Liverpool University Hosp	Dr Zafar Malik	Site PI
~~	~~~	Dr Peter Robson	Clinical/Surgical
~~	~~~	Miss Lynsey Dean	Point of contact (1st)
~~	~~~	Mr Thomas Rogers	Point of contact (1st)
~~	~~~	Miss Katy Treherne	Research Nurse
~~	~~~	Ms Nicola Bermingham	Research Nurse
~~	~~~	Ms Pauline Pilkington	Research Nurse
~~	~~~	Ms Heather Rogers	Other
UK	London: Charing Cross Hosp	Dr Alison Falconer	Site PI
~~	~~~	Dr May Stancliffe	Clinical/Surgical
~~	~~~	Dr Sathish Harinarayanan	Clinical/Surgical
~~	~~~	Dr Simon Stewart	Clinical/Surgical
~~	~~~	Mr Ross Dalton-Short	Point of contact (1st)
~~	~~~	Mr Steve Edwards	Point of contact (1st)
~~	~~~	Ms Sarah Rezkallah	Point of contact (1st)
~~	~~~	Ms Sue McInerney	Point of contact (2nd)
~~	~~~	Nebah Hassan	Point of contact (2nd)
~~	~~~	Ms Anne-Maree Thoi	Research Nurse
~~	~~~	Ms Ibiyemi Sadare (Olaleye)	Research Nurse
~~	~~~	Ms Amy Ford	Trial Coordinator
~~	~~~	Ms Bindu Chikkamuniyappa	Trial Coordinator
~~	~~~	Ms Gillian Hornzee	Trial Coordinator
~~	~~~	Miss Anna Westrop	Administrator
~~	~~~	Mr Bhavesh Pratap	Administrator
~~	~~~	Ms Sophia Magwaro	Radiographer
~~	~~~	Ms Amie Bourke	Other
UK	London: Croydon University Hosp	Mr Babbin John	Site PI
~~	~~~	Dr Adham Hijab	Clinical/Surgical
~~	~~~	Dr Alison Tree	Clinical/Surgical
~~	~~~	Dr Miguel Ferreira	Clinical/Surgical
~~	~~~	Dr Priyanka Patel	Clinical/Surgical

INVESTIGATORS AND COLLABORATORS: SITE STAFF
Staff on site delegation logs

Country	Site	Names	Role
~~	~~~	Dr Robert Huddart	Clinical/Surgical
~~	~~~	Mr Matthew Perry	Clinical/Surgical
~~	~~~	Mr Nasr Arsanious	Clinical/Surgical
~~	~~~	Ms Claire Crowley	Clinical/Surgical
~~	~~~	Ms Ibiyemi Sadare (Olaleye)	Point of contact (1st)
~~	~~~	Mrs Yvonne Campbell	Point of contact (2nd)
~~	~~~	Ms Shaki Balogun	Point of contact (2nd)
~~	~~~	Mrs Ann Payne	Research Nurse
~~	~~~	Ms Anne Haldeos	Research Nurse
~~	~~~	Ms Cheryl Batish	Research Nurse
~~	~~~	Ms Christine Springall	Research Nurse
~~	~~~	Ms Jane Thompson	Research Nurse
~~	~~~	Sister Jane Thomson	Research Nurse
~~	~~~	Mrs Maria Serra	Research Asst
~~	~~~	Ms Jackie Pach	Administrator
~~	~~~	Ms Sheefa Ahamadali	MDT coordinator
~~	~~~	Dr Andriana Michaelidou	Other
~~	~~~	Mr Mohammed Nawrozzadeh	Other
~~	~~~	Ms Emma Dunne	Other
UK	London: Guy's Hosp (London)	Dr Stephen Morris	Site PI
~~	~~~	Dr Kannon Nathan	Clinical/Surgical
~~	~~~	Dr Ronald Beaney	Clinical/Surgical
~~	~~~	Dr Sarah Harris	Clinical/Surgical
~~	~~~	Dr Simon Hughes	Clinical/Surgical
~~	~~~	Dr Teresa Guerrero-Urbano	Clinical/Surgical
~~	~~~	Ms Gabriella Assante	Point of contact (1st)
~~	~~~	Ms Jessica Rashid	Point of contact (1st)
~~	~~~	Ms Katie Jones	Point of contact (1st)
~~	~~~	Ms Viviana Aya	Point of contact (1st)
~~	~~~	Miss Lorna Bower	Research Nurse
~~	~~~	Mr Brendan Hore	Trial Coordinator
~~	~~~	Mr Greg Kuenzig	Trial Coordinator
~~	~~~	Mrs Vesna Hogan	Trial Coordinator
~~	~~~	Ms Gabriella Assante	Trial Coordinator

INVESTIGATORS AND COLLABORATORS: SITE STAFF

Staff on site delegation logs

Country	Site	Names	Role
~~	~~~	Ms Isabel Grau	Trial Coordinator
~~	~~~	Mr Hussain Gordon	Research Asst
~~	~~~	Miss Lisa-Jane Conway	Radiographer
~~	~~~	Mr Philip Reynolds	Radiographer
~~	~~~	Ms Sally Donaghey	Radiographer
~~	~~~	Bali Rooprai	Other
~~	~~~	Mrs Dawn Nunney	Other
UK	London: Hammersmith Hosp	Dr Stephen Mangar	Site PI
~~	~~~	Dr Simon Stewart	Clinical/Surgical
~~	~~~	Mr Ross Dalton-Short	Point of contact (1st)
~~	~~~	Mr Steve Edwards	Point of contact (1st)
~~	~~~	Ms Sarah Rezkallah	Point of contact (1st)
~~	~~~	Ms Bindu Chikkamuniyappa	Point of contact (2nd)
~~	~~~	Nebah Hassan	Point of contact (2nd)
~~	~~~	Ms Anne-Maree Thoi	Research Nurse
~~	~~~	Miss Anna Westrop	Administrator
~~	~~~	Ms Sophia Magwaro	Radiographer
UK	London: North Middlesex Hosp	Dr Nishi Gupta	Site PI
~~	~~~	Dr Farhad Neave	Clinical/Surgical
~~	~~~	Dr Jackie Newby	Clinical/Surgical
~~	~~~	Dr Lucinda Melcher	Clinical/Surgical
~~	~~~	Dr Stephen Karp	Clinical/Surgical
~~	~~~	Miss Sagal Kullane	Point of contact (1st)
~~	~~~	Ms Judy Hill	Research Nurse
~~	~~~	Miss Chloe Van Someren	Data Manager
~~	~~~	Mr Tom Caumont	Data Manager
~~	~~~	Ms Hanna Azirar	Data Manager
~~	~~~	Ms Tina Macavoy	MDT coordinator
UK	London: Royal Free Hosp	Dr Sarah Needleman	Site PI
~~	~~~	Dr Katherine Pigott	Clinical/Surgical
~~	~~~	Dr Maria Vilarino-Varela	Clinical/Surgical
~~	~~~	Dr Nicola Rosenfelder	Clinical/Surgical
~~	~~~	Ms Alexandra Gore	Point of contact (1st)
~~	~~~	Ms Claire Jarvis	Point of contact (1st)

INVESTIGATORS AND COLLABORATORS: SITE STAFF
Staff on site delegation logs

Country	Site	Names	Role
~~	~~~	Ms Hannah Powell	Point of contact (1st)
~~	~~~	Mrs Kaliyanee Ramtohul	Research Nurse
~~	~~~	Ms Angela McCadden	Research Nurse
~~	~~~	Ms Juniebel Cooke	Research Nurse
~~	~~~	Ms Lynda Annan	Research Nurse
~~	~~~	Ms Sara Fawcitt	Research Nurse
~~	~~~	Ms Sylvia Grieve	Research Nurse
~~	~~~	Miss Emma Douch	Trial Coordinator
~~	~~~	Ms Naomi Anderson	Data Manager
UK	London: Royal Marsden Hosp (London)	Dr Vincent Khoo	Site PI
~~	~~~	Dr Alison Tree	Clinical/Surgical
~~	~~~	Dr Liam Welsh	Clinical/Surgical
~~	~~~	Dr Nicholas Van As	Clinical/Surgical
~~	~~~	Miss Holly Hogan	Point of contact (1st)
~~	~~~	Miss Jennyfa Ali	Point of contact (1st)
~~	~~~	Mr Bernard Siu	Point of contact (1st)
~~	~~~	Mrs Sijy Pillai	Point of contact (1st)
~~	~~~	Ms Giulia Carlino	Point of contact (1st)
~~	~~~	Mr Joseph Montebello	Point of contact (2nd)
~~	~~~	Miss Vijitha Vijayakumar	Research Nurse
~~	~~~	Mr Trevor Bott	Research Nurse
~~	~~~	Mrs Debra Townsend-Thorn	Research Nurse
~~	~~~	Mrs Ruth Stafferton	Research Nurse
~~	~~~	Miss Hanna Bryant	Trial Coordinator
~~	~~~	Mr Emmanuel Brown	Trial Coordinator
~~	~~~	Ms Cordelia Grant	Trial Coordinator
~~	~~~	Ms Laillah-Crystal Banda	Trial Coordinator
~~	~~~	Miss Maryam Ali	Administrator
~~	~~~	Mr Chintan Mojidra	Administrator
~~	~~~	Mrs Sarah Storrs	Administrator
~~	~~~	Mr Matthew Olabanji	Other
~~	~~~	Ms Jennifer Morrison	Other
UK	London: Royal Marsden Hosp (Sutton)	Dr Chris Parker	Site PI
~~	~~~	Dr Ramachandran Venkitaraman	Clinical/Surgical

INVESTIGATORS AND COLLABORATORS: SITE STAFF

Staff on site delegation logs

Country	Site	Names	Role
~~	~~~	Dr Robert Huddart	Clinical/Surgical
~~	~~~	Prof Alan Horwich	Clinical/Surgical
~~	~~~	Prof David Dearnaley	Clinical/Surgical
~~	~~~	Mrs Annie Gao	Point of contact (1st)
~~	~~~	Ms Louise Murphy	Point of contact (1st)
~~	~~~	Mr John Marshall	Trial Coordinator
~~	~~~	Ms Val Lewington	Trial Coordinator
UK	London: St Bartholomews Hosp (London)	Dr Paula Wells	Site PI
~~	~~~	Dr Alexandre Kaliski	Clinical/Surgical
~~	~~~	Dr Karen Tipples	Clinical/Surgical
~~	~~~	Mrs Resmi Jayachandran	Point of contact (1st)
~~	~~~	Ms Olivia Bolton	Point of contact (1st)
~~	~~~	Mr Paul Hillman	Research Nurse
~~	~~~	Ms Janet Kiff	Research Nurse
~~	~~~	Mr Fatjon Dekaj	Trial Coordinator
~~	~~~	Mr Alastair Nicholson	Data Manager
~~	~~~	Mr Jude Nixon	Data Manager
~~	~~~	Ms Janet Oladimeji	Data Manager
~~	~~~	Ms Nanette Bech-Nielsen	Research Asst
~~	~~~	Mr Oscar Riches	Radiographer
~~	~~~	Mrs Samantha Chetiyawardana	Radiographer
~~	~~~	Dr Dan Smith	Other
~~	~~~	Dr John Conibear	Other
~~	~~~	Dr Kirsty Beaton	Other
UK	London: St Georges Hosp (London)	Mr Rami Issa	Site PI
~~	~~~	Dr Vincent Khoo	Clinical/Surgical
~~	~~~	Mr Chris Anderson	Clinical/Surgical
~~	~~~	Mr Matthew Perry	Clinical/Surgical
~~	~~~	Miss Claire Gilmartin	Point of contact (1st)
~~	~~~	Miss Serena Dover	Point of contact (1st)
~~	~~~	Mr Juel Tuazon	Point of contact (1st)
~~	~~~	Ms Sophie Golden	Point of contact (1st)
~~	~~~	Miss Jesusa Toledo	Research Nurse
~~	~~~	Mr Robert Varro	Research Nurse

INVESTIGATORS AND COLLABORATORS: SITE STAFF
Staff on site delegation logs

Country	Site	Names	Role
~~	~~~	Ms Helen Tighe	Research Nurse
~~	~~~	Ms Jane Gregg	Research Nurse
~~	~~~	Mr Mark Quarrell	Trial Coordinator
~~	~~~	Ms Chandni Patel	Trial Coordinator
~~	~~~	Ms Deirdre Daly	Data Manager
~~	~~~	Miss Titilayo Oni	Administrator
~~	~~~	Ms Debbie Rolfe	Pharmacist
UK	London: St Marys Hosp (London)	Dr Simon Stewart	Site PI
~~	~~~	Mr Mark Caballes	Point of contact (1st)
~~	~~~	Mrs Manisha Joshi	Point of contact (1st)
~~	~~~	Ms Emily Russell	Point of contact (1st)
~~	~~~	Ms Stephanie Ivie	Point of contact (1st)
~~	~~~	Ms Anne-Maree Thoi	Research Nurse
~~	~~~	Mr Farhan Naim	Trial Coordinator
~~	~~~	Ms Byiravey Pathmanathan	Trial Coordinator
~~	~~~	Ms Gillian Hornzee	Trial Coordinator
~~	~~~	Ms Joy Liau	Trial Coordinator
~~	~~~	Mr Vikram Bohra	Data Manager
~~	~~~	Ms Laura Custins	Data Manager
~~	~~~	Miss Anna Westrop	Administrator
~~	~~~	Mr Anup Patel	Administrator
~~	~~~	Ms Anna Tippins	MDT coordinator
~~	~~~	Ms Sophia Magwaro	Radiographer
UK	London: University College Hosp	Dr Heather Payne	Site PI
~~	~~~	Dr Reena Davda	Site PI
~~	~~~	Dr Ajay Aggarwal	Clinical/Surgical
~~	~~~	Dr Anita Mitra	Clinical/Surgical
~~	~~~	Dr David Woolf	Clinical/Surgical
~~	~~~	Dr Jonathan Teh	Clinical/Surgical
~~	~~~	Dr Julia Hall	Clinical/Surgical
~~	~~~	Dr Rachel Khong	Clinical/Surgical
~~	~~~	Mr Thomas Amoaten	Point of contact (1st)
~~	~~~	Ms Nicole Bonsu	Point of contact (1st)
~~	~~~	Ms Patricia Danaswamy	Point of contact (1st)

INVESTIGATORS AND COLLABORATORS: SITE STAFF

Staff on site delegation logs

Country	Site	Names	Role
~~	~~~	Ms Suzy Lowi	Point of contact (1st)
~~	~~~	Ms Didem Agdiran	Point of contact (2nd)
~~	~~~	Ms Helene Zilkha	Point of contact (2nd)
~~	~~~	Ms Samantha Whinn	Point of contact (2nd)
~~	~~~	Ms Annelies Gillesen	Research Nurse
~~	~~~	Mr Richard Merrick	Trial Coordinator
~~	~~~	Mrs Roshni Goel	Data Manager
~~	~~~	Ms Natasha Aslam	Data Manager
~~	~~~	Ms Zainab Butt	Radiographer
UK	London: Whipps Cross University Hosp	Mr James Green	Site PI
~~	~~~	Dr David Woolf	Clinical/Surgical
~~	~~~	Dr Paula Wells	Clinical/Surgical
~~	~~~	Mr John Hines	Clinical/Surgical
~~	~~~	Mr John Peters	Clinical/Surgical
~~	~~~	Mr Simon Holden	Clinical/Surgical
~~	~~~	Mr Timothy Philp	Clinical/Surgical
~~	~~~	Mrs Resmi Jayachandran	Point of contact (1st)
~~	~~~	Ms Olivia Bolton	Point of contact (1st)
~~	~~~	Miss Memory Kazingizi	Research Nurse
~~	~~~	Mr John O'Neill	Research Nurse
~~	~~~	Mr Paul Hillman	Research Nurse
~~	~~~	Mr Thompson Olaoni	Research Nurse
~~	~~~	Ms Emma Foster	Research Nurse
~~	~~~	Ms Linda Dawson-Athey	Research Nurse
~~	~~~	Ms Patricia Danaswamy	Research Nurse
~~	~~~	Ms Sadaf Zaidi	Trial Coordinator
~~	~~~	Ms Nanette Bech-Nielsen	Research Asst
~~	~~~	Mr Andrew Gillian	Pharmacist
UK	Macclesfield: Macclesfield District General Hosp	Mr Richard Brough	Site PI
~~	~~~	Mr Adebanji Adeyoju	Clinical/Surgical
~~	~~~	Mr Gerald Collins	Clinical/Surgical
~~	~~~	Mr Stephen CW Brown	Clinical/Surgical
~~	~~~	Mr Waheed Zafar	Clinical/Surgical
~~	~~~	Ms Magda Kujawa	Clinical/Surgical

INVESTIGATORS AND COLLABORATORS: SITE STAFF
Staff on site delegation logs

Country	Site	Names	Role
~~	~~~	Mrs Pippa Hill	Point of contact (1st)
~~	~~~	Mrs Victoria Adinkra	Research Nurse
~~	~~~	Ms Barbara Townley	Research Nurse
~~	~~~	Mr Iain Woodhouse	Trial Coordinator
~~	~~~	Ms Marilyn McCurrie	Administrator
~~	~~~	Ms Lisa Hardstaff	Other
UK	Maidstone: Maidstone Hosp	Dr Henry Taylor	Site PI
~~	~~~	Dr Carys Thomas	Clinical/Surgical
~~	~~~	Dr Christos Mikropoulos	Clinical/Surgical
~~	~~~	Mr John Donohue	Clinical/Surgical
~~	~~~	Dr Kathryn Lees	Clinical/Surgical
~~	~~~	Dr Patryk Brulinski	Clinical/Surgical
~~	~~~	Dr Sharon Beesley	Clinical/Surgical
~~	~~~	Mrs Alison Davison	Point of contact (1st)
~~	~~~	Ms Verity Roberts	Point of contact (1st)
~~	~~~	Mrs Ann Phillips	Research Nurse
~~	~~~	Mrs Jane Murray	Research Nurse
~~	~~~	Ms Barbara Mercier	Research Nurse
~~	~~~	Ms Carmel Jope	Research Nurse
~~	~~~	Ms Lisa Tribe	Research Nurse
~~	~~~	Ms Sarah Martins	Research Nurse
~~	~~~	Ms Vivienne Breen	Research Nurse
~~	~~~	Ms Yvonne Lines	Trial Coordinator
~~	~~~	Mr Gavin Fossey	Administrator
~~	~~~	Ms Clare Calvert	Administrator
~~	~~~	Mr Ian Pamphlett	Pharmacist
~~	~~~	Dr Pauline Wood	Radiographer
~~	~~~	Miss Katy Taylor	Radiographer
~~	~~~	Mr Innocent Neshiri	Radiographer
~~	~~~	Mrs Heather Dias	Radiographer
~~	~~~	Ms Jodie Hotine	Radiographer
~~	~~~	Ms Pavnish Rai	Radiographer
~~	~~~	Ms Sarah Rezkallah	Radiographer
~~	~~~	Mr Ifan Jones	Other

INVESTIGATORS AND COLLABORATORS: SITE STAFF

Staff on site delegation logs

Country	Site	Names	Role
~~	~~~	Ms Su Burrage	Other
UK	Manchester: Christie Hosp	Dr John Logue	Site PI
~~	~~~	Dr Ananya Choudhury	Clinical/Surgical
~~	~~~	Dr Jacqueline Livsey	Clinical/Surgical
~~	~~~	Dr James Wylie	Clinical/Surgical
~~	~~~	Dr Richard Cowan	Clinical/Surgical
~~	~~~	Dr Ruth Conroy	Clinical/Surgical
~~	~~~	Dr Tony Elliott	Clinical/Surgical
~~	~~~	Miss Amber Hart	Point of contact (1st)
~~	~~~	Ms Roonak Nazari	Point of contact (1st)
~~	~~~	Ms Sarah-Ellen Smith	Point of contact (1st)
~~	~~~	Ms Lucy Worsley	Point of contact (2nd)
~~	~~~	Miss Kate O'Connor	Research Nurse
~~	~~~	Mr Damian McCaul	Research Nurse
~~	~~~	Ms Carol Newbery	Research Nurse
~~	~~~	Ms Catherine Redshaw	Research Nurse
~~	~~~	Sister Jackie O'Dwyer	Research Nurse
~~	~~~	Sister Viv Thomas	Research Nurse
~~	~~~	Miss Holly White	Trial Coordinator
~~	~~~	Miss Laura Flanagan	Trial Coordinator
~~	~~~	Miss Maria Petsa	Trial Coordinator
~~	~~~	Miss Willemijn Spoor	Trial Coordinator
~~	~~~	Mr Adrian Fallaize	Trial Coordinator
~~	~~~	Mr Ekugbe Onoge	Trial Coordinator
~~	~~~	Mr Ian Duncan	Trial Coordinator
~~	~~~	Ms Joanne Oliver	Trial Coordinator
~~	~~~	Ms Viviana Carpio	Trial Coordinator
~~	~~~	Miss Sarah Green	Data Manager
~~	~~~	Mr Ian Bottomley	Data Manager
~~	~~~	Miss Kim Fair	Administrator
~~	~~~	Mrs Sue Davison	Administrator
~~	~~~	Mr Jonathan Buchan	MDT coordinator
~~	~~~	Ms Catherine Harris	Other
~~	~~~	Ms Cathryn Jones	Other

INVESTIGATORS AND COLLABORATORS: SITE STAFF

Staff on site delegation logs

Country	Site	Names	Role
UK	Manchester: Withington Hosp	Mr Vijay Sangar	Site PI
~~	~~~	Dr James Wylie	Clinical/Surgical
~~	~~~	Mr Vijay Ramani	Clinical/Surgical
~~	~~~	Ms Heena Mistry	Point of contact (1st)
~~	~~~	Ms Linda Bailey	Point of contact (1st)
~~	~~~	Ms Lindsay Piper	Point of contact (1st)
~~	~~~	Ms Molly Bennett	Point of contact (1st)
~~	~~~	Ms Kathryn Slevin	Point of contact (2nd)
~~	~~~	Miss Anna Gipson	Research Nurse
~~	~~~	Miss Fiona McCarth	Research Nurse
~~	~~~	Mrs Helen Haydock	Research Nurse
~~	~~~	Ms Fiona McCartin	Research Nurse
~~	~~~	Ms Kathryn Fellows	Research Nurse
~~	~~~	Ms Sarah Liptrott	Research Nurse
~~	~~~	Ms Thobekile Mthethwa	Research Nurse
~~	~~~	Ms Annie Duffy	Trial Coordinator
~~	~~~	Ms Sarah Sahin	Trial Coordinator
~~	~~~	Ms Rebecca Corless	Data Manager
~~	~~~	Ms Lillian Partington	Pharmacist
~~	~~~	Dr Jonathan Tuck	Other
~~	~~~	Dr WF Knox	Other
UK	Middlesbrough: James Cook University Hosp	Dr Clive Peedell	Site PI
~~	~~~	Dr David Wilson	Clinical/Surgical
~~	~~~	Dr Devadasan Shakespeare	Clinical/Surgical
~~	~~~	Dr Hans Van der Voet	Clinical/Surgical
~~	~~~	Dr John Hardman	Clinical/Surgical
~~	~~~	Dr Julia McBride	Clinical/Surgical
~~	~~~	Mr David Chadwick	Clinical/Surgical
~~	~~~	Ms Rita Mohan	Point of contact (1st)
~~	~~~	Mr Keith Harland	Point of contact (2nd)
~~	~~~	Mrs Alison Barnes	Research Nurse
~~	~~~	Mrs Carol Long	Research Nurse
~~	~~~	Ms Lorraine Atkinson	Research Nurse
~~	~~~	Sister Patricia McClurey	Research Nurse

INVESTIGATORS AND COLLABORATORS: SITE STAFF
Staff on site delegation logs

Country	Site	Names	Role
~~	~~~	Mrs Alison Chilvers	Trial Coordinator
~~	~~~	Ms Alison Chilvers	Trial Coordinator
~~	~~~	Ms Sarah McAuliffe	Trial Coordinator
~~	~~~	Ms Lynne Naylor	Administrator
~~	~~~	Ms Sarah Barnbrooke	MDT coordinator
~~	~~~	Mrs Paula Robson	Radiographer
~~	~~~	Ms Emma Thompson	Radiographer
~~	~~~	Ms Caroline Brownless	Other
UK	Newcastle: Freeman Hosp	Dr Ian Pedley	Site PI
~~	~~~	Dr John Frew	Clinical/Surgical
~~	~~~	Dr Rhona McMenemin	Clinical/Surgical
~~	~~~	Dr Trevor Roberts	Clinical/Surgical
~~	~~~	Miss Janine Tate	Point of contact (1st)
~~	~~~	Mr Jonathan Stoddart	Point of contact (1st)
~~	~~~	Ms Jayashree Walker	Trial Coordinator
~~	~~~	Miss Danielle Riseborough	Data Manager
~~	~~~	Miss Katy Lambert	Data Manager
~~	~~~	Miss Emma Stockley	Radiographer
~~	~~~	Mr Chris Barron	Radiographer
~~	~~~	Ms Bridget Workman	Other
UK	Newport: Royal Gwent Hosp	Mr Jim Wilson	Site PI
~~	~~~	Mr Adam Carter	Clinical/Surgical
~~	~~~	Mr Adam Cox	Clinical/Surgical
~~	~~~	Mr Syhed Rahman	Clinical/Surgical
~~	~~~	Miss Maxine Nash	Point of contact (1st)
~~	~~~	Mr Simon Hodge	Research Nurse
~~	~~~	Mrs Debra Barnett	Research Nurse
~~	~~~	Ms Julie Simpson	Research Nurse
~~	~~~	Ms Karen Wild	Research Nurse
~~	~~~	Ms Rachel Williams	Research Nurse
~~	~~~	Ms S Kearney	Research Nurse
~~	~~~	Ms Alison Davey	Trial Coordinator
~~	~~~	Ms Janet Marty	Trial Coordinator
~~	~~~	Ms Kirstin Davies	Trial Coordinator

INVESTIGATORS AND COLLABORATORS: SITE STAFF
Staff on site delegation logs

Country	Site	Names	Role
~~	~~~	Ms Lisa Gilby	Trial Coordinator
~~	~~~	Mrs Jayne Richards	Data Manager
~~	~~~	Mrs Paula Webb	Data Manager
~~	~~~	Ms Helene Lavender	Data Manager
~~	~~~	Ms Elaine Wall	Administrator
UK	Northampton: Northampton General Hosp	Dr Jenny Branagan	Site PI
~~	~~~	Dr Christine Elwell	Clinical/Surgical
~~	~~~	Dr Dorai Ramanathan	Clinical/Surgical
~~	~~~	Dr Philip Camilleri	Clinical/Surgical
~~	~~~	Mrs Ruby Goyena	Point of contact (1st)
~~	~~~	Ms Maxine Foo	Point of contact (1st)
~~	~~~	Ms Rachel Tighe (nee Bussey)	Point of contact (1st)
~~	~~~	Jane O'Callaghan	Point of contact (2nd)
~~	~~~	Ms Andrea Jones	Point of contact (2nd)
~~	~~~	Mrs Katherine McGrath	Research Nurse
~~	~~~	Ms Gillian Dell	Research Nurse
~~	~~~	Ms Bronwen Thominson	Administrator
~~	~~~	Mrs Elizabeth Tee	Other
~~	~~~	Ms Hazel McBain	Other
UK	Norwich: Norfolk & Norwich University Hosp	Dr Rob Wade	Site PI
~~	~~~	Dr Jenny Nobes	Clinical/Surgical
~~	~~~	Dr Joe Ostrowski	Clinical/Surgical
~~	~~~	Mr Guarav Kapur	Clinical/Surgical
~~	~~~	Mrs Adele Cooper	Clinical/Surgical
~~	~~~	Mrs Suzanne Walker	Clinical/Surgical
~~	~~~	Ms Karen Heasley	Clinical/Surgical
~~	~~~	Ms Cheryl Websdale	Point of contact (1st)
~~	~~~	Ms Sara Callam	Point of contact (1st)
~~	~~~	Ms Sarah Turner	Point of contact (1st)
~~	~~~	Ms Denise Archer	Point of contact (2nd)
~~	~~~	Dr Jane Beety	Trial Coordinator
~~	~~~	Mrs Joanna White	Trial Coordinator
~~	~~~	Mrs Sharon Walton	Trial Coordinator
~~	~~~	Ms Bridget Shobrook	Trial Coordinator

INVESTIGATORS AND COLLABORATORS: SITE STAFF
Staff on site delegation logs

Country	Site	Names	Role
~~	~~~	Ms Helen Darby	Trial Coordinator
~~	~~~	Mr Mark Bloomfield	Data Manager
~~	~~~	Mr Richard Birch	Data Manager
~~	~~~	Mrs Katrien Oosterom	Data Manager
~~	~~~	Miss Clare London	Pharmacist
~~	~~~	Miss Gail Healey	Pharmacist
UK	Nottingham: Nottingham University Hosps, City Campus	Dr Georgina Walker	Site PI
~~	~~~	Dr Ian Sayers	Clinical/Surgical
~~	~~~	Dr Jamie Mills	Clinical/Surgical
~~	~~~	Dr Santhanam Sundar	Clinical/Surgical
~~	~~~	Miss Leanne Alder	Point of contact (1st)
~~	~~~	Ms Hazel Marley	Point of contact (1st)
~~	~~~	Ms Cody Jevons	Point of contact (2nd)
~~	~~~	Ms Rachael Chivers	Point of contact (2nd)
~~	~~~	Miss Lucy Howard	Research Nurse
~~	~~~	Ms Hanna Purves	Research Nurse
~~	~~~	Mrs Catherine Wood	Administrator
~~	~~~	Mrs Carol Gooch	Radiographer
~~	~~~	Miss Camille Hutchinson	Other
~~	~~~	Mr Daniel Kumar	Other
~~	~~~	Mr Jacob Szolin-Jones	Other
UK	Oldham: Royal Oldham Hosp	Dr Jacqueline Livsey	Site PI
~~	~~~	Dr Ruth Conroy	Site PI
~~	~~~	Dr Ananya Choudhury	Clinical/Surgical
~~	~~~	Ms Lyndsay Scarratt	Point of contact (1st)
~~	~~~	Ms Dawn Johnstone	Point of contact (2nd)
~~	~~~	Ms Joanne Johnson	Point of contact (2nd)
~~	~~~	Mr Richard Jones	Research Nurse
~~	~~~	Ms Wendy Cook	Research Nurse
~~	~~~	Mrs Leena Mistry	Trial Coordinator
~~	~~~	Ms Lisa Gill	Trial Coordinator
UK	Oxford: Churchill Hosp	Dr Philip Camilleri	Site PI
~~	~~~	Dr Ahmad Sabbagh	Clinical/Surgical
~~	~~~	Dr Ami Sabharwal	Clinical/Surgical

INVESTIGATORS AND COLLABORATORS: SITE STAFF
Staff on site delegation logs

Country	Site	Names	Role
~~	~~~	Dr Anne Kiltie	Clinical/Surgical
~~	~~~	Dr David J Cole	Clinical/Surgical
~~	~~~	Dr Gagan Bhatnagar	Clinical/Surgical
~~	~~~	Dr Gerard Andrade	Clinical/Surgical
~~	~~~	Dr Hiba Al-Chamali	Clinical/Surgical
~~	~~~	Dr Katherine Hyde	Clinical/Surgical
~~	~~~	Dr Michael Skwarski	Clinical/Surgical
~~	~~~	Dr Niki Panakis	Clinical/Surgical
~~	~~~	Dr Raj Jampana	Clinical/Surgical
~~	~~~	Dr Rob Owens	Clinical/Surgical
~~	~~~	Dr Robert Stuart	Clinical/Surgical
~~	~~~	Mr Daniel Ajzensztejn	Clinical/Surgical
~~	~~~	Mr Simon Brewster	Clinical/Surgical
~~	~~~	Miss Patrycja Jastrzebska	Point of contact (1st)
~~	~~~	Miss Weronika Rabsztyn	Point of contact (1st)
~~	~~~	Mrs Kerrie Doyle	Point of contact (1st)
~~	~~~	Ms Sandra Mukkath	Point of contact (1st)
~~	~~~	Ms Sylwia Bekulart	Point of contact (1st)
~~	~~~	Mrs Ann Murphy	Research Nurse
~~	~~~	Mrs Anne Butterfield	Research Nurse
~~	~~~	Mrs Jo Wilson	Research Nurse
~~	~~~	Mrs Kerrie Marston	Research Nurse
~~	~~~	Ms Jane Boutflower	Research Nurse
~~	~~~	Ms Phillippa Kyffin	Research Nurse
~~	~~~	Ms Sarah Lawrey	Research Nurse
~~	~~~	Ms Sarah Markus	Research Nurse
~~	~~~	Mr Dave Barber	Trial Coordinator
~~	~~~	Ms Abimbola Aiku	Trial Coordinator
~~	~~~	Mr Matthew Mooney	Data Manager
~~	~~~	Mr Naveen Sankighatta	Data Manager
~~	~~~	Mr Tim Coutts	Data Manager
~~	~~~	Ms Trish Green	Data Manager
~~	~~~	Ms Nicole Langridge	Administrator
~~	~~~	Miss Grace Samkange	Radiographer

INVESTIGATORS AND COLLABORATORS: SITE STAFF
Staff on site delegation logs

Country	Site	Names	Role
~~	~~~	Miss Lisa Durrant	Radiographer
~~	~~~	Mrs Sarah Ruane	Radiographer
~~	~~~	Ms Weronika Carroll	Radiographer
~~	~~~	Ms Sandie Wellman	Other
UK	Peterborough: Peterborough City Hosp	Dr Abigail Hollingdale	Site PI
~~	~~~	Ms Kerrie Cavanagh	Point of contact (1st)
UK	Peterborough: Peterborough District Hosp	Dr Debbie Gregory	Site PI
~~	~~~	Dr Charlotte Ingle	Clinical/Surgical
~~	~~~	Dr Richard Benson	Clinical/Surgical
~~	~~~	Ms Elisa Barter	Clinical/Surgical
~~	~~~	Ms Paula-Joanne Rooney	Research Nurse
~~	~~~	Ms Susan Allen	Trial Coordinator
~~	~~~	Miss Steph Lawrence	Administrator
UK	Plymouth: Derriford Hosp	Mr Henry Sells	Site PI
~~	~~~	Dr Esther McLarty	Clinical/Surgical
~~	~~~	Dr Salvatore Natale	Clinical/Surgical
~~	~~~	Dr Sarah Pascoe	Clinical/Surgical
~~	~~~	Mr John Christopher Hammonds	Clinical/Surgical
~~	~~~	Mr Paul McInerney	Clinical/Surgical
~~	~~~	Mr Keith Purcell	Point of contact (1st)
~~	~~~	Ms Lyn Cogley	Point of contact (1st)
~~	~~~	Miss Emma Bishop	Point of contact (2nd)
~~	~~~	Ms Sharah Tyner	Point of contact (2nd)
~~	~~~	Ms Victoria yates	Point of contact (2nd)
~~	~~~	Ms Maria Brennan	Research Nurse
UK	Poole: Poole Hosp	Dr Joe Davies	Site PI
~~	~~~	Dr Perric Crellin	Clinical/Surgical
~~	~~~	Dr Sathish Harinarayanan	Clinical/Surgical
~~	~~~	Dr Sue Brock	Clinical/Surgical
~~	~~~	Mrs Louise Heckford	Point of contact (1st)
~~	~~~	Ms Sophie Rix	Point of contact (1st)
~~	~~~	Mr Neal Beamish	Point of contact (2nd)
~~	~~~	Mr Roger Wheelwright	Research Nurse
~~	~~~	Ms Amanda Iskender	Research Nurse

INVESTIGATORS AND COLLABORATORS: SITE STAFF
Staff on site delegation logs

Country	Site	Names	Role
~~	~~~	Ms Elizabeth Clarke	Research Nurse
~~	~~~	Ms Hilary Blaney	Research Nurse
~~	~~~	Ms Sandy Pressdee	Research Nurse
~~	~~~	Ms Seonaid Wright	Research Nurse
~~	~~~	Mrs Josie Goodsell	Trial Coordinator
~~	~~~	Miss Elizabeth Woodward	Data Manager
~~	~~~	Miss Nichola Downs	Data Manager
~~	~~~	Ms Sally Gillespie	Data Manager
~~	~~~	Ms Sara Orford	Data Manager
~~	~~~	Ms Teresa Coffin	Administrator
~~	~~~	Miss Felicity Clapp	Radiographer
UK	Portsmouth: Queen Alexandra Hosp	Dr Maja Uherek	Site PI
~~	~~~	Dr Yoodhvir Nagar	Site PI
~~	~~~	Dr Azarel Virgo	Clinical/Surgical
~~	~~~	Dr Ghassan Khoury	Clinical/Surgical
~~	~~~	Dr Kudingila Madhava	Clinical/Surgical
~~	~~~	Ms Mila Roca	Point of contact (1st)
~~	~~~	Mrs Jennifer Hale	Point of contact (2nd)
~~	~~~	Mrs Tracey Dobson	Point of contact (2nd)
~~	~~~	Ms Anna Stephenson	Point of contact (2nd)
~~	~~~	Mrs Angie Harris-Burland	Research Nurse
~~	~~~	Mrs Wendy Stacey	Research Nurse
~~	~~~	Ms Lorna Meadows	Administrator
~~	~~~	Ms Catrin Watkinson	Pharmacist
~~	~~~	Ms Kathy Blight	Pharmacist
UK	Preston: Royal Preston Hosp	Dr Alison Birtle	Site PI
~~	~~~	Dr Marcus Wise	Clinical/Surgical
~~	~~~	Dr Omi Parikh	Clinical/Surgical
~~	~~~	Ms Catherine Walmsley	Point of contact (1st)
~~	~~~	Ms Zainab Chauhan	Point of contact (2nd)
~~	~~~	Miss Rose Ellard	Research Nurse
~~	~~~	Mr Billy Hefferon	Research Nurse
~~	~~~	Mrs Claire Searle	Research Nurse
~~	~~~	Mrs Sandra Curtis	Research Nurse

INVESTIGATORS AND COLLABORATORS: SITE STAFF

Staff on site delegation logs

Country	Site	Names	Role
~~	~~~	Ms Stephanie Cornthwaite	Research Nurse
~~	~~~	Mr Nathan Fish	Data Manager
~~	~~~	Ms Helen Spickett	Data Manager
UK	Reading: Royal Berkshire Hosp	Dr Paul Rogers	Site PI
~~	~~~	Dr Richard B Brown	Clinical/Surgical
~~	~~~	Mr Stephen Parr	Point of contact (1st)
~~	~~~	Ms Emma Vowell	Point of contact (1st)
~~	~~~	Ms Jane Atkinson	Point of contact (1st)
~~	~~~	Mrs Christina Lewis	Point of contact (2nd)
~~	~~~	Mrs Wioletta Kowalczyk-Williams	Research Nurse
~~	~~~	Ms Allison Hunt	Research Nurse
~~	~~~	Ms Helen Purdon	Research Nurse
~~	~~~	Ms Kristy Coomber	Research Nurse
~~	~~~	Ms Debbie Cartwright	Trial Coordinator
UK	Redditch: Alexandra Hosp	Dr Lisa Capaldi	Site PI
~~	~~~	Dr Joanna Hamilton	Clinical/Surgical
~~	~~~	Mrs Alison Harrison	Point of contact (1st)
~~	~~~	Mrs Hayley Hodson	Point of contact (2nd)
~~	~~~	Mrs Sarah Moss	Research Nurse
~~	~~~	Mrs Wendy Featherstone	Research Nurse
~~	~~~	Ms Andrea Isaew	Research Nurse
~~	~~~	Ms Jeanette Knapp	Research Nurse
~~	~~~	Ms Margaret Hindle	MDT coordinator
UK	Romford: Queen's Hosp (Romford)	Dr Maria Martinou	Site PI
~~	~~~	Dr Stephanie Gibbs	Site PI
~~	~~~	Prof Saad Tahir	Site PI
~~	~~~	Dr Ramachandran Subramaniam	Clinical/Surgical
~~	~~~	Mr Anand Kelkar	Clinical/Surgical
~~	~~~	Mr Mohammad Tanvir Vandal	Clinical/Surgical
~~	~~~	Mr Sandeep Gujral	Clinical/Surgical
~~	~~~	Mr Neale O'Brien	Point of contact (1st)
~~	~~~	Thi Vu	Point of contact (1st)
~~	~~~	Mr Revanth Jannapureddy	Point of contact (2nd)
~~	~~~	Ms Tina Mills-Baldock	Research Nurse

INVESTIGATORS AND COLLABORATORS: SITE STAFF
Staff on site delegation logs

Country	Site	Names	Role
~~	~~~	Mr Alastair Nicholson	Data Manager
UK	Salford: Salford Royal Hosp	Prof Noel Clarke	Site PI
~~	~~~	Dr Anna Tran	Clinical/Surgical
~~	~~~	Dr Richard Cowan	Clinical/Surgical
~~	~~~	Mr Chris Betts	Clinical/Surgical
~~	~~~	Mr David Shackley	Clinical/Surgical
~~	~~~	Mr Kieran O'Flynn	Clinical/Surgical
~~	~~~	Mr Maurice Lau	Clinical/Surgical
~~	~~~	Mr Tony Elliott	Clinical/Surgical
~~	~~~	Miss Kay Goulden	Point of contact (1st)
~~	~~~	Mrs Siny George	Point of contact (1st)
~~	~~~	Ms Cellins Vinod	Point of contact (1st)
~~	~~~	Ms Catherine Redshaw	Research Nurse
~~	~~~	Ms Helen Farrell	Research Nurse
~~	~~~	Ms Rachael Allen	Research Nurse
~~	~~~	Ms Soney Dharmaprasad	Research Nurse
~~	~~~	Sister Jill Youd	Research Nurse
~~	~~~	Sister Melanie Taylor	Research Nurse
~~	~~~	Sister Sarah Kirk	Research Nurse
~~	~~~	Mr Garry Stevenson	Trial Coordinator
~~	~~~	Mrs Chloe-Anne Thompson	Data Manager
~~	~~~	Mrs Christine Farnworth	Data Manager
~~	~~~	Miss Danielle Platt	Administrator
~~	~~~	Mr Oliver Wadsworth	Administrator
~~	~~~	Ms Karen Richardson	Administrator
~~	~~~	Ms Leah Harter	Administrator
~~	~~~	Ms Malan Kaushal	Administrator
~~	~~~	Mrs Amanda Cordwell	MDT coordinator
~~	~~~	Ms Amanda Bowmer	MDT coordinator
~~	~~~	Mrs Claire Duncan (nee Keatley)	Pharmacist
~~	~~~	Ms Anne Marie Lydon	Other
UK	Salisbury: Salisbury District Hosp	Dr Adityanarayan Bhatnagar	Site PI
~~	~~~	Mr Allister Campbell	Clinical/Surgical
~~	~~~	Mr Gregor Mcintosh	Clinical/Surgical

INVESTIGATORS AND COLLABORATORS: SITE STAFF
Staff on site delegation logs

Country	Site	Names	Role
~~	~~~	Mr Mohammed El-Saghir	Clinical/Surgical
~~	~~~	Mr Peter Guy	Clinical/Surgical
~~	~~~	Mrs Lehentha Mattocks	Point of contact (1st)
~~	~~~	Mrs Catherine Reed	Research Nurse
~~	~~~	Mrs Sarah Bunn	Research Nurse
~~	~~~	Ms Julie Attlee	Research Nurse
~~	~~~	Ms Ruth Fennelly	Trial Coordinator
~~	~~~	Mrs Sophia Strong-Sheldrake	Administrator
~~	~~~	Ms Julie Gwilt	Administrator
~~	~~~	Ms Gemma Tedd	MDT coordinator
~~	~~~	Mrs Brenda Murphy	Pharmacist
UK	Scunthorpe: Scunthorpe General Hosp	Dr Sanjay Dixit	Site PI
~~	~~~	Mrs Sue Spencer	Point of contact (1st)
~~	~~~	Ms Kathleen Dent	Point of contact (1st)
~~	~~~	Ms Sandra Pearson	Point of contact (1st)
~~	~~~	Ms Dorota Potoczna	Point of contact (2nd)
~~	~~~	Mrs Karen Martin	Research Nurse
~~	~~~	Ms Jo Towse	Data Manager
~~	~~~	Ms Nikita Whotton	Data Manager
~~	~~~	Ms Marion Hood	Administrator
UK	Sheffield: Weston Park Hosp	Dr Catherine Ferguson	Site PI
~~	~~~	Dr Jessica Tay	Site PI
~~	~~~	Dr Jackie Martin	Clinical/Surgical
~~	~~~	Dr Katie Bowen	Clinical/Surgical
~~	~~~	Dr Peter Kirkbride	Clinical/Surgical
~~	~~~	Ms Rachael Clarke	Point of contact (1st)
~~	~~~	Sister Kim Wood	Research Nurse
~~	~~~	Mrs Kate Gibbins	Trial Coordinator
~~	~~~	Miss Alexandra Firth	Data Manager
~~	~~~	Mr John Martindale	Data Manager
~~	~~~	Mrs Janine Smedley (nee McCabe)	Data Manager
~~	~~~	Ms Su Clark	Data Manager
~~	~~~	Ms Catherine Spalton	Research Asst
~~	~~~	Ms Suzanne Smith	Administrator

INVESTIGATORS AND COLLABORATORS: SITE STAFF

Staff on site delegation logs

Country	Site	Names	Role
UK	Shrewsbury: Royal Shrewsbury Hosp	Dr Narayanan Srihari	Site PI
~~	~~~	Mr Ravi Prashant	Clinical/Surgical
~~	~~~	Mr Sanal Jose	Point of contact (1st)
~~	~~~	Ms Emma Bates	Point of contact (1st)
~~	~~~	Ms Indukala Chennattukungu	Point of contact (1st)
~~	~~~	Ms Joanna Clancy	Point of contact (1st)
~~	~~~	Mrs Hayley Hughes	Research Nurse
~~	~~~	Mrs Sunita Kurian-Downer	Research Nurse
~~	~~~	Ms Elena Michael	Research Nurse
~~	~~~	Ms Karen Nicholas	Research Nurse
~~	~~~	Ms Sally Potts	Research Nurse
~~	~~~	Ms Vanessa Cross	Research Nurse
~~	~~~	Sister Helen Moore	Research Nurse
~~	~~~	Mrs Marion Adams	Trial Coordinator
~~	~~~	Miss Suzanne Pope	Data Manager
~~	~~~	Mrs Sandra Smith	Data Manager
~~	~~~	Mrs Harpreet Singh	Administrator
~~	~~~	Mrs Emma Weaver	Radiographer
UK	Slough: Wexham Park Hosp	Dr Nicola Dallas	Site PI
~~	~~~	Dr Helen O'Donnell	Clinical/Surgical
~~	~~~	Dr M Laniado	Clinical/Surgical
~~	~~~	Dr Richard B Brown	Clinical/Surgical
~~	~~~	Dr Shahid Sharif	Clinical/Surgical
~~	~~~	Mr Omer Karim	Clinical/Surgical
~~	~~~	Miss Nicky Barnes	Point of contact (1st)
~~	~~~	Miss Nicole Kader	Point of contact (2nd)
~~	~~~	Ms Ana Sierra Pala	Point of contact (2nd)
~~	~~~	Mrs Victoria Robinson	Research Nurse
~~	~~~	Ms Alison Sears	Research Nurse
~~	~~~	Ms Jayne Litchfield	Research Nurse
~~	~~~	Sister Ann Jackson	Research Nurse
~~	~~~	Sister Catherine Smith	Research Nurse
~~	~~~	Mrs Julie-Ann Sinclair	Data Manager
~~	~~~	Ms Sana Mahmood	Pharmacist

INVESTIGATORS AND COLLABORATORS: SITE STAFF
Staff on site delegation logs

Country	Site	Names	Role
~~	~~~	Dr Ali Abbas	Other
UK	Somerset: Musgrove Park Hosp	Dr Emma Gray	Site PI
~~	~~~	Dr Faith McMeekin	Clinical/Surgical
~~	~~~	Dr John Boardman	Clinical/Surgical
~~	~~~	Dr John Graham	Clinical/Surgical
~~	~~~	Dr Joseph Jelski	Clinical/Surgical
~~	~~~	Dr Manivannan Periasamy	Clinical/Surgical
~~	~~~	Dr Manjusha Keni	Clinical/Surgical
~~	~~~	Dr Mary Tighe	Clinical/Surgical
~~	~~~	Dr Mohini Varughese	Clinical/Surgical
~~	~~~	Mr Surayne Segaran	Clinical/Surgical
~~	~~~	Ms Jeanette Bowes-Cavanagh	Clinical/Surgical
~~	~~~	Miss Rebecca Brown	Point of contact (1st)
~~	~~~	Mrs Sue Mahoney	Point of contact (1st)
~~	~~~	Ms Alison Whitcher	Point of contact (1st)
~~	~~~	Ms Sara Green	Point of contact (1st)
~~	~~~	Mr Tamlyn Russell	Point of contact (2nd)
~~	~~~	Mrs Maria Zietz	Point of contact (2nd)
~~	~~~	Ms Jayne Foot	Point of contact (2nd)
~~	~~~	Ms Alison Chedham	Research Nurse
~~	~~~	Ms Angela Locke	Research Nurse
~~	~~~	Ms Martha Wrigley	Research Nurse
~~	~~~	Mrs Michelle Farrar	Trial Coordinator
~~	~~~	Ms Flora Darch	Trial Coordinator
~~	~~~	Ms Christine Webster	Data Manager
~~	~~~	Mrs Sarah Wiggins	Administrator
~~	~~~	Ms Jan Ashcroft	Administrator
~~	~~~	Ms Rebecca Wallbutton	Administrator
~~	~~~	Mr Simon Goldsworthy	Radiographer
~~	~~~	Miss Odunayo Kalejaiye	Other
~~	~~~	Ms Elizabeth Ruszala	Other
UK	Southampton: Southampton General Hosp	Dr Catherine Heath	Site PI
~~	~~~	Dr Alec Paschalis	Clinical/Surgical
~~	~~~	Dr Mark Noble	Clinical/Surgical

INVESTIGATORS AND COLLABORATORS: SITE STAFF
Staff on site delegation logs

Country	Site	Names	Role
~~	~~~	Dr Victoria McFarlane	Clinical/Surgical
~~	~~~	Ms Anna Stephenson	Point of contact (1st)
~~	~~~	Ms Annelise Haskell	Point of contact (1st)
~~	~~~	Ms Fabiola Morales-Azofra	Point of contact (1st)
~~	~~~	Ms Lucy Galloway	Point of contact (1st)
~~	~~~	Ms Malavika Ganabady	Point of contact (1st)
~~	~~~	Ms Carina Mundy	Research Nurse
~~	~~~	Ms Kirsty Cumming	Research Nurse
~~	~~~	Ms Naomi James	Research Nurse
~~	~~~	Mrs Julie Kennedy	Trial Coordinator
~~	~~~	Mrs Julie Patrick	Trial Coordinator
~~	~~~	Mrs Susan Morton	Trial Coordinator
~~	~~~	Ms Shauna Wakefield	Trial Coordinator
~~	~~~	Ms Julie Abab	Administrator
~~	~~~	Ms Julie Gwilt	Administrator
~~	~~~	Ms Leanne Reader	Administrator
~~	~~~	Mrs Lisa Taylor	MDT coordinator
UK	Southend: Southend University Hosp	Dr David Tsang	Site PI
~~	~~~	Dr Abby Cyriac	Clinical/Surgical
~~	~~~	Dr Imtiaz Ahmed	Clinical/Surgical
~~	~~~	Dr Jan Prejbisz	Clinical/Surgical
~~	~~~	Dr Olivia Chan	Clinical/Surgical
~~	~~~	Mrs Tracey Davies	Point of contact (1st)
~~	~~~	Ms Sheila Reece	Research Nurse
~~	~~~	Ms Sue Bowman	Trial Coordinator
~~	~~~	Ms Heather Shires	Administrator
~~	~~~	Ms Katrina Maitland	Other
UK	Stockport: Stepping Hill Hosp	Dr John Logue	Site PI
~~	~~~	Mr WA Brough	Site PI
~~	~~~	Mr Gerald Collins	Clinical/Surgical
~~	~~~	Mr Richard Brough	Clinical/Surgical
~~	~~~	Mr Elliott Wiss	Point of contact (1st)
~~	~~~	Ms Sarah Connolly nee McKenna	Point of contact (1st)
~~	~~~	Ms Sarah Smallwood	Point of contact (1st)

INVESTIGATORS AND COLLABORATORS: SITE STAFF
Staff on site delegation logs

Country	Site	Names	Role
~~	~~~	Ms Sheila Hodgkinson	Point of contact (1st)
~~	~~~	Mrs Tricia Coughlan	Point of contact (2nd)
~~	~~~	Ms Emma Goodwin	Point of contact (2nd)
~~	~~~	Mrs Helen Haydock	Research Nurse
~~	~~~	Ms Christina Gilmour	Research Nurse
~~	~~~	Ms Eleanor Anscombe	Research Nurse
~~	~~~	Ms Sam Corcoran	Research Nurse
~~	~~~	Ms Jill Taylor	Data Manager
~~	~~~	Ms Tracie Cocks	Data Manager
~~	~~~	Ms Pat Clitheroe	Administrator
~~	~~~	Mrs Carol Rotherham	MDT coordinator
~~	~~~	Mr John Kilmartin	Pharmacist
~~	~~~	Ms Lucy Orrell	Pharmacist
~~	~~~	Ms Susan Graham	Pharmacist
~~	~~~	Dr Umi Hatimy	Other
UK	Stoke: Royal Stoke University Hosp	Dr Salil Vengalil	Site PI
~~	~~~	Dr Fawzi Adab	Clinical/Surgical
~~	~~~	Dr Kathirvelu Dhinakaran	Clinical/Surgical
~~	~~~	Dr Rajanee Bhana	Clinical/Surgical
~~	~~~	Mrs Angela Peake	Point of contact (1st)
~~	~~~	Ms Georgia Thomasson	Point of contact (1st)
~~	~~~	Miss Elizabeth Sellars	Point of contact (2nd)
~~	~~~	Ms Katrina Parkinson	Point of contact (2nd)
~~	~~~	Mrs Angela Ward	Research Nurse
~~	~~~	Mrs Marion Evans	Research Nurse
~~	~~~	Ms Alison Myatt	Research Nurse
~~	~~~	Ms Jenny Walton	Research Nurse
~~	~~~	Ms Julie Storer	Data Manager
~~	~~~	Ms Rowena Smith	Radiographer
UK	Sutton-in-Ashfield: King's Mill Hosp	Dr Eliot Chadwick	Site PI
~~	~~~	Dr Alastair McCabe	Clinical/Surgical
~~	~~~	Dr Daniel Saunders	Clinical/Surgical
~~	~~~	Dr Georgina Walker	Clinical/Surgical
~~	~~~	Dr Jun Lim	Clinical/Surgical

INVESTIGATORS AND COLLABORATORS: SITE STAFF
Staff on site delegation logs

Country	Site	Names	Role
~~	~~~	Dr Santhanam Sundar	Clinical/Surgical
~~	~~~	Mr Dominic Nash	Point of contact (1st)
~~	~~~	Ms Susan Smith	Point of contact (1st)
~~	~~~	Miss Jamie-Rae Burgoyne	Data Manager
~~	~~~	Mr Steve Haigh	Pharmacist
~~	~~~	Mrs Samantha Boam	Pharmacist
~~	~~~	Ms Keri Hollis	Pharmacist
~~	~~~	Ms Lyndsey Munson	Pharmacist
~~	~~~	Ms Lynne Wade	Pharmacist
~~	~~~	Dr Shafiq Gill	Other
UK	Swansea: Singleton Hosp	Dr Mau-Don Phan	Site PI
~~	~~~	Dr Delia Pudney	Clinical/Surgical
~~	~~~	Miss Ellen Tait	Point of contact (1st)
~~	~~~	Miss Nicola Lemon	Point of contact (1st)
~~	~~~	Ms Bethan Williams	Point of contact (1st)
~~	~~~	Ms Emily Harris (n. Marchant)	Point of contact (1st)
~~	~~~	Ms Elizabeth Evans	Point of contact (2nd)
~~	~~~	Ms Maria Johnstone	Point of contact (2nd)
~~	~~~	Mrs Alex Franklin	Research Nurse
~~	~~~	Ms Alison Stretch	Research Nurse
~~	~~~	Mr Lewis Jones	Trial Coordinator
~~	~~~	Ms Rachael Spence	Radiographer
UK	Swindon: Great Western Hosp	Dr Omar Khan	Site PI
~~	~~~	Dr David J Cole	Clinical/Surgical
~~	~~~	Dr Shiroma De Silva-Minor	Clinical/Surgical
~~	~~~	Ms Suzannah Pegler	Point of contact (1st)
~~	~~~	Ms Abbie Poole	Point of contact (2nd)
~~	~~~	Mr Vivian Zinyemba	Research Nurse
~~	~~~	Mrs Debbie Palmer	Research Nurse
~~	~~~	Ms Ania Jones	Research Nurse
~~	~~~	Ms Cerila Parajes	Research Nurse
~~	~~~	Ms Ellen Starling	Research Nurse
~~	~~~	Sister Helen Winter	Research Nurse
~~	~~~	Sister Jan Dodge	Research Nurse

INVESTIGATORS AND COLLABORATORS: SITE STAFF
Staff on site delegation logs

Country	Site	Names	Role
~~	~~~	Sister Tracey Sargent	Research Nurse
~~	~~~	Mrs Sarah Grayland	Research Asst
~~	~~~	Mr Tim Owen	Administrator
~~	~~~	Mrs Rebecca Belcher	Administrator
~~	~~~	Ms Becky Taylor	Administrator
UK	Torbay: Torbay District General Hosp	Dr Anna Lydon	Site PI
~~	~~~	Dr Erica Watts	Clinical/Surgical
~~	~~~	Dr Jorg Michels	Clinical/Surgical
~~	~~~	Dr Rajaguru Srinivasan	Clinical/Surgical
~~	~~~	Mr Jon Buckley	Point of contact (1st)
~~	~~~	Mrs Michele Allison	Point of contact (1st)
~~	~~~	Mrs Shelley Chamberlain	Point of contact (1st)
~~	~~~	Miss Linda Welsh	Point of contact (2nd)
~~	~~~	Mrs Donna Cuffe	Research Nurse
~~	~~~	Mrs Elaine Vandecandalaere	Research Nurse
~~	~~~	Ms Catherine Brookman	Research Nurse
~~	~~~	Ms Helen Greedus	Research Nurse
~~	~~~	Ms Ingrid Koehler	Research Nurse
~~	~~~	Ms Lorraine Thornton	Research Nurse
~~	~~~	Miss Claire Fairfax	Administrator
~~	~~~	Miss Hannah Griffin	Administrator
~~	~~~	Miss Stacey Davies	Administrator
~~	~~~	Ms Julia Pym	Administrator
~~	~~~	Mr Martyn Blundell	Pharmacist
~~	~~~	Dr Fiona Roberts	Other
UK	Wakefield: Pinderfields Hosp	Dr Juliette Anderson	Site PI
~~	~~~	Dr Ann Henry	Clinical/Surgical
~~	~~~	Dr Catherine Coyle	Clinical/Surgical
~~	~~~	Dr Chris Fosker	Clinical/Surgical
~~	~~~	Dr Nathalie Casanova	Clinical/Surgical
~~	~~~	Dr Peter Dickinson	Clinical/Surgical
~~	~~~	Dr Sree Rodda	Clinical/Surgical
~~	~~~	Mr Philip Weston	Clinical/Surgical
~~	~~~	Mr Rohit Chahal	Clinical/Surgical

INVESTIGATORS AND COLLABORATORS: SITE STAFF
Staff on site delegation logs

Country	Site	Names	Role
~~	~~~	Mr Subramanian Kanaga Sundaram	Clinical/Surgical
~~	~~~	Mr Subramanian Kanaga-Sundaram	Clinical/Surgical
~~	~~~	Mr Jim Anderson	Point of contact (1st)
~~	~~~	Mr Stephen Littler	Point of contact (1st)
~~	~~~	Mr Jonathan Slack	Point of contact (2nd)
~~	~~~	Ms Beverley Taylor	Point of contact (2nd)
~~	~~~	Ms Barbara Burlace	Research Nurse
~~	~~~	Ms Janine Heeley	Administrator
~~	~~~	Ms Julie Ball	Administrator
~~	~~~	Mr Richard Bowers	Pharmacist
~~	~~~	Ms Louise Benton	Pharmacist
UK	Warwick: Warwick Hosp	Dr Andrew Chan	Site PI
~~	~~~	Dr Andrew Stockdale	Clinical/Surgical
~~	~~~	Mr Ashley Johnson-Rollings	Point of contact (1st)
~~	~~~	Mrs Tina Gamble	Point of contact (1st)
~~	~~~	Ms Donna Walsh	Point of contact (1st)
~~	~~~	Ms Jo Williams	Point of contact (2nd)
~~	~~~	Mrs Elaine Simmons	Research Nurse
~~	~~~	Mrs Helen Millage	Research Nurse
~~	~~~	Ms Eilish O’Neill	Research Nurse
~~	~~~	Ms Kerrie Webb	Research Nurse
~~	~~~	Ms Lyn Hartwell	Research Nurse
~~	~~~	Mrs Theresa Griffiths	Data Manager
~~	~~~	Mrs Julia Jones	Pharmacist
~~	~~~	Ms Judith Chettle	Pharmacist
UK	Weston-Super-Mare: Weston General Hosp	Dr Serena Hilman	Site PI
~~	~~~	Dr Symeon Eleftheriadis	Clinical/Surgical
~~	~~~	Dr Tom Wells	Clinical/Surgical
~~	~~~	Mr Harvey Dymond	Point of contact (1st)
~~	~~~	Mr Hugh Lloyd-Jones	Research Nurse
~~	~~~	Mr John Anderson	Administrator
UK	Wirral: Clatterbridge Centre for Oncology	Dr Isabel Syndikus	Site PI
~~	~~~	Dr Helen Innes	Clinical/Surgical
~~	~~~	Dr John Littler	Clinical/Surgical

INVESTIGATORS AND COLLABORATORS: SITE STAFF
Staff on site delegation logs

Country	Site	Names	Role
~~	~~~	Dr Shaun Tolan	Clinical/Surgical
~~	~~~	Mrs Kathryn Hughes	Point of contact (1st)
~~	~~~	Mrs Sarah Bennett	Point of contact (1st)
~~	~~~	Mrs Sharon Dunn	Point of contact (1st)
~~	~~~	Ms Alison Kelly	Point of contact (1st)
~~	~~~	Ms Nikki Miller	Point of contact (1st)
~~	~~~	Mr Matthew Stott	Research Nurse
~~	~~~	Mrs Diane Fildes	Research Nurse
~~	~~~	Ms Alison Hassall	Research Nurse
~~	~~~	Ms Gaynor Herbert	Research Nurse
~~	~~~	Mr Laurie Lomax	Trial Coordinator
~~	~~~	Mr Chris Nutman	Data Manager
~~	~~~	Ms Alison Weston	Data Manager
UK	Wolverhampton: New Cross Hosp	Dr Ian Sayers	Site PI
~~	~~~	Dr Ali Samanci	Clinical/Surgical
~~	~~~	Dr Mark Churn	Clinical/Surgical
~~	~~~	Dr Pek Keng-Koh	Clinical/Surgical
~~	~~~	Mr Peter Cooke	Clinical/Surgical
~~	~~~	Arizoo Mohseeni	Point of contact (1st)
~~	~~~	Miss Renita Pawaroo	Point of contact (1st)
~~	~~~	Mr David Homer	Point of contact (1st)
~~	~~~	Mr Jason Rogers	Point of contact (2nd)
~~	~~~	Mrs Christine Kirk	Research Nurse
~~	~~~	Mrs Claire Lomas	Research Nurse
~~	~~~	Mrs Emma Sharman	Research Nurse
~~	~~~	Mrs Ivanna Baker	Research Nurse
~~	~~~	Ms Anna Grant	Trial Coordinator
~~	~~~	Mrs Hazel Spencer	Data Manager
~~	~~~	Ms Debbie Spruce	Data Manager
~~	~~~	Mrs Liz Radford	Research Asst
~~	~~~	Ms Hannah Worthington	Research Asst
~~	~~~	Ms Marian McCormick	Research Asst
~~	~~~	Ms Jenny Chatfield	Administrator
~~	~~~	Ms Vanda Carter	Other

INVESTIGATORS AND COLLABORATORS: SITE STAFF

Staff on site delegation logs

Country	Site	Names	Role
UK	Worcester: Worcestershire Royal Hosp	Dr Lisa Capaldi	Site PI
~~	~~~	Dr Jo Bowen	Clinical/Surgical
~~	~~~	Dr Kamalnayan Gupta	Clinical/Surgical
~~	~~~	Mr Jacob Taylor	Point of contact (1st)
~~	~~~	Mrs Amanda Holdsworth	Point of contact (1st)
~~	~~~	Ms Jayne Tyler	Point of contact (1st)
~~	~~~	Mrs Dagmara Bak	Point of contact (2nd)
~~	~~~	Mrs Hayley Hodson	Point of contact (2nd)
~~	~~~	Mrs Kristy Cleary	Research Nurse
~~	~~~	Mrs Helen Tranter	Trial Coordinator
~~	~~~	Ms Janet Forkes	Trial Coordinator
~~	~~~	Mrs Patricia Rimell	Data Manager
~~	~~~	Ms Sue Davies	Data Manager
~~	~~~	Miss Kate Ledger	Research Asst
~~	~~~	Ms Jennifer Healey-Mariano	Research Asst
~~	~~~	Mr Hugh Morrow	Pharmacist
~~	~~~	Mrs Ann White	Pharmacist
~~	~~~	Mrs Monica Gauntlett	Pharmacist
~~	~~~	Ms Alison Rosoman	Pharmacist
~~	~~~	Ms Heather Perry	Pharmacist
UK	Worthing: Worthing Hosp	Dr Ashok Nikapota	Site PI
~~	~~~	Dr David Bloomfield	Clinical/Surgical
~~	~~~	Dr George Plataniotis	Clinical/Surgical
~~	~~~	Miss Raquel Gomez-Marcos	Point of contact (1st)
~~	~~~	Ms Chloe Hoskins	Point of contact (1st)
~~	~~~	Ms Marian Flynn-Batham	Point of contact (1st)
~~	~~~	Ms Sarah Rippin	Point of contact (1st)
~~	~~~	Mr Tan Tsawayo	Point of contact (2nd)
~~	~~~	Mrs Sarah Funnell	Point of contact (2nd)
~~	~~~	Miss Paula Wakelen	Research Nurse
~~	~~~	Ms Dawn Crowe (nee Hughes)	Research Nurse
~~	~~~	Ms Jeanette Gilbert	Research Nurse
~~	~~~	Ms Linda Folkes	Research Nurse
~~	~~~	Ms Nikki Turner	Research Nurse

INVESTIGATORS AND COLLABORATORS: SITE STAFF
Staff on site delegation logs

Country	Site	Names	Role
~~	~~~	Ms Sally Moore	Research Nurse
~~	~~~	Mr Matthew Smith	Trial Coordinator
~~	~~~	Ms Celia Gonzalez	Pharmacist
UK	Wycombe: Wycombe Hosp	Dr Katherine Hyde	Site PI
~~	~~~	Dr Ami Sabharwal	Clinical/Surgical
~~	~~~	Dr Andrew Weaver	Clinical/Surgical
~~	~~~	Dr Joanne Brady	Clinical/Surgical
~~	~~~	Dr Niki Panakis	Clinical/Surgical
~~	~~~	Dr Philip Camilleri	Clinical/Surgical
~~	~~~	Dr Thinn Pwint	Clinical/Surgical
~~	~~~	Prof Andrew Protheroe	Clinical/Surgical
~~	~~~	Dr Janice Carpenter	Point of contact (1st)
~~	~~~	Mrs Anita Cserbane	Point of contact (1st)
~~	~~~	Mrs Alice Ngumo	Point of contact (2nd)
~~	~~~	Ms Emma Hogbin	Research Nurse
~~	~~~	Ms Hazel Wynn	Research Nurse
~~	~~~	Ms Sarah Manyangadze	Research Nurse
~~	~~~	Mrs Manisha Joshi	Data Manager
~~	~~~	Miss Claire Fernandez	Research Asst
~~	~~~	Ms Susan McLain-Smith	Research Asst
~~	~~~	Mr Rahul Kurup	Administrator
~~	~~~	Mr Neil Trew-Smith	Pharmacist
~~	~~~	Ms Roisin Kavanagh	Pharmacist
~~	~~~	Mr Neil Trewsmith	Other
UK	Yeovil: Yeovil District Hosp	Mr Tim Porter	Site PI
~~	~~~	Dr Emma Gray	Clinical/Surgical
~~	~~~	Dr Erica Beaumont	Clinical/Surgical
~~	~~~	Dr Geoffrey Sparrow	Clinical/Surgical
~~	~~~	Dr Shiyam Kumar	Clinical/Surgical
~~	~~~	Mrs Joanna Allison	Point of contact (1st)
~~	~~~	Mrs Joanne Macrory	Point of contact (1st)
~~	~~~	Mrs Kerry Rennie	Point of contact (1st)
~~	~~~	Miss Jess Perry	Point of contact (2nd)
~~	~~~	Mr Nigel Beer	Research Nurse

INVESTIGATORS AND COLLABORATORS: SITE STAFF
Staff on site delegation logs

Country	Site	Names	Role
~~	~~~	Mrs Lucy Pippard	Research Nurse
~~	~~~	Ms Shirley Fox	Research Nurse
~~	~~~	Ms Barbara Williams-Yesson	Trial Coordinator
~~	~~~	Miss Kate Beesley	Administrator
~~	~~~	Mrs Michelle Kotze	Administrator
~~	~~~	Miss Claire Barron	Pharmacist
~~	~~~	Mr David Donaldson	Pharmacist
~~	~~~	Mrs Tracey Duckett	Pharmacist
UK	York: York District Hosp	Dr Joji Joseph	Site PI
~~	~~~	Dr David Bottomley	Clinical/Surgical
~~	~~~	Dr Hima Bindu Musunuru	Clinical/Surgical
~~	~~~	Dr Russ Wilson	Clinical/Surgical
~~	~~~	Mr Michael Stower	Clinical/Surgical
~~	~~~	Mr Paul Brittain	Point of contact (1st)
~~	~~~	Mrs Flor Davies	Point of contact (1st)
~~	~~~	Mrs Paula Strider	Point of contact (1st)
~~	~~~	Mrs Claire Brookes	Point of contact (2nd)
~~	~~~	Mr Mark Fearnley	Research Nurse
~~	~~~	Mrs Jane Balderson	Research Nurse
~~	~~~	Mrs Jo Ingham	Research Nurse
~~	~~~	Mrs Lisa Mole	Research Nurse
~~	~~~	Mrs Nora Youngs	Research Nurse
~~	~~~	Ms Carol Taylor	Research Nurse
~~	~~~	Ms Emma Giddings	Research Nurse
~~	~~~	Ms Fereshteh Fallah	Research Nurse
~~	~~~	Ms Hilary Campbell	Research Nurse
~~	~~~	Ms Joanna Todd	Research Nurse
~~	~~~	Ms Lucy Howard	Research Nurse
~~	~~~	Mr Andy Holbrook	Trial Coordinator
~~	~~~	Mrs Srilakshmi Gollapothu	Data Manager
~~	~~~	Miss Shamila Saleem	Research Asst
~~	~~~	Mr Louis Thackray	Research Asst
~~	~~~	Ms Monica Campos	Research Asst
~~	~~~	Miss Lorna Bowling	Pharmacist

INVESTIGATORS AND COLLABORATORS: SITE STAFF
Staff on site delegation logs

Country	Site	Names	Role
~~	~~~	Mr John Wightman	Pharmacist
~~	~~~	Mr Mark Elliott	Pharmacist
~~	~~~	Mr Richard Evans	Pharmacist
~~	~~~	Mrs Christine Jakeman	Pharmacist
~~	~~~	Ms Michelle Donnison	Pharmacist
~~	~~~	Ms Rachel Conway	Pharmacist
~~	~~~	Ms Sally Harvey	Pharmacist
~~	~~~	Ms Sarah Pashley	Other

PARTICIPANTS

Approximately 4,000 people chose to participate in RADICALS. In addition to their care teams, they have been supported by family, friends and other key people. Every person who has participated in the trial is appreciated by the trial team and should be appreciated by the wider public. The findings from clinical trials can change practice for the future, but clinical trials only happen because people find the time and make the effort to support them. Thank you.

RADICALS

Radiotherapy and Androgen Deprivation In Combination After Local Surgery

A randomised controlled trial in prostate cancer

Version: 7.0
Date: 17th February 2020

MRC CTU ID: PR10
ISRCTN #: ISRCTN 40814031
NCT #: NCT00541047
EUDRACT #: 2006-000205-34
CTA #: 20363/0402/001-0031
MREC #: 07/Q0501/48

Authorised by:
Name: Professor Chris Parker
Role: Chief Investigator
Date: 17th February 2020
Signature:

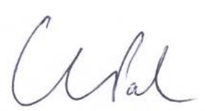

Name: Professor Matthew Sydes
Role: Trial Statistician/Project Lead
Date
Signature:

GENERAL INFORMATION

This document was constructed using the MRC CTU Protocol Template Version 7.0. It describes the RADICALS trial, coordinated by the Medical Research Council (MRC) Clinical Trials Unit (CTU) at University College London (UCL), and provides information about procedures for entering patients into it. The protocol should not be used as an aide-memoire or guide for the treatment of other patients. Every care has been taken in drafting this protocol, but corrections or amendments may be necessary. These will be circulated to the registered investigators in the trial, but sites entering patients for the first time are advised to contact the trial team to confirm they have the most up-to-date version.

COMPLIANCE

The trial will be conducted in compliance with the approved protocol, the Declaration of Helsinki 1996, the principles of Good Clinical Practice (GCP) as laid down by the ICH topic E6 (R2), Commission Clinical Trials Directive 2005/28/EC* with the implementation in national legislation in the UK by Statutory Instrument 2004/1031 and subsequent amendments, General Data Protection Regulation and the UK Data Protection Act 2018 (DPA number: Z6364106), and the UK Policy Framework for Health and Social Care Research.

*Until the Clinical Trials Regulation EU No 536/2014 becomes applicable, the trial will be conducted in accordance with the Clinical Trials Directive as implemented in the UK statutory instrument. When the directive is repealed on the day of entry into application of the Clinical Trial Regulation the trial will work towards implementation of the Regulation (536/2014) following any transition period.

International sites will comply with the principles of GCP as laid down by the ICH topic E6 (R2) and other applicable national regulations.

SPONSOR

UCL is the trial Sponsor and has delegated responsibility for the overall management of the trial to the MRC CTU at UCL. Queries relating to UCL sponsorship of this trial should be addressed to Professor Mahesh Parmar, MRC CTU at UCL Director, MRC CTU at UCL, Institute of Clinical Trials & Methodology, 90 High Holborn 2nd Floor, London, WC1V 6LJ.

The Canadian Cancer Trials Group (CCTG) (previously NCIC Clinical Trials Group) have been delegated certain Sponsor activities and functions for Canadian sites.

FUNDING

Funders in UK:	Clinical Trials Advisory Awards Committee (on behalf of Cancer Research UK)<br>Grant Reference Number: C7829/A6381<br>Medical Research Council<br>Grant Reference Number: MC_UU_12023/28
Funder in Canada:	Canadian Cancer Society – Research Institute<br>Grant Reference Number: 704970

AUTHORISATIONS AND APPROVALS

This trial was approved by the Royal Free & Medical School Research Ethics Committee and is part of the UK National Cancer Research Network (NCRN) research network portfolio.

The following persons are authorised to sign the final protocol and protocol amendments for the sponsor: Professor Chris Parker (Chief Investigator) and Professor Matthew Sydes (Trial Statistician/Project Lead).

TRIAL REGISTRATION

This trial has been registered with the ClinicalTrials.gov Clinical Trials Register, where it is identified as NCT00541047.

CLINICAL CENTRES

RADICALS is open to accredited centres in Cancer Research Networks across the UK. It is also open at centres in Canada (in collaboration with the Canadian Cancer Trials Group), Denmark and Ireland.

SAE REPORTING
Within 24 hours of becoming aware of an SAE, please fax a
completed SAE form to the MRC CTU at UCL on:
Fax: +44 (0) 20 7670 4818 or via email to **mrcctu.radicals@ucl.ac.uk**

INFORMATION FOR MRC INVESTIGATORS

SCIENTIFIC APPROVAL

The RADICALS trial has been scientifically approved by the Clinical Trials Awards and Advisory Committee (CTAAC) of Cancer Research UK and is thus part of the NCRN/NCRI portfolio of prostate cancer trials.

ETHICS APPROVAL

Royal Free Hospital Research Ethics Committee. Ref: 07/Q0501/48, 23rd April 2007

REGULATORY APPROVAL

CTA reference 20363/0402/001-0031, 27th April 2007

FINANCE

No payments will be made to centres because approaches are standard, and no free or discounted drugs are provided. This trial is NRCN adopted and therefore UK NCRN nurse time will be available to support the study.

CHIEF INVESTIGATOR/MEDICAL EXPERT

Professor Chris Parker	Royal Marsden Hospital & Institute of Cancer Research, Sutton, SM2 5PT<br>T: + 44 (0)208 661 3425 E: Chris.Parker@icr.ac.uk

TRIAL ADMINISTRATION

Please direct all queries to the Trial Manager at MRC CTU at UCL in the first instance; clinical queries will be passed to the Chief Investigator via the Trial Manager.

COORDINATING UNIT

MRC Clinical Trials Unit at UCL<br>90 High Holborn 2nd Floor<br>London<br>WC1V 6LJ<br>UK	Switchboard:<br>Fax:<br>Email:	020 7670 4700<br>020 7670 4818<br>mrcctu.radicals@ucl.ac.uk

MRC CTU AT UCL STAFF

Trial Manager:	Holly Pickering	extn 4636
Data Manager:	Christos Maniatis	extn 4838
Clinical Project Manager	Silivia Forcat	extn 4825
Statistician:	Adrian Cook	extn 4639
Project Lead:	Matthew Sydes	extn 4798

For full details of all trial committees, please see **Appendix A**
NB: throughout this document, “MRC CTU at UCL” is generally abbreviated to “CTU”.

INFORMATION FOR CCTG INVESTIGATORS

SCIENTIFIC APPROVAL

Canadian Cancer Trials Group (CCTG)

FINANCE

The rate of per case funding is the standard per case funding amount for each patient enrolled at each centre. For more information please see:
http://www.ctg.queensu.ca/trials/generic_forms_public/centre_funding.pdf

CCTG CHIEF INVESTIGATORS/MEDICAL EXPERTS

Charles Catton	Princess Margaret Hospital, Toronto, Canada<br>T :+1-416-946-2000<br>E :charles.catton@rmp.uhn.on.ca
Fred Saad	Montreal University, Montreal, Canada<br>T:+1-514-890-8000 ext 27466<br>E: fredsaad@videotron.ca

CCTG STAFF

CCTG Study Coordinator:	Cathy Davidson	E: cdavidson@ctg.queensu.ca
CCTG Clinical Trials Assistant:	Amy Hawkins	E: ahawkins@ctg.queensu.ca
Senior Investigator:	Wendy Parulekar	E: wparulekar@ctg.queensu.ca

T: +1 613-533-6430
F: +1 613-533-2941

SAE NOTIFICATION

Report to the CCTG Central Office by telephone and/or fax within 24 hours of the event.
SAEs must be reported on the Serious Adverse Event Form.

Tel: 613-533-4630 Fax: 613-533-2941

CONTENTS

GENERAL INFORMATION II

INFORMATION FOR MRC INVESTIGATORS IV

INFORMATION FOR CCTG INVESTIGATORS V

CONTENTS 1

ABBREVIATIONS AND GLOSSARY 5

TRIAL SUMMARY 7

1 BACKGROUND 14
1.1 INTRODUCTION 14
1.2 RATIONALE AND OBJECTIVES 14
1.3 THE CASE FOR A TRIAL OF IMMEDIATE VERSUS EARLY SALVAGE TREATMENT AFTER RADICAL PROSTATECTOMY 14
1.4 THE CASE FOR A TRIAL OF HORMONE THERAPY DURATION IN MEN RECEIVING RADIOTHERAPY POST-PROSTATECTOMY 15
1.5 OTHER ONGOING RELEVANT STUDIES AND TRIALS 17
1.5.1 RADICALS-RT 17
1.5.2 RADICALS-HD 17
1.5.3 Other trials 17
1.6 RISKS AND BENEFITS 18
1.7 CONCLUSIONS 18

3 SELECTION OF CENTRES AND CLINICIANS 19

2 SELECTION OF PATIENTS 20
2.1 INVESTIGATIONS PRIOR TO EACH RANDOMISATION 21

3 RANDOMISATION & ENROLMENT 22
3.1 TRIAL RANDOMISATION OPTIONS 22
3.1.1 RADICALS-RT: Radiotherapy Timing Randomisation 22
3.1.2 RADICALS-HD: Hormone Duration Randomisation 22
3.2 RANDOMISATION CONTACTS 22
3.3 CO-ENROLMENT GUIDELINES 22

4 TREATMENT OF PATIENTS 23
4.1 GUIDANCE FOR RADICALS-RT 23
4.1.1 Early post-op radiotherapy 23
4.1.2 Deferred radiotherapy 23
4.1.3 Radiotherapy technique 23
4.2 GUIDANCE FOR RADICALS-HD 26
4.2.1 RT alone 26
4.2.2 Short-term hormone therapy plus RT 27
4.3 PROTOCOL TREATMENT DISCONTINUATION 27

4.4 ACCOUNTABILITY AND UNUSED DRUGS 28
4.5 MEASURES OF COMPLIANCE AND ADHERENCE 28
4.6 NON-TRIAL TREATMENT 28
4.6.1 Medications permitted/not permitted 28
4.6.2 Data on concomitant medication 28
4.7 CO-ENROLMENT GUIDELINES 28

5 ASSESSMENTS AND FOLLOW-UP 29
5.1 CASE REPORT FORM TIMINGS 29
5.2 PROCEDURES FOR ASSESSING EFFICACY 29
5.2.1 PSA measurements 29
5.2.2 Efficacy parameters 29
5.3 PROCEDURES FOR ASSESSING SAFETY 30
5.4 OTHER ASSESSMENTS 30
5.4.1 Patient reported outcomes 30
5.4.2 Health economics 30
5.5 EARLY STOPPING OF FOLLOW-UP 30
5.6 LOSS TO FOLLOW-UP 30
5.7 PATIENT TRANSFERS 31
5.7.1 Mortality data from Electronic Health Records 31
5.8 END OF TRIAL 31

6 STATISTICAL CONSIDERATIONS 33
6.1 METHOD OF RANDOMISATION 33
6.2 OUTCOME MEASURES 33
6.2.1 Primary outcome measure - RADICALS-RT 33
6.2.2 Primary outcome measure – RADICALS-HD 33
6.2.3 Secondary outcome measures 34
6.2.4 Early reporting of biochemical outcomes 34
6.3 SAMPLE SIZE 34
6.3.1 Basic assumptions 34
6.3.2 RADICALS-RT sample size 35
6.3.3 RADICALS-HD sample size 35
6.3.4 Overall sample size 37
6.3.5 Pilot phase 37
6.4 INTERIM MONITORING AND ANALYSES 37
6.5 STATISTICAL ANALYSIS PLAN 38
6.6 INDIVIDUAL PATIENT DATA META-ANALYSIS 38
6.6.1 RADICALS-RT 38
6.6.2 RADICALS-HD 38

7 QUALITY ASSURANCE AND CONTROL 39
7.1 COMPLIANCE 39
7.2 RADIOTHERAPY QUALITY ASSURANCE 39
7.3 MONITORING AND AUDIT 39
7.4 SOURCE DATA 39
7.4.1 Data Handling 40
7.5 PROTOCOL DEVIATIONS 40

8 SAFETY REPORTING ... 41
8.1 DEFINITIONS ... 41
8.2 CLARIFICATIONS ... 41
8.2.1 Medicinal Products ... 42
8.2.2 Adverse Events ... 42
8.3 DISEASE RELATED EVENTS AND ELECTIVE HOSPITALISATION ... 42
8.3.1 Institution Responsibilities ... 42
8.3.2 Investigator Responsibilities ... 43
8.3.3 Investigator Assessment ... 43
8.4 CTU RESPONSIBILITIES ... 45

9 ETHICAL CONSIDERATIONS ... 47

10 ANCILLARY STUDIES ... 48
10.1 PATIENT-REPORTED OUTCOMES ... 48
10.2 HEALTH ECONOMICS ... 48
10.3 TRANSLATIONAL RESEARCH ... 48

11 APPROVALS AND INDEMNITY ... 49
11.1 ETHICS APPROVALS ... 49
11.2 REGULATORY APPROVAL ... 49
11.3 INDEMNITY ... 49

12 TRIAL COMMITTEES ... 50
12.1 TRIAL MANAGEMENT GROUP (TMG) ... 50
12.2 TRIAL STEERING COMMITTEE (TSC) ... 50
12.3 INDEPENDENT DATA MONITORING COMMITTEE (IDMC) ... 50
12.4 ENDPOINT REVIEW COMMITTEE (ERC) ... 50
12.5 QUALITY OF LIFE SUBGROUP ... 50
12.6 RADIOTHERAPY QUALITY ASSURANCE SUBGROUP ... 50
12.7 PATHOLOGY QUALITY ASSURANCE SUBGROUP ... 50
12.8 TRANSLATIONAL STUDIES GROUP ... 51

13 PUBLICATION AND DISSEMINATION OF RESULTS ... 52

14 PROTOCOL AMENDMENTS ... 53
14.1 PROTOCOL ... 53
14.1.1 Amendments made to Protocol version 1.0 March 2007 ... 53
14.1.2 Amendments made to Protocol version 1.1 June 2007 ... 53
14.1.3 Amendments made to Protocol version 2.0 January 2008 ... 53
14.1.4 Amendments made to Protocol version 2.2 December 2008 ... 54
14.1.5 Amendments made to Protocol version 3.0 October 2009 ... 54
14.1.6 Amendments made to Protocol version 4.0 June 2011 ... 56
14.1.7 Amendments made to Protocol Version 5.0 March 2014 ... 56
14.1.8 Amendments made to Protocol Version 6.0 December 2018 ... 57
14.2 APPENDICES ... 58
14.2.1 Amendments made to Appendix A version 1.0 March 2007 ... 58
14.2.2 Amendments made to Appendix B version 1.0 March 2007 ... 58
14.2.3 Amendments made to Appendix A version 1.0 June 2007 ... 59

14.2.4 Amendments made to Appendix B version 1.1 June 2007 59
14.2.5 Amendments made to Appendix A version 2.0 January 2008 59
14.2.6 Amendments made to Appendix A version 2.2 December 2008 59
14.2.7 Amendments made to Appendix B version 2.1 December 2008 59
14.2.8 Amendments made to Appendix A version 3.0 October 2009...59
14.2.9 Amendments made to Appendix B version 3.0 October 2009...59
14.2.10 Amendments made to Appendix A version 4.0 June 2011 60
14.2.11 Amendments made to Appendices version 5.0 March 2014 60

15 REFERENCES.. 61

ABBREVIATIONS AND GLOSSARY

Abbreviation	Expansion
AD	Androgen Deprivation
AE	Adverse event
AP	Anterior/Posterior
AR	Adverse reaction
ARO	Academic Radiation Oncology
BAUS	British Association of Urological Surgeons
CCTG	Canadian Cancer Trials Group
CF	Consent form
CI	Chief Investigator
CI	Confidence Interval
CRF	Case Report Form
CT	Computerised Tomography
CTA	Clinical Trials Authorisation
CTAAC	Clinical Trials Awards and Advisory Committee
CTCAE	Common Terminology Criteria for Adverse Events
CTG	Clinical Trials Group
CTU	Clinical Trials Unit
CTV	Clinical Target Volume
DCF	Data Clarification Form
DMC	Data Monitoring Committee
DSS	Disease Specific Survival
EORTC	European Organisation for Research and Treatment of Cancer
EPC	Early Prostate Cancer
ERC	Endpoint Review Committee
EU	European Union
EudraCT	European Union Drug Regulatory Agency Clinical Trial
FBC	Full Blood Count
FFDM	Freedom from distant metastasis
FFTF	Freedom From Treatment Failure
GCP	Good Clinical Practice
GnRHa	Gonadotrophin releasing hormone analogue
GRO	General Registrar's Office
GS	Gleason Score
HE	Health Economics
HR	Hazard Ratio
HT	Hormone Therapy
IB	Investigator's Brochure
ICH	International Conference of Harmonisation
IDMC	Independent Data Monitoring Committee
IMP	Investigational Medicinal Products
IRB	Institutional Review Board
ISRCTN	International standard randomised controlled trial number
JCOG	Japanese Clinical Oncology Group
LHRH	Luteinising Hormone-Releasing Hormone

LR	Left/Right
LREC	Local Research Ethics Committee
LTHT	Long Term Hormone Therapy
MFS	Metastasis free survival
MHRA	Medicines and Healthcare Regulatory Authority
MLC	Multi-leaf Collimation
MRC	Medical Research Council
MRI	Magnetic Resonance Imaging
NCIC CTG	NCIC Clinical Trials Group
NCRI	National Cancer Research Institute
NCRN	National Cancer Research Network
NHS	National Health Service
NHSCR	National Health Service Central Register
ONS<br>PFS	Office for National Statistics<br>Progression Free Survival
PI	Principal Investigator
PIS	Patient information Sheet
PSA	Prostate Specific Antigen
PTV	Planning Target Volume
QA	Quality Assurance
QL	Quality of life
RADICALS	Radiotherapy and Androgen Deprivation In Combination After Local Surgery
RADICALS-RT	RADICALS Radiotherapy Timing Randomisation
RADICALS-HD<br>RCT	RADICALS Hormone Duration Randomisation<br>Randomised Controlled Trial
RP	Radical Prostatectomy
RT	Radiotherapy
RTOG	Radiation Therapy Oncology Group
SAE	Serious adverse event
SAR	Serious adverse reaction
SF12	Short Form 12
SI	Superior/Inferior
SmPC	Summary of Product Characteristics
SOP	Standard operating procedures
SPC	Summary of product characteristics
SSA	Site specific assessment
STHT	Short Term Hormone Therapy
SUSAR	Suspected unexpected serious adverse reaction
SV	Seminal Vesicle
SWOG	South West Oncology Group
tds<br>TMG	Three times daily<br>Trial Management Group
TSC	Trial Steering Committee
TROG	Trans-Tasman Radiation Oncology Group
UAR	Unexpected adverse reaction

TRIAL SUMMARY

SUMMARY INFORMATION TYPE	SUMMARY DETAILS
**[Acronym or short title]**	RADICALS
**Long Title of Trial**	Radiotherapy and Androgen Deprivation in Combination After Local Surgery: A randomised controlled trial in prostate cancer
**Version**	7.0
**Date**	17th February 2020
**MRC CTU at UCL ID**	PR10
**ISRCTN #**	40814031
**NCT #**	NCT00541047
**EudraCT #**	2006-000205-34
**CTA #**	20363/0402/001-0031
**MREC #**	07/Q0501/48
**Study Design**	RADICALS is an international, multi-centre, open-labelled, randomised controlled trial in prostate cancer. It is a trial with two separate randomisations for overlapping patient groups.<br><br>One randomisation was performed within 22 weeks after radical prostatectomy (**RADICALS-RT** or the **Radiotherapy Timing Randomisation**; see section 4 for eligibility criteria). In this, patients were randomised between early post-operative radiotherapy and deferred post-operative radiotherapy (for PSA failure).<br><br>The other randomisation was performed shortly before the administration of post-operative radiotherapy and concerns the addition of hormone therapy (**RADICALS-HD** or the **Hormone Duration Randomisation**). In this, patients were randomised between radiotherapy with no hormone therapy, radiotherapy with short-term hormone therapy or radiotherapy with long-term hormone therapy. Randomisation between all three arms was encouraged but patients could be randomised just between short-term and long-term hormone therapy or just between short-term hormone therapy and no hormone therapy.<br><br>Patients joining the RADICALS-RT were encouraged to join RADICALS-HD (if and when they had RT) but were not required to do so. Patients were required to consent separately to each randomisation. Patients who had not taken part in RADICALS-RT could still enter RADICALS-HD alone if post-operative radiotherapy was clinically indicated, either early post-surgery or in the deferred setting for PSA failure.
**Setting**	Participants were recruited from hospitals in the UK, Canada, Denmark and Ireland.

SUMMARY INFORMATION TYPE	SUMMARY DETAILS
**Type of Participants to be Studied**	Patients with non-metastatic adenocarcinoma of the prostate who have had a radical prostatectomy were eligible for RADICALS. Patients at increased risk of post-operative recurrence (see section 4) were eligible for RADICALS-RT. Patients who were due to receive post-operative RT were eligible for the Hormone Duration Randomisation.
**Ancillary Studies/Substudies**	Tissue samples were collected and will be considered for future translational projects, subject to funding applications.
**Sponsor**	University College London (previously MRC)
**Legal Representative in Europe**	UCL Research Limited, Dublin, Ireland
**Interventions to be Compared**	There are two interventions, radiotherapy and hormone therapy. See **Figures 1, 2 and 3.**
**Study Hypothesis**	RADICALS-RT: That the use of early post-operative radiotherapy would improve long-term clinical outcomes after radical prostatectomy<br><br>RADICALS-HD: That the use of hormone therapy would improve long-term clinical outcomes in men receving post-operative radiotherapy after radical prostatectomy, and that long-term hormone therapy would be more effective than short-term hormone therapy
**Primary Outcome Measure(s)**	RADICALS-RT:<br>Freedom-from-distant-metastasis (FFDM)<br><br>RADICALS-HD:<br>Metastasis-free-survival (MFS)
**Secondary Outcome Measure(s)**	• Disease-specific survival (DSS) (RADICALS-RT only)<br>• Freedom from treatment failure: PSA progression when on androgen deprivation<br>• Freedom from distant metastasis (FFDM) (HD only)<br>• Clinical progression-free survival (PFS): Clinical progression of prostate cancer or initiation of non-protocol hormone therapy or death from prostate cancer.<br>• Overall survival (OS): Death from any cause<br>• Non-protocol hormone therapy: Initiation of hormone therapy other than that randomised<br>• Treatment toxicity: Incidence of severe toxicity or serious adverse events. Radiotherapy treatment planning data is required to understand the cause of treatment toxicity.<br>• Patient reported outcomes: See Section 13.1 for details<br>• Freedom from biochemical progression: Where a biochemical progression event is definied as a PSA level of ≥0.4ng/ml following radiotherapy or a PSA level of > 2.0ng/ml regardless of prior radiotherapy.
**Randomisation**	1:1 (RT) +/- 1:1:1 (HD)

Summary Information Type	Summary Details
**Number of Participants to be Studied**	A total of 1396 patients were recruited into RADICALS-RT and 2840 patients into the RADICALS-HD. Many patients contributed to both randomisations. Patients were permitted to be randomised between two of the three arms in RADICALS-HD. For more details refer to **Section 9**. The trial completed recruitment on 30th December 2016.
**Duration**	Trial outcomes for RADICALS-RT and HD are planned to be assessed in 2022.
**Funder**	CRUK and MRC (UK)<br>CCTG (Canada)
**Chief Investigator**	Professor Chris Parker

TRIAL SCHEMA

RADICALS-RT: Radiotherapy Timing Randomisation

See **Figure 1**

- Early post-operative RT to prostate bed
- Deferred RT: RT to prostate bed given in the event of PSA failure.

The radiotherapy to be used is defined in the protocol by the RADICALS Radiotherapy Subgroup. It will use standard techniques and the dose-fractionation schedules will be 66 Gy in 33 fractions over 6.5 weeks or 52.5Gy in 20 fractions over 4 weeks. For more details refer to **Section 6.1**.

Figure 1: RADICALS-RT

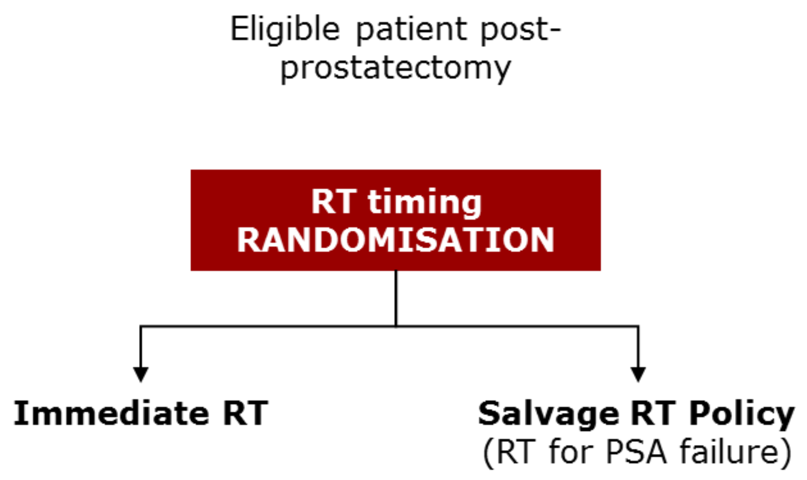

RADICALS-HD: Hormone Duration Randomisation

See **Figure 2**

- No hormone therapy with RT
- Short-term hormone therapy (6 months) commencing shortly before RT
- Long-term hormone therapy (24 months) commencing shortly before RT

Hormone therapy may be either LHRH agonist or bicalutamide 150mg daily. For more details refer to **Section 6.2**. For Canadian patients, hormonal therapy will consist of LHRH analogue therapy (in addition to antiandrogen for tumour flare, if desired) as bicalutamide monotherapy is not approved for use in Canada.

It is preferable to randomise patients between all three arms in RADICALS-HD but it is permissible to randomise patients between two of the three arms (see **Figure 3**). Patients can be randomised between:

- RT + no hormone therapy vs RT + 6m hormone therapy vs RT + 24m hormone therapy
- RT + no hormone therapy vs RT + 6m hormone therapy
- RT + 6m hormone therapy vs RT + 24m hormone therapy

Figure 2: Hormone Duration Randomisation

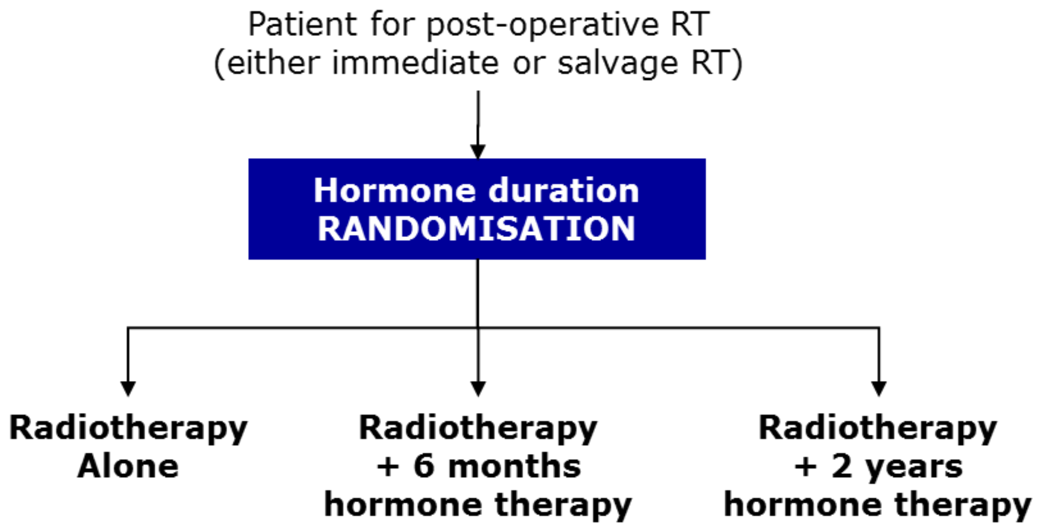

Figure 3: Two- and three-arm Hormone Duration Randomisations

Three-arm randomisation
(preferable)

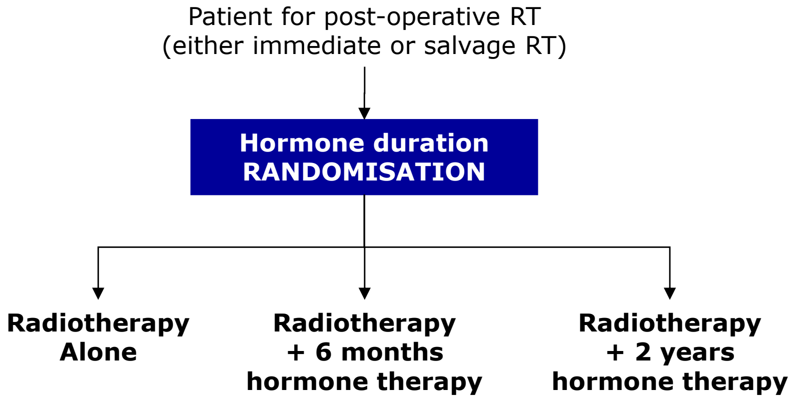

Two-arm randomisation
(none vs _short)

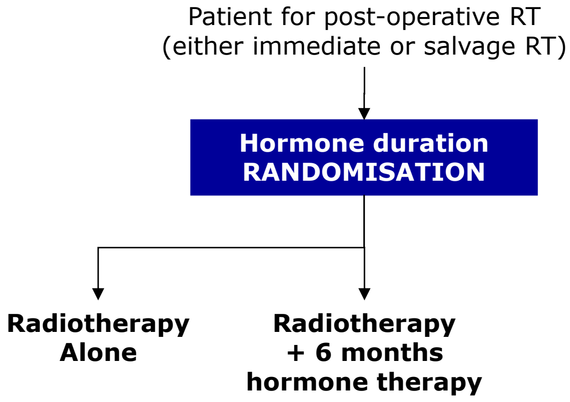

Two-arm randomisation
(short vs long)

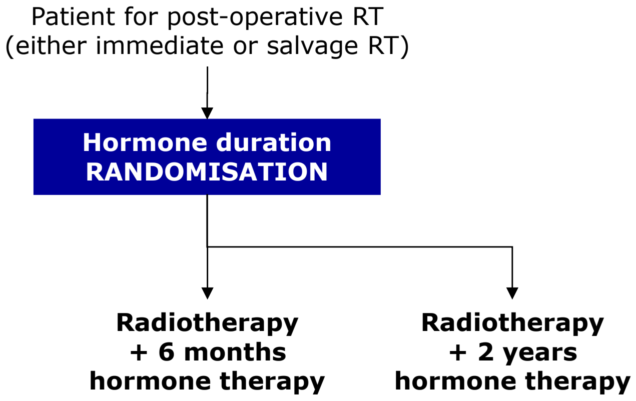

OUTCOME MEASURES

RADICALS-RT

Primary: Freedom from distant metastasis (any distant metastasis or prostate cancer death)

Secondary: Disease-specific survival (i.e. death due to prostate cancer)
Freedom from treatment failure
Clinical progression-free survival
Overall survival
Non-protocol hormone therapy
Treatment toxicity
Patient reported outcomes
Freedom from biochemical progression

Artistic Meta-analysis of RADICALS-RT

The proposed outcomes of interest for the ARTISTIC meta-analysis (see also sections 2.5.1 and 9.6.1) are as follows:

Event free survival
Time to start of salvage hormone therapy
Time free of metastasis
Prostate cancer specific survival
Overall survival

RADICALS-HD

Primary: Metastasis free survival (any distant metastasis or death) - please note the change from disease-specific survival.

Secondary: Freedom from distant metastasis
Freedom from treatment failure
Clinical progression-free survival
Overall survival
Non-protocol hormone therapy
Treatment toxicity
Patient reported outcomes

TRIAL DURATION

The trial was originally planned to address these questions over 12-13 years with 5½ to 6½ years of accrual and around 7 years of further Follow-up. The IDMC has advised that overall event rates have been much lower than anticipated. This prompted a change of primary outcome in the RADICALS-RT comparison (changed in protocol V4.0 from disease-specific survival to freedom from distant metastasis). The IDMC subsequently advised that event rates are lower still (without reference to comparative data by arm).

Following updated projections in 2019, the IDMC and TSC also supported the proposal for the change in primary outcome for RADICALS-HD from disease-specific survival to metastasis free survival, based on the findings from the IcECaP study. This proposal was also approved by the trial funder.

The patient group is men with non-metastatic adenocarcinoma of the prostate who have had a radical prostatectomy. Treatment duration within the trial will range from zero months (i.e. a proportion of patients, maybe 60%, allocated to deferred radiotherapy will never need it) to 24 months (for patients allocated long-term hormone therapy). Follow-up is required every 4 months for 2 years, every 6 months from 2 to 5 years and then annually. This broad patient group has a good long-term prognosis and full, long-term Follow-up data are essential to understand the impact of these treatments. For more details refer to **Section 7.1**.

ANCILLARY STUDIES/SUBSTUDIES

Patient reported outcomes will be collected to assess sexual function, urinary function, bowel function and general quality of life throughout the course of the trial. For more details refer to **Section 13.1**.

Health economics will be assessed by patient reported questionnaire. For more details refer to **Section 13.2**.

The trial is planned to collect prostatectomy specimens for future translational studies in order to identify and validate novel biomarkers of disease recurrence. For more details refer to **Section 13.3**. The protocol will be amended appropriately to reflect any changes regarding translational studies or a substudy protocol will be developed.

1 BACKGROUND

1.1 INTRODUCTION

Prostate cancer is the commonest cancer in UK men, with an incidence in 2008 of 37,000 cases (1). Radical prostatectomy is a standard of care for men presenting with localised disease. Conventional practice following surgery has been observation, with additional treatment, such as radiotherapy (RT) or hormone therapy (HT), used in the salvage setting for those who develop recurrent disease. The routine use of post-operative adjuvant therapy has shown benefits for other cancer types, such as breast and colorectal cancer, and is sometimes used in prostate cancer, but has not been well studied. Large randomised trials are needed to evaluate the role of adjuvant therapy following radical prostatectomy.

1.2 RATIONALE AND OBJECTIVES

Radical prostatectomy is a common operation. Hospital Episodes Statistics report 4,904 such operations were performed in England in 2010 (2). This is a significant under-estimate because it excludes operations performed outside the NHS. If rates of PSA testing in the UK continue to increase (3), then both the incidence of diagnosed prostate cancer, and the proportion of patients presenting with localised disease, will also rise. Thus, the number of radical prostatectomies performed each year in the UK is set to increase. According to the Institute for Clinical Evaluative Sciences, the number of radical prostatectomies per year in Canada is estimated to be between 5000 and 7000 (4).

Although the number of radical prostatectomies being performed is increasing there is considerable uncertainty over the optimal management strategy for patients that have had a prostatectomy. The two main management questions relate to the timing of radiotherapy and the use of hormone therapy in conjunction with post-operative radiotherapy (5-7). RADICALS will address both of these questions.

1.3 THE CASE FOR A TRIAL OF IMMEDIATE VERSUS EARLY SALVAGE TREATMENT AFTER RADICAL PROSTATECTOMY

There are three randomised controlled trials of adjuvant radiotherapy to the prostate bed published to date. EORTC 22911 recruited 1005 patients with pT3 disease post-radical prostatectomy, who were randomised between observation and adjuvant RT (8-9). A statistically significant advantage was seen for adjuvant radiotherapy in terms of biochemical progression-free survival (hazard ratio (HR) 0.49, 95.3% CI 0.41 – 0.59; $p<0.0001$) with 61% and 41% event-free at 10 years. An advantage was also reported for adjuvant radiotherapy in terms of clinical progression-free survival (HR 0.81, 95% CI 0.65 – 1.01; $p=0.054$) with 70.3% and 64.8% PFS event-free at 10 years. However, there was no evidence of a difference in overall survival ($p>0.1$) with 10-year survival rates of 76.9% with adjuvant RT and 80.7% with observation.

The second randomised controlled trial, SWOG 8794 (CCTG PR-2) had a similar design: 425 men with pT3 disease were randomised to either observation or adjuvant radiotherapy to the prostate bed, with median follow-up at the time of analysis of 10.6 years (10). Once again, adjuvant radiotherapy was associated with a statistically significant improvement in biochemical control (HR 0.43 95% CI 0.31, 0.58, $p<0.001$). At 15 years, there was a statistically significant advantage for adjuvant radiotherapy for metastasis-free survival (HR 0.74 95% CI 0.57, 1.00, $p=0.053$), and overall survival.

The third trial, the German Radiotherapy Group trial ARO 96-02, randomised 307 men with pT3 disease to either observation or adjuvant RT to the prostate bed (11). We note that 20% of patients never received their allocated RT. At a median follow-up of 3.3 years, analysis by treatment received, rather than by intention to treat, found that adjuvant radiotherapy was associated with improved biochemical control (81% vs 60% event-free at 4 years, HR 0.4, p<0.0001). These early results are consistent with those of EORTC 22911 and SWOG 8794, but AR0 96-02 was not sufficiently powered to address the effect of adjuvant treatment on clinical outcomes such as survival.

Standard practice following radical prostatectomy has evolved since the SWOG 8794 and EORTC 22911 trials were designed in the mid-1980s. In particular, the routine use of sensitive PSA assays means that contemporary patients with an undetectable post-operative PSA level have a lower risk of relapse than in the past and so less scope to benefit from adjuvant treatment. In addition, post-operative biochemical relapse can be detected earlier than clinical relapse was previously, and early detection may lead to an improvement in the efficacy of salvage RT. For both of these reasons, the benefits seen for adjuvant RT in SWOG 8794 and EORTC 22911 should not lead to the general acceptance of treatment in the adjuvant setting. Instead, the results provide a strong rationale for a comparison between adjuvant treatment and the current standard of care, which is observation with early salvage treatment for biochemical failure.

There is no consensus among UK oncologists on whether to use adjuvant or early selective salvage radiotherapy. A survey of 49 UK urological oncologists found that 25 (51%) did, and 24 (49%) did not recommend adjuvant RT for pT3 margin-positive cases (12). In a second survey of 188 UK Oncologists and Urologists there was widespread uncertainty regarding the use both of adjuvant radiotherapy and the mode, timing and duration of hormone therapy (13). This finding highlights the need for randomised studies addressing this issue. In designing the RADICALS trial, another survey was completed by 102 UK and Canadian urologists and oncologists. The responses reported clinicians offering adjuvant radiotherapy to between 0% and 30% of their post-operative patients with a median offering adjuvant radiotherapy to 3% of post-operative patients.

1.4 THE CASE FOR A TRIAL OF HORMONE THERAPY DURATION IN MEN RECEIVING RADIOTHERAPY POST-PROSTATECTOMY

Several randomised trials have demonstrated that the addition of hormone therapy improves overall survival in men receiving primary radiotherapy for prostate cancer e.g. EORTC 22863 (14), RTOG 86-10 (15), RTOG 85-31 (16), and a trial from Boston (17). However, until recently there were no reported randomised controlled trials addressing the role of hormone therapy in men receiving post-operative radiotherapy.

Three retrospective non-randomised studies have compared the outcome of salvage RT alone versus salvage RT plus short-term (4-6 months) hormone therapy, and have observed improved biochemical control rates with the addition of hormone therapy (18-20).

In 2010, the first results from a randomised trial in this setting, RTOG 96-01, were presented at the ASTRO annual conference (21). The trial recruited 771 patients with PSA failure after radical prostatectomy and randomised them between early salvage RT alone versus early salvage RT plus 2 years of hormone therapy with bicalutamide 150mg daily, with overall survival as the main outcome measure. The overall survival data are immature, but an advantage for adjuvant bicalutamide was reported in terms of freedom from distant metastasis at 7 years (93% vs 87%, p<0.04). The RTOG 96-01 trial (21) does not provide information on the use of short-term hormone therapy.

A further trial, RTOG 85-31 randomised patients with locally advanced prostate cancer between RT alone versus RT plus long-term hormone therapy. Just 139 of the 977 patients in this trial had

previously had a radical prostatectomy, and there are no published data concerning the outcome of this subgroup (16;22).

The Early Prostate Cancer (EPC) Program accrued 4400 men who had radical prostatectomy and were randomised between observation versus 2 years adjuvant bicalutamide 150 mg. At a median follow-up of 7.4 years, there was no evidence of a difference in overall survival, but the data remain immature in this predominantly low-risk population. This very large trial serves to underline that at present there is no proven role for adjuvant hormone therapy alone after radical prostatectomy (23-24). It is important to note that this trial will not answer the questions RADICALS is posing, since the indications for post-operative radiotherapy were not specified, and because salvage treatment was delayed until clinical (rather than biochemical) progression was observed.

A small trial of adjuvant hormone therapy in men with pathologically involved pelvic lymph nodes, EST-3886, was stopped after 98 patients had been accrued because an overall survival advantage for adjuvant treatment was observed (25-26).

One question that has received little or no attention to date is that of the optimum duration of hormone therapy in patients receiving post-operative radiotherapy to the prostate bed. The surveys of UK urological and oncological opinion (12-13), mentioned above, found that the use of hormone therapy was variable, with most urologists recommending radiotherapy alone. Only 35% of urologists and 34% of oncologists were using hormone therapy in combination with radiotherapy. Among those oncologists who used hormone therapy in combination with post-operative RT, short-term hormone therapy (defined as 3 to 12 months) was recommended by 31% of respondents, long-term hormone therapy (defined as >12 months) by 25%, while the remaining 44% used both short-term and long-term hormone therapy depending on the characteristics of the patient. Similarly, in the survey conducted in planning the RADICALS trial, respondents were using none, short-term and long-term hormone therapy for a median (quartiles) of 50% (0%, 90%), 0% (0%, 50%) and 10% (0%, 33%) of their patients receiving adjuvant post-operative radiotherapy and 50% (0%, 90%), 13% (0%, 50%) and 5% (0%, 30%) of their patients receiving salvage post-operative radiotherapy i.e. there is significant variation in clinical practice.

In the context of primary (i.e. not post-operative) RT for prostate cancer, the appropriate duration of hormone therapy was addressed by RTOG 92-02 (27), which randomised 1554 men receiving RT for locally advanced disease between short-term hormone therapy (4 months) and long-term hormone therapy (28 months). Long-term hormone therapy was associated with improved 5-year cause-specific survival (95% vs 91%, p=0.006), with no evidence to date of a significant difference in overall survival at 5 years (80% vs 79%, p=0.73). Similarly, results were reported from TROG 96-01, the largest randomised trial to date addressing the role of neoadjuvant androgen deprivation prior to RT in predominantly high risk non-metastatic disease (28). The use of 6 months neoadjuvant androgen deprivation reduced the risk of distant progression (HR 0.49, 95% CI 0.31-0.76, p=0.001) and death from any cause (HR 0.63, 95% CI 0.48-0.83, p=0.0008). The use of 3 months neoadjuvant androgen deprivation did not provide good evidence of a benefit.

The current pattern of UK practice, with no consensus regarding the need for, or duration of, hormone therapy in men receiving post-operative RT, combined with the increasing popularity of radical prostatectomy, provides a strong rationale for a phase III study. RADICALS-HD will investigate the question of RT alone versus RT plus short-term hormone therapy versus RT plus long-term hormone therapy in this setting. The duration of short-term hormone therapy will be 6 months, based on TROG 96-01 (28), and long-term hormone therapy will be for 2 years, based on RTOG 92-02 (27) and RTOG 96-01 (21). Long-term results from RTOG 96-01 were presented at conferences in 2010 and showed favourable outcomes for 2 years ADT over no ADT.

1.5 OTHER ONGOING RELEVANT STUDIES AND TRIALS

1.5.1 RADICALS-RT

There are two other trials asking complementary questions to RADICALS-RT which were active in Apr-2011. These are the TROG 08.03 RAVES and FNCLCC-GETUG-17/0702 trial, both of which randomise patients between immediate post-op RT and salvage RT for PSA failure. ADT is not given with RT in RAVES whereas 6m ADT is given with RT in GETUG-17. There are differences between these trials and RADICALS-RT, notably the earlier primary outcome measures, but the primary design is sufficiently complementary that combined analyses are planned for long-term outcome measures. This meta-analysis may be performed prior to the main outcome measure analysis of RADICALS-RT, as detailed in section 9.6. The meta-analysis will be based on up-to-date data from all trials. All RADICALS-RT randomised patients will be included in the analyses, which will be performed on an intention-to-treat basis. The meta-analysis will be conducted in stages, as data for each outcome matures, with analyses being carried out when reasonable power to detect treatment differences is achieved. Relevant information, for example accrual and follow-up durations, control arm event rates and patient retention rates, will be sought from each study to finalise the appropriate time point for the analysis of each outcome. Full details will be documented in the ARTISTIC protocol and statistical analysis plan.

1.5.2 RADICALS-HD

There are also complementary trials to RADICALS-HD. The EORTC 22043-30041 trial of adjuvant treatment after radical prostatectomy randomises patients between adjuvant radiotherapy alone versus adjuvant radiotherapy plus 12 months of hormone therapy. Combined analyses are planned in the future.

In the early salvage setting, the FNCLCC-GETUG-16/0504 trial compares RT alone versus RT plus 6 months ADT, and the RTOG 05-34 SPPORT trial compares early salvage RT to the prostate bed alone against supplementing this with 4 to 6 months ADT or 4 to 6 months ADT and pelvic RT.

In addition to the trials listed above, it is also pertinent to consider RTOG P-0011, which was originally designed as a 3-arm trial in men at high risk of recurrence following radical prostatectomy, with the randomisation between adjuvant RT, long-term adjuvant hormone therapy (24 months) or RT plus long-term hormone therapy. The trial was modified to a 2-arm trial comparing adjuvant RT +/- hormone therapy to improve recruitment although the trial closed early. The current UK standard of care, namely observation with salvage treatment in the event of PSA failure, was not included in the trial. Similarly, a German study known as AP 26/99 and ARO 00/01 planned to randomise around 900 patients with isolated PSA relapse to early salvage radiotherapy with or without short-term HT (6 months) (29). The trial closed in 2003 because of poor accrual.

Completion of recruitment to RADICALS will be facilitated in the UK by favourable attitudes of UK urologists towards radiotherapy, the centralisation of radical prostate cancer surgery in Cancer Centres, the established multidisciplinary team pattern of working, and the NCRN infrastructure.

1.5.3 OTHER TRIALS

In addition to the above trials which have completed recruitment of patients, we note that the Japanese Clinical Oncology Group are running a trial (JCOG 0401) for patients with isolated PSA failure after prostatectomy where patients are randomised to radiotherapy with hormone therapy or hormone therapy alone (30). The target is 200 patients. This trial does not address the key question of the timing of post-operative radiotherapy.

1.6 RISKS AND BENEFITS

The treatments described in this protocol reflect additional treatment given after surgery. Each of the management strategies tested in RADICALS are familiar in clinical practice, therefore the potential adverse effects, risks, hazards and benefits are similar to those which would be experienced in standard practice. It is intended that RADICALS can define the most appropriate strategy for this group of patients, addressing the balance of benefits against risks.

1.7 CONCLUSIONS

In summary, the three existing phase III trials of adjuvant RT against observation listed above (8-11) will be of very limited value in practice, either because they have been superseded by clinical developments or because they are too small. The paucity of randomised trials addressing the optimum duration of hormone therapy in the post-operative setting is an important omission. The popularity of radical prostatectomy, together with current oncological and urological opinion in the UK, Canada and elsewhere, presents an opportunity for a large, randomised trial addressing both the timing of post-operative treatment (early versus deferred) and the duration of hormone therapy (none versus short-term versus long-term) used in addition to prostate bed RT.

3 SELECTION OF CENTRES AND CLINICIANS

In order to participate in RADICALS, investigators and centres must be registered with one of the participating groups and must fulfil a set of basic criteria.

Each investigator must:

- Regularly undertake treatment of prostate cancer
- Have appropriate experience of conducting trials according to the principles of Good Clinical Practice (GCP)
- Comply with protocol treatment and follow-up schedule
- Maintain a local Trial Master File which will contain essential documents for the conduct of the trial

- Submit all trial data in a timely manner and as described in the protocol. Individual centres may be suspended on the recommendation of the Trial Management Group (TMG) if data returns are poor or if trial conduct is violated in other ways
- Notify the trials unit immediately of all Serious Adverse Events (SAEs). The initial SAE report must be promptly followed by detailed written reports
- Comply with Radiotherapy Quality Assurance
- Not disclose any trial data without the approval of the Trial Steering Committee (TSC)
- Retain all trial related documents for 15 years after completion of the trial

Each centre must:

- Conduct the trial in compliance with the principles of GCP and applicable regulatory requirements
- Have an adequate number of qualified staff and adequate facilities, for the foreseen duration of the trial, to conduct the trial properly and safely
- Must ensure that all staff assisting with the trial are adequately informed about the protocol and their trial related duties
- Obtain necessary local approvals
- Allow on-site monitoring
- Have PSA test with an assay sensitivity of 0.1ng/ml or lower

For information about additional site registration criteria, trial documentation and local procedures, see local **Appendix B**.

2 SELECTION OF PATIENTS

Patients with non-metastatic adenocarcinoma of the prostate who have had a radical prostatectomy will be eligible for RADICALS. **All patients must fulfil the main entry criteria and the criteria relevant to the randomisation they are taking part in.** Patients who are taking part in RADICALS-RT can also take part in RADICALS-HD when they have radiotherapy. Complete inclusion and exclusion criteria are listed in **Table 1**.

Table 1: Patient inclusion and exclusion criteria

TRIAL SECTION	INCLUSION	EXCLUSION
All Patients	• Patient has undergone radical prostatectomy<br>• Prostatic adenocarcinoma<br>• Written informed consent	• Bilateral orchidectomy<br>• Prior pelvic RT<br>• Other active malignancy likely to interfere with protocol treatment or follow-up<br>• Known distant metastases from prostate cancer<br>• Pre-operative hormone therapy within previous 6 months<br>• Previous pre-operative hormone therapy for longer than 8 months<br>• Any post-operative hormone therapy*
RADICALS-RT	• Post-operative PSA ≤0.2ng/ml<br>• Ideally more than 4 weeks and less than 22 weeks after radical prostatectomy#<br>• One or more of:<br>:: pT3/4<br>:: Gleason 7-10 (biopsy or surgical sample)<br>:: Pre-operative PSA≥10ng/ml<br>:: Positive margins	• Post-operative biochemical failure, defined as EITHER<br>:: two consecutive rises in PSA and final PSA >0.1ng/ml OR<br>:: three consecutive rises in PSA<br>• Ideally more than 22 weeks since radical prostatectomy#
RADICALS-HD	• Patient due to receive post-operative RT (early or deferred)	• PSA >5ng/ml at the time of randomisation

#Patients randomised to early RT ideally should start trial treatment within 26 weeks after radical prostatectomy

*Patients joining only the 6 months vs 2 years comparison in RADICALS-HD may begin post-operative hormone therapy prior to randomisation. However, this MUST be discussed with trials office before randomisation in this circumstance.

2.1 INVESTIGATIONS PRIOR TO EACH RANDOMISATION

All patients must have the following tests prior to randomisation according to the timings in the table below.

Table 2: Timing of Investigations

COMPARISON	TEST	TIMING
**RADICALS-RT**	Bone scan*	16 weeks prior to randomisation
	PSA**	Within 30 days prior to randomisation<br>AND<br>At least 30 days after surgery
**RADICALS-HD**	Bone scan#	16 weeks prior to randomisation
	PSA**	Within 30 days prior to randomisation<br>AND<br>At least 30 days after surgery

* Bone scan only required if Gleason score ≥8 and post-operative PSA is detectable. Additional investigations are at the clinician's discretion.

** PSA assay must have a sensitivity of 0.1ng/ml or lower

Bone scan only required if PSA>2. Additional investigations are at the clinician's discretion.

3 RANDOMISATION & ENROLMENT

3.1 TRIAL RANDOMISATION OPTIONS

RADICALS is an international, multi-centre, open-labelled, randomised controlled trial. Blinding of treatment allocation in the trial is impracticable and will not be used. RADICALS is a trial with two separate randomisations.

3.1.1 RADICALS-RT: Radiotherapy Timing Randomisation

This randomisation, ideally performed within 22 weeks after radical prostatectomy (RP), is defined as '**Radiotherapy Timing Randomisation**'. If the patient meets the eligibility criteria (see **Table 1** in Section **4** and **Appendix A V**), he may be randomised between early radiotherapy and deferred radiotherapy for PSA failure.

3.1.2 RADICALS-HD: Hormone Duration Randomisation

This randomisation, normally performed before the administration of post-operative radiotherapy, is defined as '**Hormone Duration Randomisation**'. This means that for patients receiving deferred RT, enrolment to RADICALS-HD may take place months or years after radical prostatectomy (see **Figures 1-3**). Patients entering into this randomisation may have already been in RADICALS-RT or not. Prior to their radiotherapy, patients may be randomised between the following 3 arms: no hormone therapy, short-term hormone therapy and long term hormone therapy. All patients taking part in RADICALS-HD may elect to be randomised between two, rather than all three, arms to facilitate comparisons and trial recruitment.

3.2 RANDOMISATION CONTACTS

Randomisation to RADICALS-RT closed in June 2015, randomisation for RADICALS-HD closed in December 2016.

3.3 CO-ENROLMENT GUIDELINES

Co-enrolment to other trials is permitted, providing this does not interfere with assessment of RADICALS outcome measures. See **Section 6.7** for further detail.

4 TREATMENT OF PATIENTS

4.1 GUIDANCE FOR RADICALS-RT

Patients in RADICALS-RT will be allocated to either early post-operative RT or deferred RT. RT will be given as 66Gy in 33 fractions over 6.5 weeks or 52.5Gy in 20 fractions over 4 weeks. Treatment with radiotherapy (or hormone treatment – see **Section 6.2**) will commence within 2 months after the randomisation for early RT patients or within 2 months after biochemical failure for deferred RT patients. For information on radiotherapy quality assurance see **Section 10.2**.

If radiotherapy centres wish to use IMRT, they may do so with the exception of Canadian centres who will need to be credentialed for IMRT before they can use it for their RADICALS patients (full details are available in the Canadian Appendix) . The prostate bed dose should be 66Gy in 2Gy fractions or 52.5Gy in 20 fractions. However, if it is intended to use IMRT to treat the pelvic lymph nodes in addition to the prostate bed, then treatment should be given over 33 fractions to a total nodal dose of 52-54Gy. Alternative schedules should be agreed with the RADICALS Trial Management Group.

4.1.1 EARLY POST-OP RADIOTHERAPY

Patients allocated to early radiotherapy to the prostate bed will start treatment within approximately 2 months of entering into RADICALS-RT and ideally within 26 weeks after surgery. Radiotherapy will be according to guidelines given in **Section 6.1.3**. Patients allocated early post-operative radiotherapy can also enter RADICALS-HD if they wish: this is encouraged. Alternatively, the use of hormones can be decided by the responsible investigator. Radiotherapy will be delayed by 2 months, up to 8 months after surgery, if the patient is due to receive hormone therapy.

4.1.2 DEFERRED RADIOTHERAPY

This is a monitoring policy, with deferred RT to prostate bed given in the event of biochemical failure. PSA will be tested at each follow-up visit (see **Section 7**) and more often if rising PSA is detected. Biochemical failure is defined as EITHER two consecutive rising PSA levels and a PSA of greater than 0.1 ng/ml OR three consecutive rising PSA levels. If post-operative biochemical failure is confirmed, patients will receive radiotherapy as described in **Section 6.1.3** and should be offered entry to the RADICALS-HD; this is encouraged. Radiotherapy will be delayed by 2 months if the patient is due to receive hormone therapy.

4.1.3 RADIOTHERAPY TECHNIQUE

4.1.3.A RADIATION THERAPY

Radiotherapy to start within approximately 2 months of randomisation. Treatment should be CT planned with the patient supine, with empty rectum and comfortably full bladder. Recommended doses are in **Section 6.1.3.D**.

4.1.3.B PHYSICAL FACTORS

Megavoltage equipment is required with effective photon energies >6MV. Minimum source-to-axis distance is 100cm. The treatment technique will typically be by a 3-field or 4-field coplanar technique with blocks or multi-leaf collimation (MLC) leaf positions designed for all fields to protect uninvolved structures. Intensity-Modulated Radiation Therapy (IMRT) techniques may be used, subject to the RADICALS Radiotherapy Quality Assurance (RTQA) reviewers' approval.

4.1.3.C TREATMENT VOLUMES GUIDANCE

Please note that the following treatment volumes are for guidance only.

[A] PROSTATE BED

Clinical Target Volume (CTV). The CTV will include the prostate bed in all patients. The pelvic lymph node regions may also be included at the investigator's discretion. Information which may be used to define the prostate bed CTV include:

i. Histopathologic information of prostate size and tumour extent to specific boundaries of the surgical resection
ii. Pre-operative imaging e.g. pelvic CT/MRI studies
iii. Post-operative anatomy on planning CT scan

The definition of the prostate bed CTV is based on the estimated location of the pre-operative prostate volume plus sites of possible microscopic tumour extension, plus the extent of the surgical bed, and should normally include any surgical clips provided that the normal-tissue dose-constraints are satisfied. The original volume of seminal vesicles (including any residual seminal vesicle tissue post-op) will not be considered target if they were not pathologically involved with tumour, and if the predicted pre-operative risk of seminal vesicle involvement was less than 15% using the Roach formula (% seminal vesicle (SV) involvement risk = PSA + 10 x [GS – 6] where GS=Gleason Score. If there was pathologic involvement of the seminal vesicles, or if the predicted risk of involvement was greater than 15%, then the seminal vesicles will be considered target.

:: **Low-risk** = <15% according to the Roach formula
:: **High-risk** = ≥15% according to the Roach formula

Inferior border: 5mm cranial to the superior border of the penile bulb

Anterior border: As follows:

i. Caudal (less than 2cm above anastamosis) – posterior aspect of symphysis pubis
ii. Cranial (more than 2cm above anastamosis) – posterior 1/3 of bladder wall

Posterior border: Anterior rectal wall

Lateral border: Medial border of obturator internus and levator ani muscles

Superior border: As follows:

i. If SV low-risk and pathologically uninvolved: base of SV
ii. If SV high risk or pathologically involved: tips of SV
iii. If SV absent, the superior border should be determined with reference to the estimated position of the pre-operative SV using the longitudinal dimension superiorly from urogenital diagphragm to reflect preoperative size of prostate, together with the position of any surgical clips.

Prostate bed – Planning Target Volume: The planning target volume (PTV) will add 1.0 cm in all directions, for day-to-day variation in set up and for CTV motion.

Prostate bed – Field size: The maximum unshaped field size in each axis (anterior/posterior (AP), left/right (LR) and superior/inferior (SI)) will typically be between 8.0 and 12.0cm.

[B] PELVIC LYMPH NODES

Clinical Target Volume: The CTV will include the prostate bed in all patients. The pelvic lymph node regions may also be included at the investigator's discretion. The pelvic nodal

CTV will include the internal iliac/obturator, external iliac, pre-sacral and pre-sciatic nodal regions.

Pelvic lymph nodes – Planning Target Volume:

- Inferior border: inferior border of prostate bed PTV
- Lateral borders: pelvic sidewalls
- Anterior border: posterior symphysis
- Posterior border: anterior S2-3 junction
- Superior border: lower 1/3 sacro-iliac (S-I) joints

Pelvic lymph nodes – field borders:
Determined by PTV above. Conformal blocks/MLC leaves may be used to shield inferior part of rectum and anus, the base of the penis, and the antero-superior part of the bladder.

4.1.3.D Radiation Doses

(A) PROSTATE BED

Radiotherapy will be given once a day, five sessions a week. The dose shall be prescribed at the intersection of the central rays of the beams. The prescribed dose to the intersection of the central rays of the beams is recommended to be one of the following:

- 66Gy given in 33 fractions over 6.5 weeks
- 52.5Gy given in 20 fractions over 4 weeks

Other schedules should be discussed and approved by the TMG.The minimal dose to the PTV shall not be less than 95% of the prescribed dose; the maximum, not more than 105% of the prescribed dose.

(B) PELVIC LYMPH NODES

Radiotherapy will be given once a day, five sessions a week. The dose shall be prescribed at the intersection of the central rays of the beams. The prescribed doses to the intersection of the central rays of the beams will be:

- 46Gy given in 23 fractions over 4.5 weeks

4.1.3.E CRITICAL NORMAL STRUCTURES

The dose-volume objectives are provided in **Tables 3** and **4**. These are for guidance only.

Table 3: Dose & Volume Objective: Daily fractions of 2 Gy

STRUCTURE	DOSE	VOLUME OBJECTIVE
**Bladder**	50 Gy	< 80%
	60 Gy	< 50%
**Rectum**	30 Gy	< 80%
	40 Gy	< 70%
	50 Gy	< 60%
	60 Gy	< 50%
	66 Gy	< 30%

Table 4: Dose & Volume Objective: 52.5Gy in 20 fractions over 4wks

STRUCTURE	ISODOSE	VOLUME OBJECTIVE
**Bladder**	40 Gy	< 80%
	48 Gy	< 50%
**Rectum**	24 Gy	< 80%
	32 Gy	< 70%
	40 Gy	< 60%
	48 Gy	< 50%
	52.5 Gy	< 30%

4.2 GUIDANCE FOR RADICALS-HD

4.2.1 RT ALONE

Patient would be treated with post-operative radiotherapy alone as described in **Section 6.1**. Radiotherapy should ideally start as soon as possible but within 2 months after randomisation.

4.2.2 SHORT-TERM HORMONE THERAPY PLUS RT

Trial treatment should ideally start as soon as possible but within 2 months after randomisation. Radiotherapy should commence approximately 2 months after starting hormone treatment. Treatment using a gonadotrophin releasing hormone analogue (GnRHa) should be given for 6 months. Because of the possibility of tumour 'flare', an anti-androgen (such as cyproterone acetate 100mg tds) should be used for one week prior to the first GnRHa administration, and continued for a total of 3 weeks. The choice of GnRHa may vary according to local practice (e.g. goserelin, leuprorelin), but in this arm the use of 3-month depot preparations should be avoided. Where possible, one month preparations (e.g. goserelin 3.6mg, leuprorelin 3.75mg) should be used in order to hasten testosterone recovery after the treatment period. Bicalutamide monotherapy 150mg daily or degarelix each for 6 months are acceptable alternatives. For Canadian patients, hormonal therapy will consist of LHRH analogue therapy (in addition to antiandrogen for tumour flare, if desired) because bicalutamide monotherapy is not approved for use.

4.2.2.A DISPENSING HORMONE THERAPY

Centres will use routinely available products (either LHRH agonists or bicalutamide monotherapy) that will be stored and dispensed in the usual way. For Canadian patients, hormonal therapy will consist of LHRH analogue therapy (in addition to antiandrogen for tumour flare, if desired) because bicalutamide monotherapy is not approved for use.

4.2.2.B LONG-TERM HORMONE THERAPY PLUS RT

Trial treatment should ideally start as soon as possible but within 2 months after randomisation. Radiotherapy should commence approximately 2 months after starting hormone treatment. Treatment using a gonadotrophin releasing hormone analogue (GnRHa) should be given for 24 months. Because of the possibility of tumour 'flare', an anti-androgen (such as cyproterone acetate 100mg tds) should be used for one week prior to the first GnRHa administration, and continued for a total of 3 weeks. The choice of GnRHa may vary according to local practice (e.g. goserelin, leuprorelin). In this arm, the use of 3-month depot preparations (e.g. goserelin 10.8mg, leuprorelin 11.25mg) is encouraged in the interests of patient convenience, but 1 month or 2 month depots are acceptable. Bicalutamide monotherapy 150mg daily, or degarelix, for 24 months are acceptable alternatives. In the case of bicalutamide, patients should be considered for prophylactic radiotherapy to bilateral breast buds (8Gy single fraction using orthovoltage radiation) to prevent painful gynaecomastia. For Canadian patients, hormonal therapy will consist of LHRH analogue therapy (in addition to antiandrogen for tumour flare, if desired) because bicalutamide monotherapy is not approved for use.

4.2.2.C DISPENSING HORMONE THERAPY:

Centres will use routinely available products (either LHRH agonists or bicalutamide monotherapy) that will be stored and dispensed in the usual way. For Canadian patients, hormonal therapy will consist of LHRH analogue therapy (in addition to antiandrogen for tumour flare, if desired) because bicalutamide monotherapy is not approved for use.

4.3 PROTOCOL TREATMENT DISCONTINUATION

In consenting to the trial, patients are consenting to trial treatment, trial follow-up and data collection. However, an individual patient may stop treatment early for any of the following reasons:

- Disease Progression
- Unacceptable toxicity or adverse event
- Intercurrent illness that prevents further treatment

- Any change in the patient's condition that justifies the discontinuation of treatment in the clinician's opinion
- Inadequate compliance with the protocol treatment in the judgement of the treating physician
- Withdrawal of consent for treatment by the patient

As the patient's participation in the trial is entirely voluntary, they may choose to discontinue the trial treatment at any time without penalty or loss of benefits to which they are otherwise entitled. Although the patient is not required to give a reason for discontinuing their trial treatment, a reasonable effort should be made to establish this reason while fully respecting the patient's rights.

Patients should remain in the trial for the purpose of follow-up and data analysis (unless the patient withdraws their consent for this). If a patient is withdrawn from follow-up, refer to **Section 6.5**.

4.4 ACCOUNTABILITY AND UNUSED DRUGS

As all drugs are licensed in the countries in which the trial will be performed, drug accountability measures will not be necessary. Drugs should be obtained as per local practice.

4.5 MEASURES OF COMPLIANCE AND ADHERENCE

Date of treatment, dose, delays and reasons for delays or dose modifications of all study treatment will be recorded on case report forms.

4.6 NON-TRIAL TREATMENT

4.6.1 MEDICATIONS PERMITTED/NOT PERMITTED

No other therapies for other prostate cancer (e.g. bilateral orchidectomy, oestrogens, cytotoxic chemotherapy) are acceptable prior to disease progression. 5-alpha reductase inhibitors, soya, selenium and vitamin E are acceptable non-trial therapies.

4.6.2 DATA ON CONCOMITANT MEDICATION

Concomitant medication relevant to serious adverse events will be recorded on Serious Adverse Event forms.

4.7 CO-ENROLMENT GUIDELINES

Ideally, patients should not be participating in any other clinical trial of prostate cancer treatment when they enter RADICALS. However, there are some planned trials that overlap and fit with RADICALS which patients may join if participation does not interfere with RADICALS or other trials. Similarly, once enrolled, patients should not enter any other trials that will interfere with the RADICALS assessments until the patient has had a treatment failure event reported (see **Section 9.2.2**). After this point, the patient may be entered into further studies.

The primary outcome measures of RADICALS are freedom from distant metastasis and disease-specific survival. Therefore, Follow-up to RADICALS must continue and must not be affected by co-enrolment to other studies. It is preferable that the participating group's trials unit should be notified in writing, with details of the trial: trial name, sponsor, randomisation arms, study endpoints and a declaration that RADICALS Follow-up will not be impeded, before a patient is co-enrolled or after randomisation if the patient is already co-enrolled.

5 ASSESSMENTS AND FOLLOW-UP

5.1 CASE REPORT FORM TIMINGS

Table 5 presents a summary of the timing of the required trial case report forms to be completed by the centre for participating patients.

Table 5: Summary of timing of case report forms (CRFs)

TRIAL CASE REPORT FORMS	TIMING FROM RANDOMISATION
Baseline Information form (CRF 1a)	Pre-randomisation
Patient History Form (CRF1b)	Pre- or Post-randomisation
Co-morbidity form (CRF 2)	Within two weeks prior to randomisation
PSA History Log	Pre-randomisation
Randomisation forms<br>(CRF 3 = RADICALS-RT and RADICALS-HD)<br>(CRF 4 = RADICALS-HD alone)	At randomisation
Radiotherapy forms (CRF 5)	After administration of radiotherapy
Follow-up forms*(CRF 6)	Month 4, 8, 12, 16, 20, 24, 30, 36, 42, 48, 54, 60, then annually until year 15
Patient Reported Outcome forms**	Pre-randomisation, 1, 5 and 10 years
Disease Event form (CRF 7)	As needed
Serious adverse event form (CRF 8)	As needed
Death Report form (CRF 9)	As needed

*Timed from most recent randomisation
**Patient reported outcomes only reported by patients in RADICALS-RT

5.2 PROCEDURES FOR ASSESSING EFFICACY

5.2.1 PSA MEASUREMENTS

PSA will be tested regularly at each follow-up visit and more often if clinically indicated. The assay used must have a sensitivity of 0.1ng/ml or lower.

5.2.2 EFFICACY PARAMETERS

The primary outcome measure is for RADICALS-RT is freedom from distant metastasis. This will be defined as any distant metastasis or death from prostate cancer. Bone scans are not mandated at set times but should be performed as clinically indicated. The primary outcome measure for RADICALS-HD is metastasis free survival, defined as any distant metastasis or death from any cause.

All men should be followed-up for the duration of the trial. With regards to ascertaining causes of death, particular attention will be paid to men who are reported as having died from prostate cancer without previously reporting progression or recurrence, and men who are reported as having died from non-prostate cancer causes after developing hormone refractory disease. There will be a review of causes of death performed independent from allocated trial arm.

5.3 PROCEDURES FOR ASSESSING SAFETY

There are no tests in addition to standard practice to assess patient safety. Patients will be seen every 4 months for 2 years, every 6 months from 2 to 5 years, then annually thereafter. Secondary malignancies, toxicities and SAEs will be recorded on CRFs and/or SAE forms which will be monitored by the Independent Data Monitoring Committee (IDMC).

5.4 OTHER ASSESSMENTS

5.4.1 PATIENT REPORTED OUTCOMES

Quality of life will be assessed using self-administered questionnaires in the subgroup of patients entered in RADICALS-RT. These questionnaires have approximately 50 questions and are collected pre-randomisation and at 1, 5 and 10 years after randomisation. Further details are given in **Section 13.1**.

5.4.2 HEALTH ECONOMICS

Data for the health economics substudy will be collected on both CRFs and patient administered questionnaires (EQ-5D). The EQ-5D questionnaire will be completed by the patients together with the patient reported outcome forms at baseline and at 1, 5 and 10 years after randomisation. Further details are given in **Section 13.2**.

5.5 EARLY STOPPING OF FOLLOW-UP

If a patient chooses to discontinue their trial treatment, they should still be followed up providing they are willing; if they do not wish to remain on trial follow-up, however, their decision must be respected and the patient will be withdrawn from the trial completely. The CTU should be informed of this in writing using the appropriate documentation. Patients withdrawing from follow up have a negative impact on a trial's data.

If the medical data collected during the patient's participation in the trial are kept for research and analysis purposes, they can be anonymised if necessary. Consent for future use of stored samples already collected can be refused when leaving the trial early (but this should be discouraged and should follow a discussion).

Patients may change their minds about stopping trial follow-up at any time and re-consent to participation in the trial.

Patients who stop trial follow-up early will not be replaced.

Patients will be followed up in the long-term through usual mechanisms, which may include flagging through national registries.

5.6 LOSS TO FOLLOW-UP

Every effort should be made to follow-up all patients who have been randomised. Patients should, if possible, remain under the care of an oncologist or urologist for the duration of the trial. If care of a patient is returned to the primary lead physician, it is the responsibility of the trial investigator who obtained the patient's consent to participate in the trial to ensure that the data collection forms are completed. For participants who stop follow up early, data collected up to the time of withdrawal

from follow up will be kept and included in the analysis unless the participant explicitly withdraws consent for this.

If follow-up visits are no longer possible according to the trial schedule, patients can be followed up by telephone or using information from other healthcare services, e.g. GP practice. These follow-ups should adhere to the timelines specified in the patient's schedule. If information reported on the follow-up CRF is not from a follow-up visit to a RADICALS investigator site please indicate this by completing question 1a on the Follow Up CRF.

Where it applies, the consent of patients should be obtained for their names to be flagged for survival information through national registries, for example NHS Information Centre/Office of National Statistics (ONS) in England/Wales and General Register Office in Scotland, Hospital Episode Statistics (HES). If the clinician moves, appropriate arrangements should be made to arrange for trial follow-up to continue at the centre.

5.7 PATIENT TRANSFERS

For patients moving from the area, every effort should be made for the patient to be followed-up at another participating trial centre and for this trial centre to take over responsibility for the patient. To document the transfer process the main contact person at both the current and receiving hospitals should complete and sign the Patient Transfer Confirmation form. A fully completed form must be returned to the CTU prior to the patient transfer and ideally any data queries for the patient should be completed prior to transfer.

On receipt of the completed transfer form a member of the RADICALS team will confirm the database has been updated and request confirmation of the name of the patient's new Clinician. Photocopies of the following documents may then be sent to the new hospital to complete the transfer and copies must be also retained at the original site for monitoring purposes:

- Consent form
- Completed CRFs
- Any documentation relating to the patient's participation in RADICALS (patient names must be removed from any documentation).

5.7.1 MORTALITY DATA FROM ELECTRONIC HEALTH RECORDS

Death registration data for England and Wales became available to the trial in January 2018, to be updated quarterly thereafter. These will be provided through ONS or an appropriate alternative. This enables regular checking for completeness of the trial database, with specific data chases to sites in the event of unreported deaths becoming known. The data also enables more precise survival estimation, since patients without recent trial follow-up may be assumed to be alive approximately one month before the data extract if death has not been registered. Death registration also enables checking for unreported distant metastases, in the event of a prostate cancer death being registered with ONS but no distant metastases previously recorded in the trial database. Data from from other national registers may be used in the future if it becomes available to the trial team.

5.8 END OF TRIAL

All patients will be actively followed according to the trial schedule until end of funding unless the TMG considers appropriate to transition before then to a long-term follow-up stage. During the long-term follow-up stage participants will not be required to make any trial specific visits to the clinic but will be followed-up via retrospective data collection from the sites and national registries for which

consent has been requested as part of the original consent process. During this follow-up period participants will have completed the interventional phase of the study and will not be on any trial mandated regimen so real time SUSAR and SAE reporting will cease but investigators will be asked to report events via national reporting systems such as the MHRA Yellow card system in the UK.

6 STATISTICAL CONSIDERATIONS

6.1 METHOD OF RANDOMISATION

Randomisation will be performed centrally at the MRC Clinical Trials Unit at UCL using a computer-implemented algorithm. The method of randomisation will be minimisation over a number of clinically important stratification factors with an allocation probability of 80%. Each comparison will have an independent randomisation programme.

6.2 OUTCOME MEASURES

RADICALS considers a number of primary and secondary outcome measures; all outcome measures are relevant to both trial randomisations unless otherwise stated. All outcome measures will be timed from the relevant randomisation.

6.2.1 PRIMARY OUTCOME MEASURE - RADICALS-RT

RADICALS-RT was originally designed to detect an absolute improvement of 5% in disease-specific survival at 10-years from 90% to 95% with 80% power. Since the trial was designed in 2006, further information on disease-specific survival in similar patient cohorts has become available from prospective RCTs, including EORTC 22911 (8) and SWOG 8794 (10). These provide useful estimates as they are both clinical trials and recruited partly during the PSA era; such information was not previously available. The results of these trials show that death from causes other than prostate cancer is a major competing risk, with around only one in four deaths being attributed to prostate cancer and a 10-year disease-specific-survival (DSS) of around 94%; this patient cohort is performing much better than had been anticipated originally. With further treatments (e.g. docetaxel) being available and standard of care at the time of relapse and the development of castrate-refractory disease, the time from the development of distant metastasis until death from prostate cancer has lengthened. An absolute improvement of 5% is not a reasonable assumption if these estimates hold true.

However, the role of adjuvant RT remains controversial and a clinical trial is required to resolve this issue. RADICALS-RT will do this. Therefore, focus has turned towards distant metastases, which is an earlier but clinically important and objective outcome measure. The primary outcome measure for RADICALS-RT is now **freedom-from-distant-metastasis**.

Disease-specific survival, therefore, becomes an important secondary outcome measure. At the main analysis of distant metastases, the trial would expect around 41 deaths from prostate cancer and would have 59% power to detect a halving of the risk of prostate cancer death from 6% at 10-years to 3%, ie DSS improves from 94% to 97%. With further follow-up, RADICALS-RT could attain 80% power for DSS after additional 6 years of follow-up (total duration of the trial would be 18.5 years). However, the question of DSS would be addressed sooner through combined analysis with two parallel trials: RAVES and GETUG-17 (see Section 9.6).

6.2.2 PRIMARY OUTCOME MEASURE – RADICALS-HD

After 2011, when the primary outcome in RADICALS-RT was amended to freedom-from-distant-metastasis, the primary outcome measure in RADICALS-HD was unchanged and remained disease-specific survival (DSS). Causes of death in patients diagnosed with prostate cancer can be difficult to confirm. A reported death from prostate cancer would be expected to be preceded by a report of hormone refractory metastatic prostate cancer. The clinician's discretion should be used to decide if death during treatment is related to prostate cancer. All UK patients will be flagged with the NHS

Central Register (NHSCR) or equivalent for mortality data to support the data collected on the case report forms (CRFs).

Measures such as central flagging ensure that deaths are captured on the database in an accurate and timely manner. However, the number of deaths from prostate cancer in RADICALS-HD patients continued to be very low and it became apparent that neither the 0 vs 6 month or 6 vs 24 month comparisons would accrue sufficient events for analysis within a realistic timeframe. Deaths from other causes also presented a problem for analysis as a competing risk, even though numbers of 'all cause' deaths were also lower than expected. It was therefore decided that the primary outcome of RADICALS-HD would now be metastases-free survival (**MFS).**.

6.2.3 SECONDARY OUTCOME MEASURES

- **Disease-specific-survival** – (RADICALS-RT only)
- **Freedom from treatment failure**: PSA progression when on androgen deprivation
- **Clinical progression-free survival**: Clinical progression of prostate cancer or initiation of non-protocol hormone therapy or death from prostate cancer.
- **Overall survival**: Death from any cause
- **Non-protocol hormone therapy**: Initiation of hormone therapy other than that randomised
- **Treatment toxicity**: Incidence of severe toxicity or serious adverse events. Radiotherapy treatment planning data is required to understand the cause of treatment toxixity.
- **Patient reported outcomes**: See Section 13.1 for details
- **Freedom from biochemical progression:** Where a biochemical progression event is definied as a PSA level of ≥0.4ng/ml following radiotherapy or a PSA level of > 2.0ng/ml regardless of prior radiotherapy.

6.2.4 EARLY REPORTING OF BIOCHEMICAL OUTCOMES

In early 2018, based on the number of primary outcome events in the control arm of the RADICALS-RT comparison, the target number of events was not forecast to occur until 2025. This is several years later than originally expected due to the low event rate. Two other randomised controlled trials (RCTs), RAVES and GETUG 17, also address the question of deferred RT and are planning to report in 2018/2019. The ARTISTIC project is a pre-planned individual patient data meta-analysis, combining RAVES, GETUG-17 and RADICALS-RT. The RADICALS TMG therefore agreed, in February 2018, to publish the Freedom from biochemical progression results of RADICAL-RT to coincide with the other two trials, and enable a timely meta-analysis.

6.3 SAMPLE SIZE

6.3.1 BASIC ASSUMPTIONS

The original sample size calculations were performed using the -art- package in Stata 9(31). The sample size re-calculation was performed in Stata 11.1. using version 1.0.8 (date 24mar2010) of –art-, including –artsurv- for the main calculation and –artpep- for variations. In terms of accrual and follow-up, we assume 5½ to 6½ years of recruitment, attaining a constant rate of accrual by 3 years after initiation of the trial in RADICALS-RT; a steady rate of accrual may be reached slightly later in time in RADICALS-HD as the rate may not peak until patients allocated deferred radiotherapy in RADICALS-RT start to experience biochemical failure. After recruitment, we assume a further 7 years of Follow-up. Clinically, we assume that, in RADICALS-RT, we should only be interested in treatment options with an absolute increase in 10-year freedom-from–distant-metastasis of 5%. In RADICALS-HD, we should only be interested in treatment options with an absolute increase in 10-year metastase-free survival of 6%, at least.

The sample sizes have been calculated separately for the two randomisations because of potential variation in the underlying assumptions, an uncertain proportion of patients joining RADICALS-RT

and RADICALS-HD and some uncertainty about accrual rates. A number of scenarios relating to trial recruitment and assumptions have been calculated and are reported elsewhere but are available upon request. Selected scenarios are reported here.

6.3.2 RADICALS-RT SAMPLE SIZE

In the patients suitable for this randomisation, the control arm is assumed to be deferred radiotherapy i.e. radiotherapy at PSA relapse.

When the trial was originally designed, there was uncertainty about the likely event rate but it was anticipated that a modest absolute effect in the order of 5% would be required to convince clinicians to adopt adjuvant radiotherapy for all patients. The original sample size calculations anticipated that around 2,600 patients would need to be recruited over 5½ years and followed-up for a further 7 years in order to obtain 80% power to detect an improvement from 70% to 75% or 90% power to detect an improvement from 80% to 85%.

From SWOG 8794 and EORTC 22911, the proportion of patients free of distant metastases at 10 years is estimated to be 90%. We would look to test whether adjuvant RT can improve this to 95% (hazard ratio 0.487), which is seen as the minimum clinically significant improvement required to routinely introduce adjuvant RT to this patient population; this mirrors the size effect observed in SWOG 8794. This is tested using the superiority design. With 80% power, a two-sided 5% significance level, accrual lasting 5½ years (reaching peak accrual rates after 3 years) and a further 7 years of follow-up, we would need to recruit 1,063 patients in order to observe 66 distant metastases events. This sample size assumes that 30% of patients are lost to follow-up and around 30 patients per month randomised from 30 months onward. Therefore, the target sample size will be reduced from 2,600 to around 1,063 patients. This assumes that 30% of patients are lost to follow-up between 5 and 10 years.

If the peak accrual is lower, at around 25 patients per month, accrual would be extended by 1 year, to around 6½ years and around 1,160 patients would be randomised; this should address the question with the same power and in the same overall timescale.

6.3.3 RADICALS-HD SAMPLE SIZE

Patients would be suitable for this randomisation if they are planned for post-operative radiotherapy regardless of whether this is the early or deferred setting.

There is some uncertainty in the baseline disease-specific survival (DSS) rate for patients receiving early RT and for patients receiving deferred RT. It is assumed that patients receiving early radiotherapy do at least as well as patients receiving deferred radiotherapy, timed from randomisation to RADICALS-HD. There is also uncertainty over the proportion of early and deferred patients that would join the trial; it is assumed that at least as many patients in the deferred setting will be randomised, if not two to three times more.

Since the trial was designed in 2006, further information on the baseline estimates of disease specific survival have become available from similar patient cohorts, including data from the RTOG 9601, SWOG 8794 and EORTC 22911 trials. These provide useful estimates as they are clinical trials and recruited partly during the PSA era; such information was not previously available when RADICALS-HD was designed.

Sites and patients are encouraged to join the three-way randomisation of no-HT vs STHT vs LTHT in RADICALS-HD as this is the most efficient for the trial. However, it has become apparent that the three-way randomisation is less well supported than either of the two separate potential two-way randomisations: no-HT vs STHT and STHT vs LTHT. These are each clinically important questions and

the trial will address both. It will not be possible to address one of the originally envisaged comparisons: no-HT vs LTHT with any reasonable degree of power, although the comparison will be performed. Therefore, there are two main comparisons in RADICALS-HD:

1. RT-only (no-HT) vs RT + short-term hormone therapy (STHT)
2. RT+ short-term HT (STHT) vs RT + long-term HT (LTHT)

It is assumed that patients who enter RT+STHT vs RT+LTHT comparison have a slightly higher risk of a disease event than patients who enter RT-only vs RT+STHT comparison because the clinician assumes that some HT is required; therefore, their 10 year DSS rate is estimated as being lower.

6.3.3.A COMPARISON: NO-HT VS RT+STHT

Original power calculations:
It was estimated that DSS would be 85% at 10 years in patients on the no-HT arm. This superiority trial is testing whether addition of STHT to RT can improve this to 91% (hazard ratio HR=0.58). A total of 1263 patients (128 events) in a comparison of no-HT vs STHT would allow for 80% power to detect an increase of 6% in 10-year DSS with a 3% significance level (accounting for the multiple use patients who join the three-way randomisation). This assumes that 30% of patients are lost to follow-up. Peak accrual would be around 31 patients per month in the comparison of no-HT vs RT+STHT.

If peak accrual is lower, at around 25 patients per month, accrual would be extended by 1 year to around 6.5 years and around 1368 patients would be randomised; this should address the question in the same timescale with the same power.

Updated power calculations:
The primary outcome measure of RADICALS-HD was amended from DSS to MFS after peer review in 2019 and without any reference to accumulating, comparative data. At this point, recruitment was already completed with 1480 patients in the 0 vs 6 months comparison. It is estimated that MFS will be approximately 80% at 10 years in patients allocated to the no-HT group. The 0 vs 6 months comparison would have over 80% power, with a two-sided 5% alpha, to detect an absolute increase in MFS of 6%, from 80% in the no-HT group to 86% (HR=0.67) in the short-HT group. Approximately 200 MFS events are anticipated for this analysis.

6.3.3.B COMPARISON: RT+STHT VS RT+LTHT

Original power calculations:
It was estimated that DSS would be 87% at 10 years in patients on the STHT arm. This is lower than the estimated 91% 10-yr DSS in the research arm (STHT) in the previous comparison if STHT is more effective than no-HT, as we anticipate that higher risk patients will enter the STHT vs LTHT comparison. This superiority trial is testing whether the LTHT can improve disease-specific survival to 93% at 10 years (hazard ratio HR=0.52). A total of 1077 patients (91 events) in a comparison of STHT vs LTHT would allow for 80% power to detect an increase in 10-year DSS of 6% with a 3% significance level (accounting for the multiple use patients who join the three-way randomisation). This assumes that 30% of patients are lost to follow-up.

This could be achieved in 51/2 years with 26 patients randomised each month from 30 months onwards. Patients would be followed-up for 7 years after the end of recruitment. The numbers above allow for a certain percentage of patients to be lost to follow-up.

If the peak accrual is lower, at around 20 patients per month, accrual would be extended by 1 year to around 6.5 years and around 1129 patients would be randomised; this should address the question in the same timescale with the same power.

Updated power calculations:
The primary outcome measure of RADICALS-HD was amended from DSS to MFS after peer review in 2019 and without any reference to accumulating, comparative data. At this point, recruitment was already completed with 1524 patients in the 6 vs 24 months comparison. It is estimated that MFS will be approximately 75% at 10 years in patients in patients allocated to the 6-month group of the 6 vs 24 months comparison; it is expected that patients in this comparison will be at higher risk of an event than those in the 0 vs 6 months comparison. The 6 vs 24 months comparison should have over 80% power, with a two-sided 5% alpha, to detect an absolute increase in MFS of 6%, from 75% in the short-HT group to 81% in the long-HT group (HR=0.72). Approximately 300 MFS events are anticipated for this analysis.

6.3.4 OVERALL SAMPLE SIZE

The overall sample size will depend on how many patients are recruited to both RADICALS-RT and RADICALS-HD, and how many patients join the three-arm Hormone Duration Randomisation. It is anticipated that, of patients who have undergone radical prostatectomy, 10% will have a definite indication for non-randomised early radiotherapy and 50% will have a definite indication for following a non-randomised policy of deferred radiotherapy. The value of early radiotherapy will be uncertain in the remaining 40% who, if they meet the eligibility criteria, should be randomised in RADICALS-RT. No formal overall sample size is estimated, but around 2500 patients will be recruited.

Given the number of radical prostatectomies performed each year in the participating countries, these are feasible target sample sizes.

6.3.5 PILOT PHASE

RADICALS incorporates an 18-month feasibility stage during which randomisation rates, and the trial as a whole, will be carefully assessed. Continuation of the trial beyond the feasibility stage was conditional on satisfactory patient accrual. The trial’s progress has been repeatedly reviewed by the TMG, TSC and IDMC as well as the funding body. The decision to review and update the sample size calculations has been taken with full discussion and support, particularly by given the efforts to encourage the accrual. Accrual rates will be monitored throughout the trial.

6.4 INTERIM MONITORING AND ANALYSES

Formal interim analyses of the accumulating data will be performed at regular intervals (at least, annually) for review by an Independent Data Monitoring Committee (IDMC, see **Section 15.3**). These analyses will be performed by statisticians at the MRC CTU at UCL. The IDMC will be asked to give advice on whether the accumulated data from the trial, together with results from other relevant trials, justifies continuing recruitment of further patients or further Follow-up. A decision to discontinue recruitment, in all patients or in selected subgroups, would be made only if the result is likely to convince a broad range of clinicians including participants in the trial and the general clinical community. If a decision is made to continue, the IDMC will advise on the frequency of future reviews of the data on the basis of accrual and event rates. The IDMC will make recommendations to the Trial Steering Committee (TSC, see **Section 15.2**) as to the continuation of the trial.

The trial oversight committees will be asked to continue to monitor and comment on any deviation of the accruing data from the underlying assumptions e.g. higher or lower rates of death from prostate cancer than expected or type of patient randomised.

6.5 STATISTICAL ANALYSIS PLAN

The analyses to be performed for RADICALS will be presented in detail in a separate Statistical Analysis Plan. In short, the main analyses will be performed for patients in RADICALS-RT and separately for patients in RADICALS-HD. The main outcome measures will be compared using the standard time-to-event methods of Kaplan-Meier with formal comparisons using log-rank tests and graphically represented with survival plots. Analyses in the hormone duration comparisons will be stratified by the timing of post-operative radiotherapy (early RT vs deferred RT).

6.6 INDIVIDUAL PATIENT DATA META-ANALYSIS

6.6.1 RADICALS-RT

In collaboration with the two parallel trials to RADICALS-RT, RAVES and GETUG-17, and the Meta-analysis Group of the MRC-CTU at UCL, the ARTISTIC collaborative group has been formed in order to prospectively plan a meta-analysis. Pooling summary results and relevant statistics for event free survival from all three trials will facilitate a meta-analysis of almost 2000 patients in total. The resulting increase in power and precision that the meta-analysis provides will enable a timely and definitive assessment of the treatment effects of adjuvant RT in all patients as well as potentially enabling an assessment of treatment effects within subgroups of patients, defined for example by risk groups or by age at randomisation. In due course, as trial outcome data matures, the ARTISTIC collaboration will undertake a full meta-analysis based on individual participant data to assess all outcome measures (see section 1.1.4.A).

6.6.2 RADICALS-HD

Other international groups are conducting overlapping clinical trials therefore, we plan to also undertake a meta-analysis using IPD including RADICALS-HD, GETUG-16, EORTC 22043/30041, RTOG 05-34 (SPPORT) and RTOG 96-01 and any other relevant trial identified in a full systematic review. Together these trials will provide increased power for analysis of both disease-specific survival and overall survival. Formal agreements will be developed with all trial groups.

7 QUALITY ASSURANCE AND CONTROL

7.1 COMPLIANCE

RADICALS will be conducted according to the protocol, relevant Standard Operating Procedures (SOPs), GCP and relevant national regulatory requirements.

7.2 RADIOTHERAPY QUALITY ASSURANCE

The RADICALS Radiotherapy Trials Quality Assurance (RTTQA) Group, consisting of radiation oncologists and radiotherapy physicists, will give information and guidance regarding implementation of the protocol, monitor compliance with the protocol, and provide feedback on the RTQA accreditation (where necessary).

RTQA accreditation is required by all centres (see **Appendix B**). However, centres that have been RTQA accredited for another multi-centre prostate radiotherapy trial in the UK (e.g. MRC RT01 or CHHIP) will be automatically granted RADICALS-RTQA accreditation.

The RADICALS website will include sample cases to illustrate the Clinical Target Volumes described in **Section 6.1.3.A**.

Data will be collected by the NCRI Radiotherapy Trials QA (RTTQA) Group for patients treated in the RADICALS trial. This includes: CT images, contours, plan and plan dose cubes along with DVHs. Data must be appropriately anonymised.

7.3 MONITORING AND AUDIT

A risk assessment has been carried out and an appropriate monitoring plan has been developed. Monitoring will be a combination of central monitoring (e.g. database checks) and the IDMC review as described in **Section 15.3**. There will be limited on-site monitoring: all participating investigators and groups must agree to direct access to all trial related sites, source data documents and reports for the purpose of monitoring by the sponsor and audit and inspection by domestic and foreign regulatory authorities.

7.4 SOURCE DATA

The investigator/institution should maintain adequate and accurate source documents and trial records that include all pertinent observations on each of the site's trial subjects. Source data are contained in source documents and are defined by EU guidelines as all information in original records that are used for the reconstruction and evaluation of the clinical trial. Source documents are the first place where the source data is recorded. These can include hospital records, clinical and office charts, laboratory notes, X-rays, and pharmacy dispensing records.

Source data should be attributable, legible, contemporaneous, original, accurate, and complete. Changes to source data should be traceable, should not obscure the original entry, and should be explained if necessary (e.g., via an audit trail). Each data element should only have one source.

Data will be recorded on case report forms (CRF). The original should be sent to the appropriate participating group and a copy kept at the local centre. The type of data to be recorded is detailed in Section 7, the Assessments and Procedures section.

The following data should all be verifiable from source documents, which may include paper notes and electronic health records:

- signed consent and (where applicable) assent forms
- dates of visits including dates any trial specimens were taken and processed in the laboratory
- eligibility and baseline values
- adverse events of any grade that lead to treatment modification and adverse events judged definitely/probably/possibly related to IMP
- severe (grade 3/4) adverse events
- serious adverse events
- dates IMP (hormone therapy) was dispensed and administered

7.4.1 DATA HANDLING

The site will retain a copy of each CRF. All data recorded in each CRF, will be entered onto the RADICALS trial clinical database. A comprehensive validation check program will identify missing, illogical and/or inconsistent data. Trained data management personnel will review the resulting discrepancy report, correcting any data entry errors. If investigator input is required to clarify or correct any missing, ambiguous or inconsistent data, the data manager will generate a Data Clarification Form (DCF). The Data Manager will send this form to the investigator for completion. When the completed DCF is returned to data management, the data on the clinical database will be corrected accordingly.

7.5 PROTOCOL DEVIATIONS

If the site identifies a protocol deviation they should notify the PI within 24 hours. The PI and team should review the deviation and create a suggested corrective action plan. This corrective plan should be reported to the MRC CTU team, who will review it and the deviation. The site will keep a protocol deviation log and the completed corrective action plan in order to ensure, as far as possible, that the deviation does not reoccur.

8 SAFETY REPORTING

ICH GCP requires that both investigators and sponsors follow specific procedures when notifying and reporting adverse events/reactions in clinical trials. These procedures are described in this section of the protocol. **Section 11.1** lists definitions, **Section 11.3** gives details of the institution/investigator responsibilities and **Section 11.4** provides information on MRC CTU at UCL responsibilities.

8.1 DEFINITIONS

The definitions of the EU Directive 2001/20/EC Article 2 based on ICH GCP apply in this trial protocol. These definitions are given in **Table 6**. These definitions apply to RADICALS investigators in the UK and Canada.

Table 6: Terms and definitions for adverse events

TERM	DEFINITION
Adverse Event (AE)	Any untoward medical occurrence in a patient or clinical trial subject to whom a medicinal product has been administered including occurrences which are not necessarily caused by or related to that product.
Adverse Reaction (AR)	Any untoward and unintended response to an investigational medicinal product related to any dose administered.
Unexpected Adverse Reaction (UAR)	An adverse reaction, the nature or severity of which is not consistent with the information about the medicinal product in question set out in the summary of product characteristics (or Investigator brochure) for that product.
Serious Adverse Event (SAE) or Serious Adverse Reaction (SAR) or Suspected Unexpected Serious Adverse Reaction (SUSAR)	Respectively any adverse event, adverse reaction or unexpected adverse reaction that:<br>• results in death<br>• is life-threatening*<br>• requires hospitalisation or prolongation of existing hospitalisation**<br>• results in persistent or significant disability or incapacity<br>• consists of a congenital anomaly or birth defect<br>• is another important medical condition***

8.2 CLARIFICATIONS

*Life-threatening, in the definition of 'serious', refers to an event in which the patient was at risk of death at the time of the event; it does not refer to an event which hypothetically might have caused death if it were more severe.

**Hospitalisation is defined as an inpatient admission, regardless of length of stay, even if the hospitalisation is a precautionary measure for continued observation. Hospitalisations for a pre-existing condition (including elective procedures that have not worsened) do not constitute an SAE.

***Medical judgement should be exercised in deciding whether an AE/AR is serious in other situations. Important AE/ARs that are not immediately life-threatening or do not result in death or

hospitalisation but may jeopardise the subject or may require intervention to prevent one of the other outcomes listed in the definition above, should also be considered serious.

Please note that SAEs should also be reported as routine toxicities where that toxicity is also collected on one of the routine assessment forms.

8.2.1 Medicinal Products

An investigational medicinal product is defined as the tested investigational medicinal product and the comparators used in the study. (EU guidance ENTR/CT 3, April 2006 revision).

Adverse reactions include any untoward or unintended response to drugs. Reactions to an IMP or comparator should be reported appropriately.

8.2.2 Adverse Events

Adverse Events include:

- An exacerbation of a pre-existing illness
- An increase in frequency or intensity of a pre-existing episodic event or condition
- A condition (even though it may have been present prior to the start of the trial) detected after trial drug administration
- Continuous persistent disease or a symptom present at baseline that worsens following administration of the study treatment

8.3 DISEASE RELATED EVENTS AND ELECTIVE HOSPITALISATION

SAEs related to disease progression, or death as a result of disease progression, are exempt from expedited reporting and should be reported on the Follow Up Form(CRF 9), Disease Event Form (CRF 7) and/or Death Report Form (CRF 9) instead of the SAE Form.

The following situations regarding elective hospitalisation that fulfil the definition of an SAE are also excluded from expedited reporting and should be reported on the Follow-up Form instead of the SAE Form:

- Elective hospitalisation to simplify treatment or procedures
- Elective hospitalisation for pre-existing conditions that, in the investigator's opinion, have not been exacerbated by trial treatment

There are no other treatment-related toxicities that result in hospitalisation for symptom control which are excluded from expedited reporting. Life-threatening or fatal events should still be reported on the SAE form.

8.3.1 Institution Responsibilities

All non-serious AEs/ARs, whether expected or not, should be recorded in the patient's medical notes. Specific toxicities should be reported for the trial in the toxicity (symptoms) section of the Follow-up form (CRF 6) and sent to the MRC CTU at UCL within one month of the form being due. The specific toxicities reported for the trial are diarrhoea, proctitis, cystitis, haematuria, urethral strictue & rectal haemorrhage. SAEs/SARs should be notified to the MRC CTU at UCL as described below.

The severity (i.e. intensity) of all AEs/ARs (serious and non-serious) in this trial should be should be graded using the NCI CTCAE v3.0. The full list is available at https://ctep.cancer.gov/protocolDevelopment/electronic_applications/docs/ctcaev3.pdf.

A flowchart is given at the end of this section to help explain the notification procedures. Any questions concerning this process should be directed to the MRC CTU at UCL in the first instance.

8.3.2 Investigator Responsibilities

All AEs, should be recorded in the patient's medical notes and reported in the toxicity (symptoms) section of the Follow-up Form and sent to the CTU within the agreed timescale (see **Table 5, Section 7.1**). SAEs should be notified to the CTU within 24 hours of the investigator becoming aware of the event.

8.3.3 Investigator Assessment

8.3.3.A Seriousness

When an AE/AR occurs, the investigator responsible for the care of the patient must first assess whether the event is serious using the definition given **in Table 6**. If the event is serious and not exempt from expedited reporting (see Safety Reporting Flowchart), then an SAE form must be completed and the trials unit notified within 24 hours of becoming aware of the event.

8.3.3.B Causality

The Investigator must assess the causality of all serious events/reactions in relation to the trial therapy using the definitions in **Table 7**. There are 5 categories: unrelated, unlikely, possible, probable and definitely related. If the causality assessment is unrelated or unlikely to be related the event is classified as a SAE. If the causality is assessed as either possible, probable or definitely related then the event is classified as a SAR.

Table 7: Assigning Type of SAE Through Causality

Relationship	Description	SAE Type
Definitely	There is clear evidence to suggest a causal relationship and other possible contributing factors can be ruled out.	SAR
Probable	There is evidence to suggest a causal relationship and the influence of other factors is unlikely.	SAR
Possible	There is some evidence to suggest a causal relationship (for example, because the event occurs within a reasonable time after administration of the trial medication). However, the influence of other factors may have contributed to the event (for example, the patient's clinical condition, other concomitant treatments).	SAR
Unlikely	There is little evidence to suggest that there is a causal relationship (for example, the event did not occur within a reasonable time after administration of the trial medication). There is another reasonable explanation for the event (for example, the patient's clinical condition, other concomitant treatment).	Unrelated SAE
Unrelated	There is no evidence of any causal relationship	Unrelated SAE

If an SAE is considered to be related to trial treatment and drug is stopped or the dose modified, refer to **Section 10.3.3.E.**

8.3.3.C EXPECTEDNESS

If there is at least a possible involvement of the trial treatment (or comparator), the investigator may make an initial assessment of the expectedness of the event, however the Sponsor has the overall responsibility for determination of expectedness. In the case of hormone therapy, an unexpected adverse reaction is one not previously reported in the current Reference Safety Information (approved versions located on trial website - http://www.radicals-trial.org/radicals-members-area/) or one that is more frequent or more severe than previously reported. The definition of an unexpected adverse reaction (UAR) is given in **Table 7**.

The main expected short term side-effects which may occur during or after radiotherapy treatment include tiredness, skin irritation, pubic hair loss, urinary frequency, decreased urinary stream, haematuria, need for urinary catheter, diarrhoea and bowel urgency. Late effects of radiotherapy treatment include bowel urgency, frequency or bleeding, urinary frequency, urinary urgency, erectile dysfunction, infertility, and increased risk of bladder or bowel cancer.

8.3.3.D NOTIFICATION

The MRC CTU at UCL should be notified within 24 hours of the investigator becoming aware of an event that requires expedited reporting. Investigators should notify the MRC CTU at UCL of all SAEs occurring from the time of randomisation until 30 days after the last protocol treatment administration. SARs and SUSARs must be notified to the MRC CTU at UCL until the submission of the end of trial notification (i.e. no matter when they occur after randomisation). Any subsequent events that may be attributed to treatment should be reported to the MHRA using the yellow card system.

8.3.3.E NOTIFICATION PROCEDURE:

a. The SAE form must be completed by the Investigator (named on the Signature List and Delegation of Responsibilities Log, who is responsible for the patient's care; this will be either the Principal Investigator or another medically qualified person with delegated authority for SAE reporting)), with due care being paid to the grading, causality and expectedness of the event as outlined above. In the absence of the responsible investigator the form should be completed and signed by a member of the site trial team and faxed or emailed as appropriate. The responsible investigator should subsequently check the SAE form, make changes as appropriate, sign and then re-fax to the MRC CTU at UCL as soon as possible. The initial report shall be followed by detailed, written reports as appropriate.

b. The minimum criteria required for reporting an SAE are the trial number, name of investigator reporting, the event, and why it is considered serious.

c. Send the SAE form by fax or email to the MRC CTU at UCL within 24 hours of the investigator's knowledge of the event.
   Fax Number: see box, below

d. Follow-up: Patients must be followed-up until clinical recovery is complete and laboratory results have returned to normal or baseline, or until the event has stabilised. Follow-up should continue after completion of protocol treatment if necessary. Follow-up information should be noted on a further SAE form by ticking the box marked 'Follow-up' and faxing to the MRC CTU at UCL as information becomes available. Extra, annotated information and/or copies of test results may be provided separately. The patient must be identified by trial number, date of birth and initials only. The patient's name should not be used on any correspondence and should be deleted from any test results.

   Staff should follow their institution's procedure for local notification requirements.

8.4 CTU RESPONSIBILITIES

Medically qualified staff at the MRC CTU at UCL and/or the Chief Investigator (or a medically qualified delegate) will review all SAE reports received. The causality assessment given by the local Investigator at the hospital cannot be overruled and in the case of disagreement, both opinions will be provided in any subsequent reports.

The MRC CTU at UCL is undertaking the duties of trial sponsor and is responsible for the reporting of all SUSARs and other SARs to the regulatory authorities (MHRA and competent authorities of other European member states and any other countries in which the trial is taking place) and the research ethics committees as appropriate. Fatal and life-threatening SUSARs must be reported to the competent authorities within 7 days of the CTU becoming aware of the event; other SUSARs must be reported within 15 days.

The MRC CTU at UCL will also keep all investigators informed of any safety issues that arise during the course of the trial.

The CTU, as Sponsor, will submit Annual Safety Reports in the form of a Developmental Safety Update Report (DSUR) to Competent Authorities (Regulatory Authority and Ethics Committee).

SAE NOTIFICATION

WITHIN 24 HOURS OF BECOMING AWARE OF AN SAE, PLEASE FAX A COMPLETED SAE FORM TO THE MRC CLINICAL TRIALS UNIT AT UCL ON:

FAX: +44 (0)20 7670 4818

Safety Reporting Flowchart

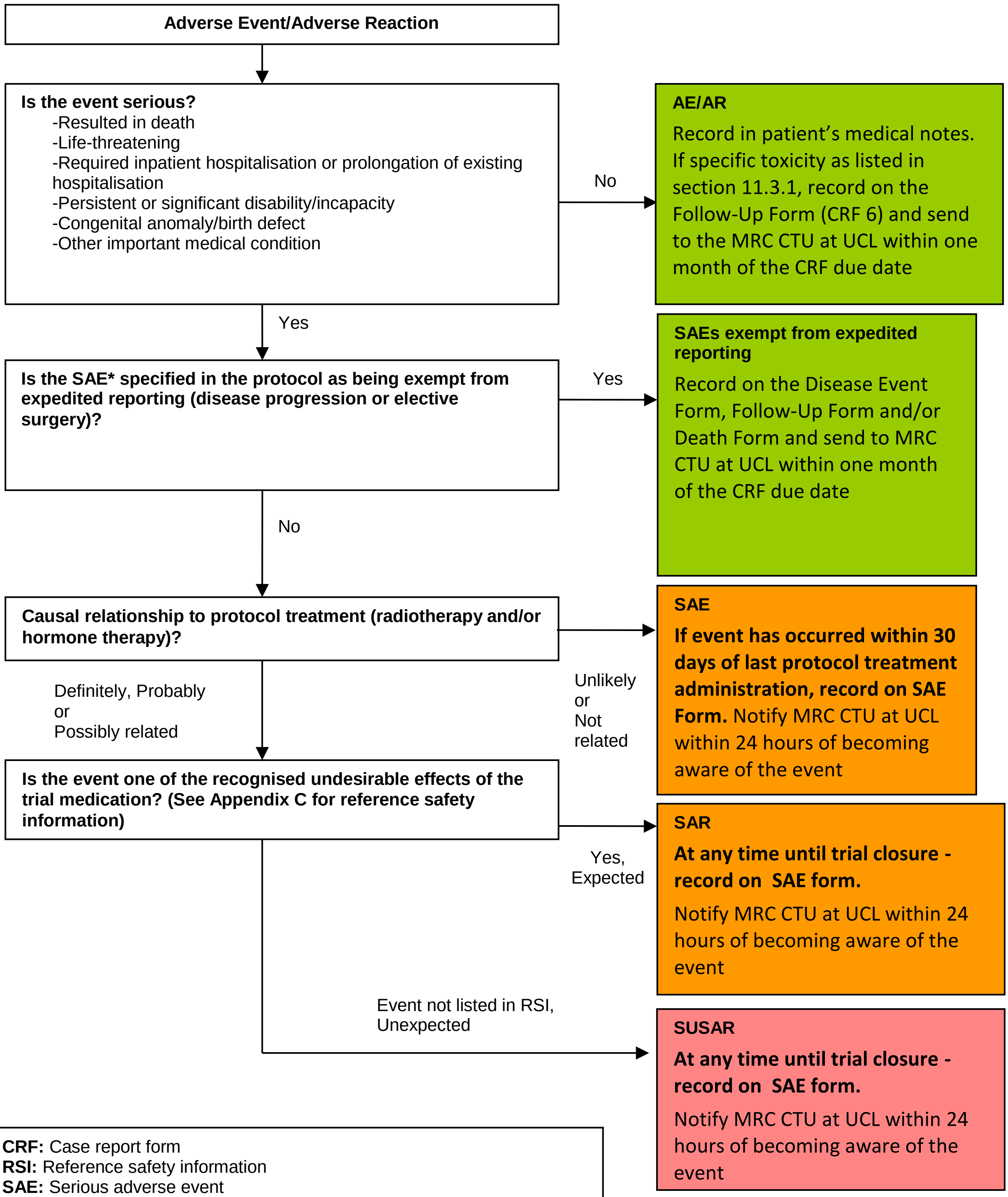

CRF: Case report form
RSI: Reference safety information
SAE: Serious adverse event
SAR: Serious adverse reaction
SmPC: Summary of product characteristics
SUSAR: Suspected unexpected serious adverse reaction

*All SAEs should be assessed for causality

9 ETHICAL CONSIDERATIONS

Participation in a randomised controlled trial means that the patient and clinician are not able to choose all aspects of patient treatment but do choose to be randomised. Patients will receive different treatments and toxicities are different by arm; this will all be explained to patients. All trial treatments and Follow-up schedules are routine practice across the UK. This trial is designed to fit with clinical practice; there are:

- No additional visits or assessments required by the trial
- No additional risks caused by participating in the trial and trial treatment.

The study will abide by the principles of the Declaration of Helsinki. Each patient's consent to participate in the trial should be obtained after a full explanation of the treatment options, including the conventional and generally accepted methods of treatment.

The right of the patient to refuse to participate in the trial without giving reasons must be respected. After the patient has entered the trial, the clinician must remain free to give alternative treatment to that specified in the protocol, at any stage, if he/she feels it to be in the best interest of the patient. However, the reason for doing so should be recorded and the patient will remain within the trial for the purpose of Follow-up and data analysis according to the treatment option to which they have been allocated. Similarly, the patient must remain free to withdraw at any time from the protocol treatment and trial Follow-up without giving reasons and without prejudicing his/her further treatment.

The investigator must ensure that patient's anonymity will be maintained and that their identities are protected from unauthorised parties. On CRFs patients will not be identified by their names, but by an identification code. The investigator should keep a patient enrolment log showing codes, names and addresses.

A statement of MRC policy on ethical considerations in clinical trials of cancer therapy, including the question of informed consent, is available from the MRC Head Office web site (https://mrc.ukri.org/research/policies-and-guidance-for-researchers/
).

10 ANCILLARY STUDIES

10.1 PATIENT-REPORTED OUTCOMES

There are limited patient-reported data (Quality of Life (QL) scores) on symptoms and morbidities associated with treatments after radical prostatectomy. No single questionnaire can adequately collect data in all of these areas. Therefore, patients will be asked to complete a number of short questionnaires.

Patient-reported outcome data will be collected from patients in the Radiotherapy Timing Randomisation in the UK and Canada, at least, via self-administered questionnaires. The questionnaires will assess general quality of life and health economics (SF-12, EQ-5D), urinary function (ICSmaleSF), bowel function (Vaizey) and sexual function (SHIM: IIEF-5).

The main objectives of this study are to determine the impact of:

- RT on general QL, sexual function, urinary function and bowel function
- Duration of hormone therapy on general quality of life, and sexual function

QL will be assessed prior to randomisation and at 1, 5 and 10 years. A separate quality of life protocol describes the study in more detail (see **Appendix A VI**).

10.2 HEALTH ECONOMICS

It is expected that clinical and quality of life issues will primarily drive the interpretation of trial results. However, data will be collected to allow potential health economic analyses. The trial will collect core data on resource use (treatments, in-patient and out-patient hospitalisations), and patients will regularly complete EQ-5D questionnaires which will generate preference-based measures of quality of life for possible calculation of quality-adjusted life-years. The trial will take around a decade to mature given the usually good prognosis of this patient group. A separate sub-protocol will be developed prior to any planned analyses.

10.3 TRANSLATIONAL RESEARCH

Optional translational studies will be planned and introduced early during the trial, subject to funding applications. The protocol will be amended appropriately to reflect any changes regarding translational studies.

11 APPROVALS AND INDEMNITY

11.1 ETHICS APPROVALS

The trial protocol has received the favourable opinion of a main Research Ethics Committee or Institutional Review Board (IRB) in the approved national participating countries. Local ethics approvals and other related documentation required are detailed in local **Appendix B**.

11.2 REGULATORY APPROVAL

This is a trial of Investigational Medicinal Products (IMPs) and therefore must be approved by the national competent authority. Details of national approvals are given in local **Appendix B**.

11.3 INDEMNITY

Each collaborating group has ensured that appropriate arrangements for indemnity to cover the liability of the investigator, including insurance where necessary, have been made according to their national guidelines. See guidelines in local **Appendix B**.

12 TRIAL COMMITTEES

12.1 TRIAL MANAGEMENT GROUP (TMG)

A Trial Management Group (TMG) has been formed comprising the Chief Investigator, other lead investigators (clinical and non-clinical), PPI contributors and members of the Data Centres. The TMG will be responsible for the day-to-day running and management of the trial and will meet at least 3 times a year by teleconference. Further details of TMG functioning are presented in the TMG charter.

12.2 TRIAL STEERING COMMITTEE (TSC)

The Trial Steering Committee (TSC) has membership from TMG plus independent members, including the chair. The role of the TSC is to provide overall supervision for the trial and provide advice through its independent Chairman. The ultimate decision for the continuation of the trial lies with the TSC. Further details of TSC functioning are presented in the TSC charter.

12.3 INDEPENDENT DATA MONITORING COMMITTEE (IDMC)

The Independent Data Monitoring Committee (IDMC) is the only group who sees the confidential, accumulating data to the trial. Reports to the IDMC will be produced by the MRC CTU at UCL statisticians. The IDMC will meet within 6 months of the trial opening with the frequency of meetings dictated by the IDMC. The IDMC will consider data in accordance with the analysis plan (see Section 9.5) and will be advisory to the TSC. The IDMC can recommend premature closure or reporting of the trial, or that recruitment to any research arm be discontinued.

Further details of IDMC functioning, and the procedures for interim analysis and monitoring are provided in the IDMC charter.

12.4 ENDPOINT REVIEW COMMITTEE (ERC)

The Endpoint Review Committee will be a small group, comprising at least one person blind to allocated treatment, will review the primary outcome measure (prostate cancer deaths). Details of the criteria and principles are part of the analysis plan which is in **Section 9.5**.

12.5 QUALITY OF LIFE SUBGROUP

The Quality of Life Subgroup issues guidance surrounding quality of life, including selection of the quality of life tools, and guidance on administration of the questionnaire.

12.6 RADIOTHERAPY QUALITY ASSURANCE SUBGROUP

The Radiotherapy Quality Assurance Subgroup developed the RT quality assurance (QA) plan and issued guidance on delivering RT in this trial.

12.7 PATHOLOGY QUALITY ASSURANCE SUBGROUP

The Pathology Quality Assurance Subgroup developed the pathology QA plan and issued guidance on reporting pathology for trial.

12.8 TRANSLATIONAL STUDIES GROUP

The Translational Studies Group aim to develop and implement appropriate and high quality bolt-on studies.

Figure 4: Diagram of relationships between trial committees

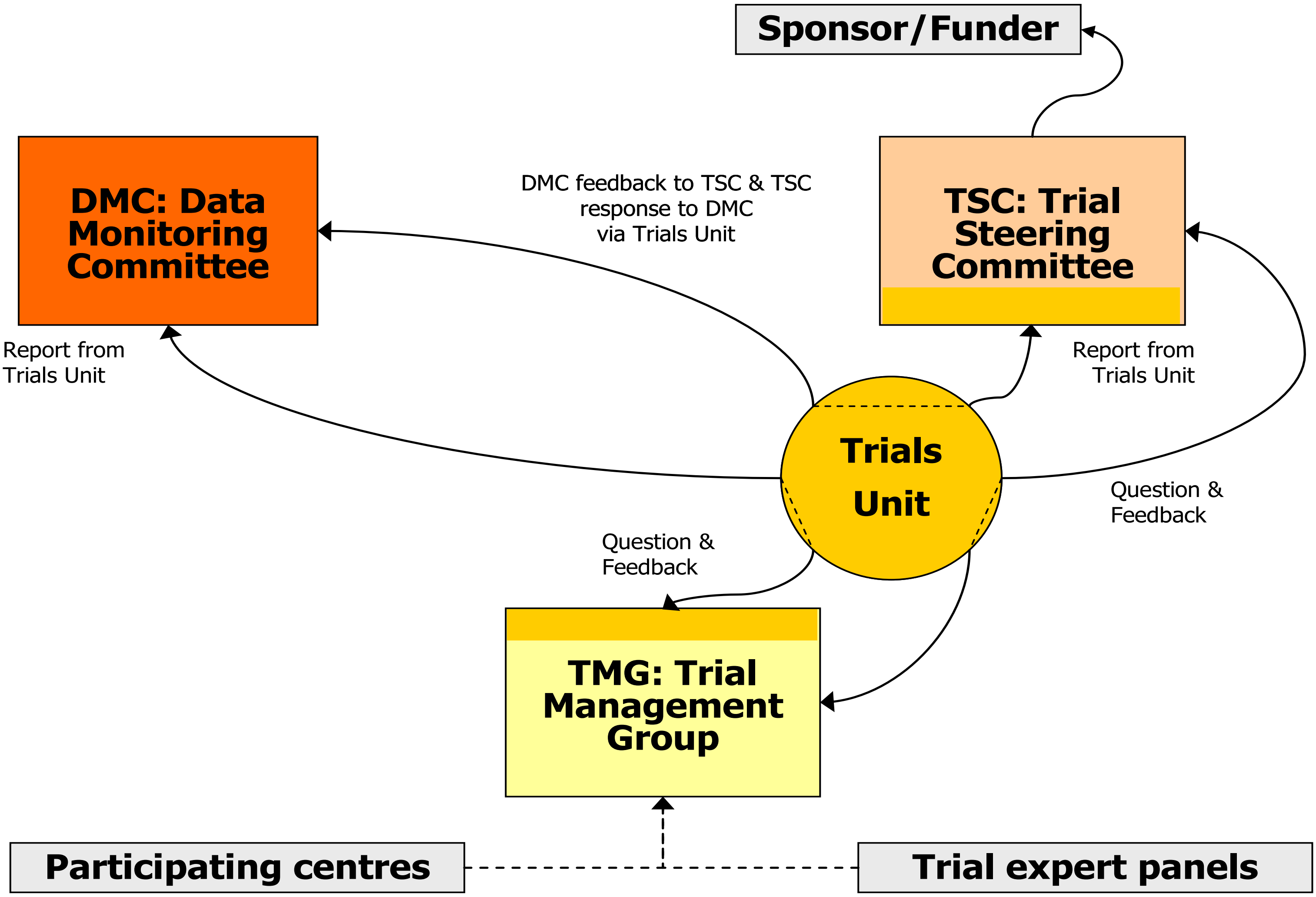

13 PUBLICATION AND DISSEMINATION OF RESULTS

The results from different centres and participating groups will be analysed together and published as soon as possible. Individual groups/clinicians must not publish data concerning their patients that are directly relevant to questions posed by the study until the Trial Management Group has published its report. The Trial Management Group will form the basis of the Writing Committee and will advise on the nature of all publications.

14 PROTOCOL AMENDMENTS

This is version 7.0 of the protocol.

14.1 PROTOCOL

14.1.1 AMENDMENTS MADE TO PROTOCOL VERSION 1.0 MARCH 2007

1. Page ii – CTA reference added
2. Page iii – Ethics information removed
3. Page iii – Colleen Savage replaced as Intergroup Affairs Study Coordinator by Andrea Hiltz
4. Page 3 – Radiotherapy Quality Assurance added to list of Appendix B contents
5. Section 6.1.3.5 – Dose and volume objectives in Tables 3 and 4 changed
6. Section 10.3 – Reference to section 10.3 removed
7. Section 11.2 – Sentence added to remind that routine toxicities should be reported as SAEs
8. Section 11.4 - <CRF name> updated to Follow-up Form (CRF6) in Figure 4

14.1.2 AMENDMENTS MADE TO PROTOCOL VERSION 1.1 JUNE 2007

1. Page ii – Lillian Tsang replaced as Data Manager by Lindsey Masters
2. Page iii – Fred Saad added as a NCIC CTG Medical Expert
3. Section 1.1.1 – Explanation of three-way and two-way randomisation added
4. Section 1.1.3.2 – Information on free Eligard supply for patients in Canada removed
5. Section 4 – Prior hormone therapy removed from main entry exclusion criteria
6. Section 4 – Neoadjuvant treatment removed from main entry exclusion criteria
7. Section 4 – Hypogonadism removed from main entry exclusion criteria
8. Section 4 – Hormone therapy within previous 6 months added to main entry exclusion criteria
9. Section 4 – Radiotherapy Timing Randomisation exclusion criterion changed to more than 5 months since radical prostatectomy and a clarification that trial treatment should ideally start within 5 months after surgery added
10. Section 4 – Within 3 months after radical prostatectomy removed from Radiotherapy Timing Randomisation inclusion criteria
11. Section 4 – Hormone Duration Randomisation exclusion criteria changed from PSA > 10ng/ml to 5ng/ml
12. Section 5.2 – Randomisation CRF numbers updated to 1a, 1b, 2 and 3 or 4
13. Section 5.3 – Overall Summary of trial updated to include patients allocated to deferred RT with no PSA rise
14. Section 6.1.3.3 – Guidance added to title of treatment volumes section
15. Section 6.1.3.4 – Text changed to 'minimal dose to the PTV not less than…'
16. Section 6.2.2 – Eligard information for patients in Canada removed
17. Section 6.2.2.1 – Information on free Eligard supply for patients in Canada removed
18. Section 6.2.3 – Eligard information for patients in Canada removed
19. Section 6.2.3.1 – Information on free Eligard supply for patients in Canada removed
20. Section 7.1 – Numbering of Baseline Information Form changed to 1b
21. Section 7.1 – Patient History Form (CRF1b) added

14.1.3 AMENDMENTS MADE TO PROTOCOL VERSION 2.0 JANUARY 2008

1. Section 6.1.3.2 – Statement added to confirm that Intensity-Modulated Radiation Therapy (IMRT) may be used.
2. Section 6.1.3.3 – Statement added to clarify that treatment volumes are for guidance only.
3. Section 6.1.3.5 – Tables 3 & 4 – Dose volume objectives for the bladder have been updated as follows:

Dose	Volume Objective
50Gy	80%
60Gy	50%

4. Section 10.2 – Updated explanation of the role of the radiotherapy quality assurance (RTQA) group and requirements for the RTQA process.

14.1.4 AMENDMENTS MADE TO PROTOCOL VERSION 2.2 DECEMBER 2008

1. Page ii – Lindsey Masters replaced by Ben Spittle as Data Manager
2. Page ii – Gordana Jovic included as Statistician
3. Page iii – NCIC CTG funder renamed Canadian Cancer Society – Research Institute
4. Section 1.1 and throughout document – immediate radiotherapy now referred to as early radiotherapy and early salvage radiotherapy policy now referred to as deferred radiotherapy.
5. Section 1.1 and throughout document – 5 months changed to 22 weeks for clarity.
6. Section 1.1 – Clarification that patients joining the Radiotherapy Timing Randomisation and allocated to early radiotherapy are encouraged to but not required to also join the Hormone Duration Randomisation.
7. Section 1.1.3.2 – Clarification of the possibility to randomise between all three arms or two of the three arms of the Hormone Duration Randomisation including diagrams (Figure 3).
8. Section 4 – Patient inclusion and exclusion criteria
    i. Clarification that pre-operative hormone therapy within previous 6 months is an exclusion criterion
    ii. Addition of clarification that previous pre-operative hormone therapy for longer than 8 months is an exclusion criterion as is any post-operative hormone therapy
    iii. Addition of more specific inclusion criteria for Radiotherapy Timing Randomisation
    iv. Change of inclusion criterion from PSA ≤0.4ng/ml to ≤0.2ng/ml
9. Section 4.1 - Addition of Timing of Investigations table for Hormone Duration Randomisation and change of requirements for bone scans.
10. Section 5.1.1 – correction of typographical error – randomisation should be performed with five months after radical prostatectomy.
11. Section 5 – Removal of overall trial design Figure – now in Appendix A
12. Section 6.1 – Clarification that treatment with radiotherapy or hormone therapy will commence within two months of the randomisation.
13. Section 6.1.1 – Clarification that patients allocated to early radiotherapy in the Radiotherapy Timing Randomisation can also join the Hormone Duration Randomisation if they wish or the use of hormones can be decided by the investigator.
14. Section 6.1.3 – Additional clarification that radiotherapy should start within approximately two months of randomisation.
15. Section 6.1.3.3 – Clarification of formula to calculate % SV involvement risk.
16. Section 6.2 – Clarification that radiotherapy should ideally start within 2 months after randomisation and that radiotherapy should begin 2 months after hormone therapy.
17. Section 6.2 – Clarification that bicalutamide monotherapy is not approved for use in Canada.
18. Section 6.2.2 – Inclusion of degarelix as acceptable treatment for 6 months
19. Section 6.5 – Removal of sentence regarding study specific drug logs for study medication in Canada.
20. Section 13.1 – Inclusion of Canada in patient-reported outcomes study.

14.1.5 AMENDMENTS MADE TO PROTOCOL VERSION 3.0 OCTOBER 2009

1. Page i – Amendment made to show compliance with principles of GCP.
2. Page i – Sponsor address updated.
3. Page i – Danish and Irish sites included as MRC CTU sites.

4. Page ii – Clarification that Cancer Research UK and Medical Research Council are funding the trial in the UK.
5. Page ii - Ben Spittle replaced by Paul Patterson as Data Manager
6. Page ii – MRC CTU address updated.
7. Section 1.1 and throughout document – Radiotherapy Timing Randomisation and Hormone Duration Randomisation also referred to as RADICALS-RT and RADICALS-HD.
8. Section 1.1.4 – Outcome measures listed separately for RADICALS-RT and RADICALS-HD
9. Section 1.1.4.1 – RADICALS-RT primary outcome measure changed to freedom from distant metastasis and disease-specific survival added to list of secondary outcome measures.
10. Section 1.1.4.2 – Number of patients required for each randomisation amended.
11. Section 1.1.5 – Addition of up to 61/2 years of accrual and 6 to 7 years of follow-up.
12. Section 2.1 – Updated data from other sources added.
13. Section 2.2 – Updated data from other sources added.
14. Section 2.3 – Updated data from other sources added.
15. Section 2.4 – Updated data from other sources added.
16. Section 2.5 – Addition of information on other ongoing relevant studies and trials.
17. Section 4 – Clarification that RADICALS-RT randomisation should ideally be more than 4 weeks and less than 22 weeks after radical prostatectomy.
18. Section 4 - Clarification that patients joining short-term vs. long-term hormones comparison may have post-operative hormone therapy prior to randomisation but this must be discussed with the trial team.
19. Section 4.1 – 4 weeks changed to 30 days for clarity.
20. Section 4.1 – Clarification that bone scan is only required if Gleason score is ≥8 and post-operative PSA is detectable.
21. Section 5.1.1 – Clarification that RADICALS-RT randomisation should ideally be performed within 22 weeks of surgery.
22. Section 6.1 – Clarification that treatment section is guidance.
23. Section 6.2 – Clarification that treatment section is guidance.
24. Section 6.2.2 – Inclusion of degarelix as acceptable treatment in Canada.
25. Section 6.2.2.1 - Inclusion of degarelix as acceptable treatment in Canada.
26. Section 6.2.3 - Inclusion of degarelix as acceptable treatment in Canada.
27. Section 6.2.3.1 - Inclusion of degarelix as acceptable treatment in Canada.
28. Section 6.3 – Removal of progression whilst on therapy as a reason to stop trial treatments.
29. Section 6.7 – Addition of freedom from distant metastasis as a primary outcome measure.
30. Section 7.1 – Clarification that Comorbidity form (CRF2) should be completed two weeks prior to randomisation.
31. Section 7.2.2 – Addition of information about change of primary outcome measure for RADICALS-RT and clarification that bone scans are not mandated.
32. Section 9.1 – Addition of clarification that each comparison will have an independent randomisation programme.
33. Section 9.2.1 – Addition of information on new primary outcome measure for RADICALS-RT and reasons for change.
34. Section 9.2.3 – Addition of disease-specific survival as secondary outcome measure for RADICALS-RT only.
35. Section 9.3 – Updated information on sample size calculations for RADICALS-RT and RADICALS-HD and the comparisons in RADICALS-HD.
36. Section 9.5 – Statistical Analysis Plan information updated by moving information on planned comparisons in RADICALS-HD to new sections 9.3.3.1 and 9.3.3.2.
37. Section 9.6 – Addition of information on planned individual patient data meta-analyses.
38. Section 11.3 – CRF numbering corrected.
39. Section 15.3 – Updated information as IDMC has now been formed.

14.1.6 AMENDMENTS MADE TO PROTOCOL VERSION 4.0 JUNE 2011

This document was created using the MRC CTU Protocol Template Version 4.0, and as a result there are major formatting changes. The rationale to adopt this protocol template is to keep in line with the current style of documentation as recommended by the MRC CTU Protocol Review Committee. 'At UCL' has been added throughout protocol to reflect the organisational change.

1. Page i – Wording updated to reflect use of new protocol template.
2. Page i – Compliance section updated.
3. Page ii – Authorisations and Approvals section included.
4. Page ii – Trial Registration section included.
5. Page iii – Date of Ethics Approval added.
6. Page iii – Typographical correction: Regulatory approval date corrected from 17th April to 27th April.
7. Page iii – Email address has been amended.
8. Page iii – MRC CTU staff details updated.
9. Page iv – NCIC CTG contact details updated.
10. Section 1.1.1 – Typographical correction: space added between RADICALS-HD and alone.
11. Section 1.1.3.2 -Figure 3: Clarification added.
12. Section 1.1.5 – Years of further Follow-up updated to 7 years.
13. Section 4 – Table 1: Additional heading added.
14. Section 4.1 – Tables 2a and 2b merged as one table.
15. Section 4.1 – Table 2b: 4 weeks changed to 30 days for clarity and timing added.
16. Section 5.1.2 – Reference to see figures 1-4 corrected to figures 1-3.
17. Section 6.1 – Use of IMRT information added.
18. Section 6.1.3.4 – Updated to allow for other appropriate dose schedules.
19. Section 6.2.2 – Degarelix removed as an option for Canadian patients.
20. Section 6.2.2.1 – Degarelix removed as an option for Canadian patients.
21. Section 6.2.3 – Degarelix removed as an option for Canadian patients.
22. Section 6.2.3.1 - Degarelix removed as an option for Canadian patients.
23. Section 7.1 – Table 5: Two weeks prior to randomisation replaced by Within two weeks prior to randomisation for clarity.
24. Section 7.1 – Table 5: Randomisation forms details amended for clarity.
25. Section 7.5 – Details of national registries updated.
26. Section 7.6 – Trial closure information updated with current template.
27. Section 8.2 – Patient transfer procedure updated.
28. Section 9.2.3 – Requirement of radiotherapy treatment planning data added to treatment toxicity.
29. Section 9.3.2 – Clarification of number of patients radomised per month.
30. Section 10.2 – Typographical correction: hyphen added between RADICALS and RT.
31. Section 10.2 – 'the protocol' removed from last paragraph.
32. Section 10.2 – Information about data collection for RTTQA added.
33. Section 11.2 – Typographical correction: full stop added to end of 4th paragraph.
34. Section 11.3.2 (D) – Reporting period amended from one working day to 24 hours.
35. Section 11.3.2 – Notification procedure: Reporting period amended from one working day to 24 hours.
36. Section 11.4 - Reporting period amended from one working day to 24 hours.
37. Section 11.4 – Figure 4: Reporting period amended from one working day to 24 hours.

14.1.7 AMENDMENTS MADE TO PROTOCOL VERSION 5.0 MARCH 2014

Page i – Update to Canadian Sponsor name, Addition of information about sponsorship of the trial

2. Page ii – Update to MRC CTU address, update to MRC CTU staff

3. Page iii – Update to Canadian Sponsor name, update to CCTG staff

4. Section 1.1.3 – Update for Figure 1 and Figure 2
5. Section 1.1.4.A – Addition of ‘Freedom from biochemical progression’; Addition of ARTISTIC meta-analysis outcomes
6. Section 1.1.4.B – Addition of number of recruited patients
7. Section 1.1.5 –Update to trial duration
8. Section 1.1.7 – Clarification of samples to be collected
9. Section 2.3 – Change of Canadian sponsor name
10. Section 2.5.1 – Details of ARTISTIC meta-analysis
11. Section 3 – Addition of a Bullet Point for point 4
12. Section 5.2 - Update to Canadian Sponsor name
13. Section 6.1.1 – Addition of ‘entering into’ RADICALS-RT
14. Section 6.1.3.A – change Section 6.1.3.4 to Section 6.1.3.D
15. Section 7.2.2 – clarification of length of follow up
16. Section 7.5 – Addition of guidance for conducting visits with patients on long term follow-up
17. Section 7.5.1 – Addition of inclusion of ONS mortality data
18. Section 7.6 – Clarification of when trial closure will occur
19. Section 9.2.3 – Addition of ‘Freedom from distant metastasis (RADICALS-HD only); Addition of ‘Freedom from biochemical progression (RADICALS-RT only)
20. Section 9.2.4 – Addition of details of early reporting of biochemical outcomes
21. Section 9.6.1 – Update to the plans for ARTISTIC collaborative group
22. Section 10.5 – Addition of guidance for sites regarding protocol deviations
23. Section 11.3 – clarification of SAE reporting
24. Section 11.3.1 - clarification of adverse event reporting, update to link of CTCAE V3.0
25. Section 11.3.2.A – change Table 5 to Table 6
26. Section 11.3.2.C – Addition of how to find RSI for investigators, addition of responsibility of assigning expectedness
27. Section 11.3.2.D – Clarification of reporting timelines for SARs and SUSARs
28. Safety Reporting Flowchart – Updated
29. Section 12 – change website address

14.1.8 Amendments made to Protocol Version 6.0 December 2018

Protocol v6.0 was updated to Protocol v7.0 using the MRC CTU Protocol Template v7.0 and as a result there are some changes to sections and formatting. The use of this template is recommended by the MRC CTU Protocol Review Committee.

1. Title page – Canadian Cancer Trials Group logo updated
2. Title page – CTA number changed (following change to UCL Sponsorship)
3. General Information – Revised wording regarding ICH-GCP added to Compliance section
4. General Information – Clarification added regarding UCL Sponsorship and CCTG responsibilities
5. General Information – Grant reference numbers added to Funding section
6. General Information – Revised wording added to Authorisations and Approvals section
7. General Information – Clinical Centres information added
8. Information for MRC Investigators – Changes made to trial contacts and contact details
9. Information for MRC Investigators – Randomisation contact details removed
10. Pages 5-6 - Freedom from distant metastasis and Metastasis free survival added to Abbreviations
11. Pages 7-9 – Trial Summary transcribed into table format, number of participants to be studied and trial duration added
12. Page 12 – Primary outcome for RADICALS-HD changed to Metastasis-free-survival (MFS)
13. Page 13 – Trial Duration – Updated wording added regarding change in primary outcome for RADICALS-HD

14. Page 22 – Randomisation Contacts 5.2 removed, wording added to confirm randomisation closed.
15. Pages 27-28 – Protocol Treatment Discontinuation 4.3 section and relevant wording added
16. Page 31 – Early Stopping of Follow Up 5.5 – section and relevant wording added
17. Pages 31-32 – Loss to Follow Up 5.6 – clarification regarding follow up data added
18. Page 33 – Withdrawal from the trial completely (previously 8.1) – section removed, clarified in sections 4.3 and 5.6
19. Page 33 – Trial Closure 7.9 retitled End of Trial
20. Page 34-35 – Statistical considerations 6.2.2 - Wording added to clarify change to primary outcome to MFS for RADICALS-HD
21. Page 37 – Statistical considerations 6.3.3.A – Updated power calculations added for RADICALS-HD: No-HT vs RT+STHT
22. Page 38 – Statistical considerations 8.3.3.B – Updated power calculations added for RADICALS-HD: RT vs STHT vs RT+LTHT
23. Page 40 - Source Data section 7.4 and relevant wording added
24. Page 42 – Safety Reporting – 8.1 – 'Another important medical condition' and definition added to Table 6
25. Page 43 - Safety Reporting – 8.2.1 – Definition of Investigational Medicinal Product added
26. Page 43 – Safety Reporting – 8.2.2 – Examples of adverse events added
27. Page 43 – Safety Reporting – Clarification added regarding reporting of disease-related and elective surgery events
28. Page 44 – Safety Reporting – 8.3.2 – Investigator responsibilities clarification wording added
29. Page 45 – Safety Reporting – 8.3.3.B – Updated SAE causality definitions Table (7) added
30. Page 45– Safety Reporting – 8.3.3.C – Minor changes made to wording regarding assessing SAE expectedness
31. Page 45 – Safety Reporting – 8.3.3.D – Clarification wording added to Notification
32. Page 46 – Safety Reporting – 10.3.3.E – Clarification wording added regarding minimum SAE reporting criteria
33. Page 46 – Safety Reporting – 8.4 – Clarification wording added to CTU Responsibilities
34. Page 48 – Safety Reporting Flow chart – clarification wording added regarding SAEs exempt from expedited reporting and SAE notification procedures

14.2 APPENDICES

14.2.1 AMENDMENTS MADE TO APPENDIX A VERSION 1.0 MARCH 2007

1. Section Av – Heading spelling corrected from GUIDENCE to GUIDANCE
2. Section Avii – Colleen Savage replaced as Trial Manager by Andrea Hiltz and Chris Morash replaced as Urologist by Fred Saad.

14.2.2 AMENDMENTS MADE TO APPENDIX B VERSION 1.0 MARCH 2007

1. Section Bi – Model agreement for non-commercial research, GP letters and Accreditation Form I added to required documentation and requirements for radiotherapy quality assurance added.
2. Section Bii – Model agreement for non-commercial research added to contents of commitment form, telephone number updated and Investigators Statement, Contact Details Sheet and Delegation Log removed.
3. Section Biii – Radiotherapy quality assurance appendix added and numbering of subsequent sections changed.
4. Section Biv.2 – CTA reference added

14.2.3 AMENDMENTS MADE TO APPENDIX A VERSION 1.0 JUNE 2007

1. Section Av – Updated guidance flow diagram added
2. Section Avii – New trial committee members added

14.2.4 AMENDMENTS MADE TO APPENDIX B VERSION 1.1 JUNE 2007

1. Section Bii – Accreditation pack name changed to Site Specific Approval pack
2. Section Biii – Radiotherapy quality assurance guidance added
3. Section Bviii – Patient Information Sheet split into two sheets, one for patients entering both randomisations and one for the hormone duration randomisation only. Some minor wording changed and version changed to 3.0, January 2008
4. Section Bix – Consent Form split into two sheets, one for patients entering both randomisations and one for the hormone duration randomisation only. Consent statements added to clarify MRC CTU employees will have access to records, that name and NHS number will be taken and for participation in the quality of life study. Version changed to 2.0, January 2008.

14.2.5 AMENDMENTS MADE TO APPENDIX A VERSION 2.0 JANUARY 2008

1. Section Avi – Statement regarding non-participation of Canadian centres in the quality of life study removed.

14.2.6 AMENDMENTS MADE TO APPENDIX A VERSION 2.2 DECEMBER 2008

1. Section Ai – Addition of overall trial design figure.
2. Section Aiii – Removal of WHO performance status table as grading is not required for data collection.
3. Section Av – Updated flow diagram depicting criteria for suitable patients for each radiotherapy groups. This is reflected in the updated eligibility criteria in section 4 of the protocol.
4. Section Avii – Addition of Angela Lee to trial committee and Lindsey Masters replaced by Ben Spittle as Data Manager.

14.2.7 AMENDMENTS MADE TO APPENDIX B VERSION 2.1 DECEMBER 2008

1. Section Biv – Updated GP letters with amended wording and specification of which patient group each are applicable to. Version updated to v3.0.
2. Section Bv – SSA replaced with R&D approval.
3. Section Bviii – Patient Information Sheets amended to relate to each separate randomisation and introduction to section added.
4. Section Bix – Updated consent forms for each separate randomisation and introduction added.
5. Section Bix.2 – Removal of question 8 regarding quality of life study as this is not applicable to these patients.

14.2.8 AMENDMENTS MADE TO APPENDIX A VERSION 3.0 OCTOBER 2009

1. Section Av – Clarification of patient selection for RADICALS-RT
2. Section Av – Updated PSA value from >0.1 to >0.2.
3. Section Avi – Blank version number and date removed.
4. Section Avii – Trial Committee Members list updated

14.2.9 AMENDMENTS MADE TO APPENDIX B VERSION 3.0 OCTOBER 2009

1. Section Bii – MRC CTU address updated
2. Section Biii – MRC CTU address updated
3. Section Biv – Date and version of document added and date of letter space clarified.

4. Section Bviii – Date and version of patient information sheets updated.
5. Section Bviii – Information about sample size and recruiting countries updated.
6. Section Bix – Date and version of consent forms updated
7. Section Bix – Date and version of patient information sheets referred to updated.

14.2.10 Amendments made to Appendix A version 4.0 June 2011

1. Section Aiii – Urethral stricture removed from the RTOG toxicity table
2. Section Avii – Trial Committee Members list updated

17.2.11 Amendments made to Appendix B version 4.0 June 2011

1. Section Bii – MRC CTU trial email address updated
2. Section Biv – Date and version of GP letters updated
3. Section Bvi – Insurance arrangements have changed
4. Section Bviii – Patient Information Sheets have been removed from this appendix and will be kept as a separate document
5. Section Bix – Consent Forms have been removed from this appendix and will be kept as a separate document

14.2.11 Amendments made to Appendices version 5.0 March 2014

1. Appendix A, Section A VII – update of trial committee members
2. Appendix B, Section B II – update of MRC CTU contact address
3. Addition of Appendix C: Reference Safety Information

15 REFERENCES

1. Annual incidence of prostate cancer: http://info.cancerresearchuk.org/cancerstats/types/prostate/
2. Hospital Episode Statistics website: http://www.hesonline.nhs.uk
3. Jane Melia, Sue Moss, Louise Johns. Rates of prostate-specific antigen testing in general practice in England and Wales in asymptomatic and symptomatic patients: a cross-sectional study. BJU International 2005;94(1):51-6.
4. Atlas website: www.ices.on.ca. 2005.
5. Stephenson AJ, Shariat SF, Zelefsky MJ, Kattan MW, Butler EB, Teh BS et al. Salvage radiotherapy for recurrent prostate cancer after radical prostatectomy. JAMA 2004;291(11):1325-32.
6. Anscher MS. Salvage radiotherapy for recurrent prostate cancer: the earlier the better. JAMA 2004;291(11):1380-2.
7. Weigel, T, Bottke, D, Willich, N, and et al. Phase III results of adjuvant radiotherapy (RT) versus "wait and see" (WS) in patients with pT3 prostate cancer following radical prostatectomy (RP). Proc Am Soc Clin Oncol. 2005.
8. Bolla M, Van Poppel H, Tombal B, Vekemans K, Da Pozzo L, De Reijke TM, Verbaeys A, Bosser JF, Van Velthoven R, Collette L. 10-year results of Adjuvant radiotherapy after radical prostatectomy in pT2N0 prostate cancer (EORTC 22911). Proc 52nd Annual ASTRO Meeting 2010 Abs #62
9. Bolla M, van Poppel H, Collette L, van Cangh P, Vekemans K, Da Pozzo L et al. Postoperative radiotherapy after radical prostatectomy: a randomised controlled trial (EORTC trial 22911). Lancet 2005;366(9485):524-5.
10. Thompson IM, Tangen CM, Paradelo J, Lucia MS, Miller G, Troyer D, Messing E, Forman J, Chin J, Swanson G, Canby-Hagino E, Crawford ED. Adjuvant radiotherapy for pathological T3N0M0 prostate cancer significantly reduces risk of metastases and improves survival: long-term followup of a randomized clinical trial. J Urol 2009: 181(3):956-62
11. Wiegal T, Bottke D, Steiner U, Siegmann A, Golz R, Störkel S, Willich N, Semjonow A, Souchon R, Stöckle M, Rübe C, Weissbach L, Althaus P, Rebmann U, Kälble T, Feldmann HJ, Wirth M, Hinke A, Hinkelbein W, Miller K.J Clin Oncol. 2009 Jun 20;27(18):2924-30.
12. Morris SL, Parker C, Huddart R, Horwich A , Dearnaley D. Current opinion on adjuvant and salvage treatment after radical prostatectomy. Clin.Oncol.(R.Coll.Radiol.) 2004;16(4):277-82.
13. Lee LW, Clarke NW, Ramani VA, Cowan RA, Wylie JP, Logue JP. Adjuvant and salvage treatment after radical prostatectomy: current practice in the UK. Prostate Cancer Prostatic.Dis. 2005;8(3):229-34.
14. Bolla M, Collette L, Blank L, Warde P, Dubois JB, Mirimanoff RO et al. Long-term results with immediate androgen suppression and external irradiation in patients with locally advanced prostate cancer (an EORTC study): a phase III randomised trial. Lancet 2002;360(9327):103-6.
15. Pilepich MV, Winter K, John MJ, Mesic JB , Sause W, Rubin P et al. Phase III radiation therapy oncology group (RTOG) trial 86-10 of androgen deprivation adjuvant to definitive radiotherapy in locally advanced carcinoma of the prostate. Int.J Radiat.Oncol.Biol.Phys. 2001;50(5):1243-52.
16. Lawton CA, Winter K, Murray K, Machtay M , Mesic JB, Hanks GE et al. Updated results of the phase III Radiation Therapy Oncology Group (RTOG) trial 85-31 evaluating the potential benefit of androgen suppression following standard radiation therapy for unfavorable prognosis carcinoma of the prostate. Int.J Radiat.Oncol.Biol.Phys. 2001;49(4):937-46.
17. D'Amico A, Manola J, Loffredo M, Renshaw A, DellaCroce A, Kantoff P. 6-month androgen suppression plus radiation therapy vs radiation therapy alone for patients with clinically localized prostate cancer: a randomized controlled trial. Journal of the American Medical Association 2004;292(7):821-7.

18. Eulau SM, Tate DJ, Stamey TA, Bagshaw MA , Hancock SL. Effect of combined transient androgen deprivation and irradiation following radical prostatectomy for prostatic cancer. Int.J Radiat.Oncol.Biol.Phys. 1998;41(4):735-40.
19. King CR, Presti JC, Jr., Gill H, Brooks J, Hancock SL. Radiotherapy after radical prostatectomy: does transient androgen suppression improve outcomes? Int.J Radiat.Oncol.Biol.Phys. 2004;59(2):341-7.
20. Katz MS, Zelefsky MJ, Venkatraman ES, Fuks Z, Hummer A, Leibel SA. Predictors of biochemical outcome with salvage conformal radiotherapy after radical prostatectomy for prostate cancer. J Clin.Oncol. 2003;21(3):483-9.
21. RTOG Website. http://www.rtog.org/members/protocols/96-01/96-01.pdf. 2006.
22. Pilepich MV. Androgen suppression adjuvant to definitive radiotherapy in prostate carcinoma--long-term results of phase III RTOG 85-31. Int J Radiat Oncol Biol Phys. 2005;61(5):1285-90.
23. See W, Iversen P, Wirth M, McLeod D, Garside L, Morris T. Immediate treatment with bicalutamide 150mg as adjuvant therapy significantly reduces the risk of PSA progression in early prostate cancer. Eur.Urol. 2003;44(5):512-7.
24. Tyrrell CJ, 'Casodex' Early Prostate Cancer Trialists Group. Bicalutamide ('Casodex') 150 mg as adjuvant to radiotherapy in patients with localised or locally advanced prostate cancer: results from the randomised Early Prostate Cancer Programme. Radiotherapy and Oncology 2005;76(1):4-10.
25. Messing EM, Manola J, Sarosdy M, Wilding G, Crawford ED, Trump D. Immediate hormonal therapy compared with observation after radical prostatectomy and pelvic lymphadenectomy in men with node-positive prostate cancer. N Engl J Med 1999;341(24):1781-8.
26. Messing EM, Manola J, Yao J, Kiernan M, Crawford D, Wilding G et al. Immediate versus deferred androgen deprivation treatment in patients with node-positive prostate cancer after radical prostatectomy and pelvic lymphadenectomy. Lancet Oncol. 2006;7(6):472-9.
27. Hanks GE, Pajak TF, Porter A, Grignon D, Brereton H, Venkatesan V et al. Phase III trial of long-term adjuvant androgen deprivation after neoadjuvant hormonal cytoreduction and radiotherapy in locally advanced carcinoma of the prostate: the Radiation Therapy Oncology Group Protocol 92-02. J Clin.Oncol. 2003;21(21):3972-8.
28. Denham JW, Steigler A, Lamb DS, Joseph D, Turner S, Matthews J, Atkinson C, North J, Christie D, Spry NA, Tai KH, Wynne C, D'Este C. Short-term neoadjuvant androgen deprivation and radiotherapy for locally advanced prostate cancer: 10-year data from the Trans-Tasman Radiation Oncology Group 96.01 randomised controlled trial. Lancet Oncol 2011;12(5):451-9.
29. Schwarz R, Rexer H. Radiotherapy versus radiotherapy plus hormone therapy in the case of isolated PSA increase after radical prostatectomy. A prospective multicenter Phase III study of the AUO (AP 26/99) and the ARO (00/01. Urologe A. 2004;43(2):194-6.
30. Yokomizo A, Kawamoto H, Nihei K, Ishizuka N, Kakehi Y, Tobisu K et al. Randomised controlled trial to evaluate radiotherapy +/- endocrine therapy versus endocrine therapy alone for PSA failure after radical prostatectomy: Japan Clinical Oncology Group Study JCOG 0401. Jpn.J.Clin.Oncol. 2005;35(1):34-6.
31. Barthel FM, Royston P, Babiker A. A menu-driven facility for complex sample size calculation in randomized controlled trials with a survival or a binary outcome. The stata journal 2005;5(1):123-9.
